# Gene expression during zombie ant biting behavior reflects the complexity underlying fungal parasitic behavioral manipulation

**DOI:** 10.1186/s12864-015-1812-x

**Published:** 2015-08-19

**Authors:** Charissa de Bekker, Robin A. Ohm, Raquel G. Loreto, Aswathy Sebastian, Istvan Albert, Martha Merrow, Andreas Brachmann, David P. Hughes

**Affiliations:** Institute of Medical Psychology, Faculty of Medicine, Ludwig-Maximilians-University Munich, Goethestrasse 31, 80336 Munich, Germany; Department of Entomology and Department of Biology, Center for Infectious Disease Dynamics, Pennsylvania State University, University Park, State College, Pennsylvania, 16802 PA USA; Microbiology, Faculty of Science, Utrecht University, Padualaan 8, 3584 CH Utrecht, The Netherlands; CAPES Foundation, Ministry of Education of Brazil, Brasília, 70040-020 DF Brazil; Bioinformatics Consulting Center, Pennsylvania State University, University Park, State College, Pennsylvania, 16802 PA USA; Department of Biochemistry and Molecular Biology, The Huck Institutes of the Life Sciences, Pennsylvania State University, University Park, State College, Pennsylvania, 16802 PA USA; Faculty of Biology, Section Genetics, Ludwig-Maximilians-University Munich, Grosshaderner Strasse 2-4, 82152 Martinsried, Germany

**Keywords:** Parasite-host interactions, Extended phenotype, Behavioral manipulation, *Ophiocordyceps unilateralis*, *Camponotus castaneus*, Genomics, Mixed transcriptomics

## Abstract

**Background:**

Adaptive manipulation of animal behavior by parasites functions to increase parasite transmission through changes in host behavior. These changes can range from slight alterations in existing behaviors of the host to the establishment of wholly novel behaviors. The biting behavior observed in Carpenter ants infected by the specialized fungus *Ophiocordyceps unilateralis s.l.* is an example of the latter. Though parasitic manipulation of host behavior is generally assumed to be due to the parasite’s gene expression, few studies have set out to test this.

**Results:**

We experimentally infected Carpenter ants to collect tissue from both parasite and host during the time period when manipulated biting behavior is experienced. Upon observation of synchronized biting, samples were collected and subjected to mixed RNA-Seq analysis. We also sequenced and annotated the *O. unilateralis s.l.* genome as a reference for the fungal sequencing reads.

**Conclusions:**

Our mixed transcriptomics approach, together with a comparative genomics study, shows that the majority of the fungal genes that are up-regulated during manipulated biting behavior are unique to the *O. unilateralis s.l.* genome. This study furthermore reveals that the fungal parasite might be regulating immune- and neuronal stress responses in the host during manipulated biting, as well as impairing its chemosensory communication and causing apoptosis. Moreover, we found genes up-regulated during manipulation that putatively encode for proteins with reported effects on behavioral outputs, proteins involved in various neuropathologies and proteins involved in the biosynthesis of secondary metabolites such as alkaloids.

**Electronic supplementary material:**

The online version of this article (doi:10.1186/s12864-015-1812-x) contains supplementary material, which is available to authorized users.

## Background

Many parasites have evolved the ability to manipulate host behavior. This can range from alterations of existing behavioral traits to the expression of novel behaviors not present in the normal repertoire [1–3]. These changes in host behavior serve to promote completion of the parasite’s life cycle and are generally assumed to be the result of genes expressed by the parasite [3, 4]. Despite this, only a few studies have either demonstrated or implicated a genetic basis for manipulated behavior [5–12]. One example is insect infection by baculovirus where the virus induces an enhanced locomotory activity (ELA) in the caterpillar host prior to death [5, 11] combined with the migration of infected individuals towards elevated positions. At this elevated position, the host dies and liquefies, leading to virus particle transmission onto leaves that are then consumed by new caterpillar hosts [13–15]. ELA is a normal behavior in un-infected caterpillars, facilitating the search for suitable sites to undergo metamorphosis [16]. The baculovirus protein tyrosine phosphatase-encoding gene *ptp*, however, induces ELA upon infection [5, 11]. A second mechanism involves expression of the baculovirus gene ecdysteroid uridine 5’-diphosphate (UDP)-glucosyltransferase (*egt*) that results in a disrupted climbing behavior [9]. Healthy caterpillars climb at night for feeding purposes and return down during the day to avoid predation. Upon infection, the retreat is disrupted due to *egt* expression. Despite the identification of these genes, the host pathways through which behavior is manipulated are still unknown. The attraction of infected individuals by light, however, suggests a role for host pathways involved in phototaxis and light perception [17].

The progress made in the baculovirus system does not necessarily provide us with answers that can be extrapolated to other systems. This is especially true in host-parasite systems where more complex manipulations are observed, which lead to manipulated hosts expressing wholly novel behaviors. One such example involves the fungal parasite *Ophiocordyceps unilateralis sensu latu (s.l.)* manipulating brains of Carpenter ants (genus *Camponotus*). In this system, a set of stereotyped behaviors is induced once the fungal colony growing inside the host reaches a sufficient size. Infection induced behaviors include leaving the nest at a different time of day compared to regular foraging, non-directed movements, convulsions and climbing up the vegetation [10]. Subsequently, the host bites into the vegetation at an elevated position and dies [18]. This manipulated biting is not part of the ant’s normal behavior and assists the formation of the fruiting body and spore transmission post-mortem [18–20]. The place of biting is again stereotyped and seemingly adapted to the ecosystem in which the infection takes place: infected ants bite the main veins or margins of leaves in rainforests [10, 18, 21, 22] while they bite twigs in temperate forests [12]. Moreover, the transition from wandering to this death grip is synchronized to a certain time of day [10, 12].

Here, we describe the gene expression in the complex manipulator *O. unilateralis s.l.*, a fungal isolate collected from the temperate forests of South Carolina [12], and its *Camponotus* ant host during the *in vivo* manipulated biting event. We performed a mixed transcriptomics study on the heads of experimentally infected individuals sampled during and after manipulated biting. We also sequenced and annotated the genome of *O. unilateralis s.l.* from North America. We found that during manipulated biting, the fungal parasite *O. unilateralis s.l.* up-regulates genes that putatively encode for proteins involved in oxidation-reduction processes and pathogenicity-related interactions, some of which may have medical or industrial applications. Moreover, we have identified genes that are involved in the expression of putative proteins that might affect host behavior. In the ant host, we found the differential expression of genes seemingly involved in apoptosis, immune and stress responses, as well as possible targets of behavioral manipulation.

## Results and discussion

### Apparent synchronization of manipulated biting behavior

We used an *O. unilateralis s.l.* species from South Carolina and its natural host *Camponotus castaneus* to study behavioral manipulation of the host by the parasite. Ants were experimentally infected through injection and kept under 24 h light: dark (12 h: 12 h) and temperature cycles together with sham-treated (injected with media without fungal material) and untreated individuals (see Methods). Only infected ants that died between 16 and 24 days post infection were observed in the characteristic manipulated biting position, as illustrated with pictures and videos in [12]. Manipulated biters were always found in this position during the first observational recording of the day at 09:00 h local time (3 h after lights on). The body and the legs would still be moving and twitching, an indication that the ant was alive. These ants would not react to any environmental stimuli (e.g., agitation, other ants). At 13:00 h, movements were reduced to occasional twitching of the legs. At 14:00 h no movement was detected, suggesting that the ant host had died. Similar observations were made in independent experiments with this parasite and host species (e.g., in [12]). The consistent observations of time of death imply that both, manipulated biting behavior and the subsequent death, are synchronized. Similar synchronized manipulation and death was observed in another species of ant-manipulating *Ophiocordyceps* from Thailand. However, in that system the infected ants displayed manipulated biting behavior around solar noon, followed by death 6 h after the biting event had taken place [10]. The shift of synchronized timing of biting towards the early morning/late night in our experiments could be an effect of the set-up (e.g*.*, differences in temperature and light compared to nature), or due to species-specific timing of manipulated behavior. Future work to establish the timing of manipulation under field conditions in the temperate woods of South Carolina would be helpful.

Live ants displaying manipulated biting behavior were collected at 10:00 h. At 14:00 h, biting ants that showed no movement and thus appeared dead were harvested. This time point is referred to as “after manipulation”. To decrease within-sample variation possibly introduced by collecting manipulated ants across different days, three individuals that were found manipulated on the same day were used for each sample type. Healthy control ants were also collected at 10:00 h and 14:00 h. As a baseline for *O. unilateralis* gene expression during and after manipulation, fungal cultures kept in insect cell culture media were harvested.

### General genome features of *O. unilateralis s.l.* and *C. floridanus*

As a reference for the *C. castaneus* RNA-Seq reads, the published genome of a related *Camponotus* species, *Camponotus floridanus*, was used [23]. For the present study, we repeated the functional annotations for the gene predictions using the same methodology as for *O. unilateralis s.l.*. This resulted in statistics similar to the ones previously reported: a total genome size of 234.88 Mb encoding 17,061 genes of which 10,015 were predicted to encode proteins within 4,229 unique PFAM domains and of which 4.1 % is putatively being secreted. The CEGMA completeness for this genome was 99.3 %.

The genome of the *O. unilateralis s.l.* from South Carolina [12], was sequenced to 120-fold coverage. This genome served as a reference for the fungal RNA-Seq reads in our samples. Contig assembly resulted in a genome size of 26.05 megabases (Mb). Gene prediction yielded 7,831 putative genes. By using the Core Eukaryotic Genes Mapping Approach (CEGMA) core genes dataset [24, 25], the *O. unilateralis s.l.* genome was estimated to be 98.7 % complete. PFAM domains were assigned to 5,556 (71 %) of these genes, and there were 3,498 unique predicted PFAM domains [26]. The proportion of genes encoding putatively secreted proteins was 11.4 %. A Gene Ontology (GO) annotation was assigned to 49.9 % of the genes and 1,800 (23 %) received a Kyoto Encyclopedia of Genes and Genomes KEGG) annotation (Table 1). From these 1,800 genes, 45.9 % were found to be involved in various forms of metabolism and 36.6 % in genetic information processing such as transcription, translation and protein folding (Additional file 1).

**Table 1:** *Ophiocordyceps unilateralis s.l.* genome assembly and annotation statistics

Property	Value
Scaffolds in assembly	7875
Total assembly length (Mbp)	26.05
N50 (kb)	22
Largest scaffold (kb)	145
Assembly GC content (%)	54.76
Assembly gaps (%)	0.73
Repetitive content (%)	7.81
Genes	7831
Gene length (median)	1420
Transcript length (median)	1275
Exon length (median)	261
CDS length (median)	1272
Protein length (median)	424
Spliced genes (total, %)	6038 (77.1 %)
Exons per gene (median)	3
Intron length (median)	62
Introns per spliced gene (median)	3
Gene density (genes/Mbp)	300.57
Proteins with internal stops (total, %)	0
Unique PFAM domains	3498
Genes with PFAM (total, %)	5556 (70.95 %)
Genes with GO (total, %)	3904 (49.85 %)
Genes with signalP (total, %)	891 (11.38 %)
Genes with TMHMM (total, %)	1493 (19.07 %)
CEGMA completeness (%)	98.69

**Fig. 1:**
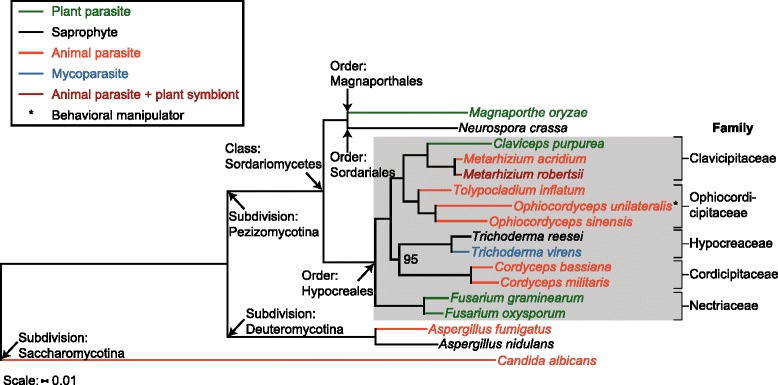
Phylogenetic relationship of *O. unilateralis s.l.* with other ascomycetous fungi. Phylogenetic reconstruction of *Ophiocordyceps unilateralis* and an outgroup of Ascomycota. The order Hypocreales is highlighted in grey. The tree was rooted on *Candida albicans* and this branch is not drawn to scale. Bootstrap values were 100, unless indicated otherwise. Fungal lifestyles are indicated with different colors

**Fig. 2:**
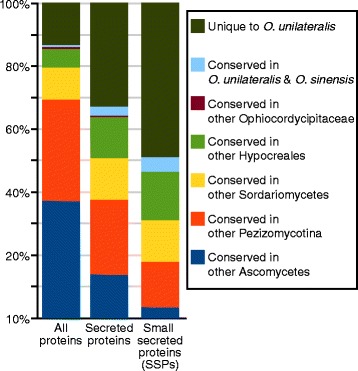
Conservation of *O. unilateralis s.l.* genes in other Ascomycota. Bar charts displaying the percentage of genes encoding all proteins, secreted proteins and small secreted proteins that were found to be conserved across taxonomic groups or to be unique to the *O. unilateralis s.l.* genome

The genome and gene predictions of *O. unilateralis s.l.* were compared to that of 16 published Ascomycota genomes. A phylogenetic reconstruction based on 168 single-copy genes that were conserved across the compared genomes is depicted in Fig. 1. The tree is congruent with a previously published phylogeny that contained a subset of the presently used organisms [27]. Fungi with various lifestyles both within and outside the class Sordariomycetes served as an outgroup to the order Hypocreales in our phylogenomic analysis. The order Hypocreales harbors a variety of insect parasites, (including *O. unilateralis s.l.*) distributed across several fungal families, as well as taxa that infect plants and other fungi (mycoparasites). We also assessed the conservation of the protein-encoding genes of *O. unilateralis s.l.*. This revealed that 13 % of all putative proteins within the genome are unique to *O. unilateralis s.l.*, with 6 % conserved only in fungi within the order Hypocreales, and 1 % shared only with *Ophiocordyceps sinensis*. When we compared only those proteins that have a predicted secretion signal (SignalP annotation), 33 % (=293 secreted proteins) were found to be unique to the *O. unilateralis s.l.* genome. Of these, 146 were predicted to be small secreted proteins (SSPs). This is 49 % of all SSPs found in the *O. unilateralis s.l.* genome, which suggests that half of the putatively bioactive SSPs in this fungal parasite are unique to this species complex (Fig. 2). Both this set of unique SSPs and SSPs in general have a higher percentage of cysteine residues (2.4 % and 2.8 %, respectively), compared to the full protein set (1.4 %). This is frequently observed for SSPs in fungal genomes, and the cysteine residues likely play a role in disulphide bonds [28, 29].

We characterized the predicted secreted protein set further. An impressive number of 34 genes were found that contained one or more putative enterotoxin_a PFAM domains. These are bacterial-like toxins of which 9 are homologs of cholera toxins and the rest are homologs of heat-labile enterotoxin_a. These toxins are not commonly found in fungal genomes [30]. Of the 17 fungi analyzed here, 9 did not have any enterotoxin-encoding genes in their genomes. Only the insect-infecting fungi and plant pathogen *M. oryzae* contained genes putatively encoding these bacterial-like toxins. The *Tolypocladium inflatum* genome was found to have 3 enterotoxin-encoding genes, *Cordyceps militaris* has 4, *Magnaporthe oryzae* has 6, *Metarhizium acridium* has 7, *Ophiocordyceps sinensis* has 8, *Cordyceps bassiana* has 14, and *Metarhizium robertsii* has 16. The *O. unilateralis s.l.* genome contained at least twice as many toxins relative to the other fungi within the comparison. A phylogenetic analysis showed that these enterotoxins are diversely distributed displaying resemblance with enterotoxins from other species. This indicates that the high number of enterotoxins in the genome of *O. unilateralis* are not a result of a duplication of a small number of enterotoxins, but rather a general expansion of phylogenetically diverse enterotoxin genes (Additional file 2). In weevils (a beetle), it has been shown that bacterial enterotoxins reduced the production of chemosignaling molecules by affecting the alcohols in these compounds [31, 32]. Communication in social insects generally involves chemical signals. As such, ants have an exocrine apparatus that is mainly used for this purpose [33]. The expansion of genes putatively encoding for secreted enterotoxins in the *O. unilateralis s.l.* genome might be an indication that impairing host communication through chemosignaling is one of the mechanisms employed by this manipulative fungus.

### General mixed transcriptome features of *O. unilateralis s.l.* and *C. castaneus*

Gene expression profiles of *O. unilateralis s.l.* and *C. castaneus* during (10:00 h) and after manipulated biting (14:00 h) were generated through mixed transcriptomics on entire ant heads. We are therefore looking at the gene expression of interactions between the fungal parasite and the ant brain, as well as other tissues inside the head (e.g., muscles and fat body tissue). Gene expression studies on entire *Drosophila* heads however demonstrate that a good resolution of the processes in the brain can still be had this way [34]. Fungal cultures grown in insect cell culture media, and heads of healthy ants collected at the respective times of day for the mixed transcriptome samples, were used as controls. Sequencing yielded an average of 17.5 million paired reads per sample with an average mean quality score of 35 (see Additional file 3 for statistics). These reads were mapped to the *O. unilateralis s.l.* and *C. floridanus* reference genomes to obtain expression profiles, which were normalized to Fragments Per Kilobase of exon model per Million fragments mapped (FPKM) (see Methods). Of the *O. unilateralis s.l.* genes, 86.0, 86.5 and 85.0 % were considered transcriptionally active with an expression level of at least 4 FPKM in the fungal control culture, during manipulation, and after manipulation, respectively. Of the *C. floridanus* homologs, 50.1 and 49.6 % were considered transcriptionally active in *C. castaneus* controls compared to 50.0 and 49.8 % in infected *C. castaneus* ant heads during and after manipulated biting behavior, respectively. Pairwise analysis was used to determine which genes were significantly (Q < 0.05) and at least 2-fold up- or down-regulated between sample conditions (Additional file 4).

For the ant host, an average of 27.3 % of the reads from the healthy control samples, 14.6 % of the mixed “manipulated biting behavior” reads and 9.0 % of the mixed “after manipulation” reads mapped to the *C. floridanus* genome (Additional file 3). The similarity/divergence of the *C. castaneus* and the *C. floridanus* genomes is unknown, which could be the reason for the lower percentage of reads mapping to the reference genome. Despite this low mapping score for ant reads, we did find 1,891 genes that were differentially expressed. This is considerably more compared to what has been found in previous work on gene expression of ant hosts challenged with pathogenic fungi (375 genes) [35]. For the fungal parasite, an average of 81.4 % of the control sample reads, 46.9 % of the mixed “manipulated biting behavior” reads and 61.1 % of the mixed “after manipulation” reads mapped to the *O. unilateralis s.l.* genome. A relative comparison of the mixed transcriptome mapping scores to those of the single transcriptomes suggests that during manipulated biting behavior only about half of the tissue inside an infected ant head was made up by the ant host (27.3 % of the control sample reads vs. 14.6 % of the “after manipulation” reads mapping to the *C. floridanus* genome). The other half consisted of fungal cells (81.4 % of the control sample reads vs. 46.9 % of the “after manipulation” reads mapping to the *O. unilateralis s.l.* genome). After manipulation had taken place, the fungal colony had grown to make up about 75 % of the biological material inside an infected ant’s head (Additional file 5). An alternative explanation is that the expression levels considerably decreased in the dying ant, which causes the relative expression of the fungus to increase.

### Enrichment of functional annotations in *O. unilateralis s.l.* differential gene expression

To obtain insight into the biological processes that are different between samples, we performed an enrichment analysis of functional annotations among differentially expressed genes. We determined which functional annotation terms were significantly overrepresented among various subsets of differential expression (Additional files 6 and 7 ) by performing the Fisher Exact test (corrected P < 0.05). Within the 1,417 genes that were up-regulated in *O. unilateralis s.l.* during the manipulated biting (10:00 h) compared to fungal control cultures, those involved in DNA binding, DNA replication and DNA repair, and oxidation-reduction processes were overrepresented (Additional file 6: Table S1). Genes involved in DNA replication and DNA repair were also overrepresented in the 1,050 up-regulated fungal genes after manipulated biting had taken place (14:00 h) compared to control cultures (Additional file 6: Table S3).

Genes involved in the metabolism of sugars were generally down-regulated during manipulation (Additional file 6: Table S2). However, a direct comparison between gene expression in *O. unilateralis* during (10:00 h) and after manipulation (14:00 h) indicated an overrepresented up-regulation of carbohydrate processing genes after manipulation had taken place (Additional file 6: Table S5). Even when the data set was narrowed down to only those genes that were first down-regulated during manipulation before being up-regulated again afterwards, carbohydrate metabolic processes were still overrepresented (Additional file 6: Table S8). This suggests that the ants collected at 14:00 h indeed represent the situation after manipulation in which the host has presumably been killed and the fungus has transitioned to actively consuming the host (i.e., saprophytic growth). Following death, rapid growth occurs as the fungus switches from growing within the host to growing externally, forming the stalk needed for reproduction [18]. Coinciding with this, genes with a pathogenesis GO annotation term were overrepresented in the down-regulated gene set after manipulation had taken place (Additional file 6: Table S4). All pathogenesis-related genes in this subset were annotated as protein exotoxins with either a functional enterotoxin_a domain (all but one) or a *Pertussis* S1 subunit domain (Additional file 7, sheet 4). As discussed above, such exotoxins are normally secreted by bacteria but have also been reported to be present in fungal genomes such as *C. bassiana* [30].

Our transcriptomics data thus indicate a shift from parasitic to saprophytic activity. This is supported by previous metabolomics analyses, which demonstrated that fungal parasites display vastly different secretomes during growth on live versus dead ant tissues [36]. Related to this shift we found that after manipulation, *O. unilateralis s.l.* had down-regulated an overrepresented amount of genes involved in oxidation-reduction processes, such as cytochrome P450s (CYPs) (Additional file 6: Table S6). One of the many roles CYPs play involves the biosynthesis of secondary metabolites related to pathogenesis [37]. This result still holds when the subset of genes is reduced to only those that were first significantly up-regulated during manipulation (about 60 % of this subset, Additional file 6: Table S7). Genes with a secondary metabolism annotation term were also down-regulated in overrepresented manner after manipulation, when compared to expression during biting. Among them were genes putatively involved in the metabolism of neurotoxic compounds such as alkaloids and indole diterpene alkaloids (penitrems), and genes involved in the production of fungal bioactive compounds such as nonribosomal peptide synthetases (NRPSs) and polyketide synthases (PKSs) (Additional file 7, sheet 7).

When examining the subsets for differential expression we found an overrepresentation of genes predicted to encode secreted proteins in almost all of them. When comparing gene expression during manipulation with that of the fungal control culture, 13.5 and 14.7 % from the total of the up- and down-regulated genes, respectively, were annotated to have a secretion signal. This is, respectively, 21.4 and 19.0 % of all genes predicted to encode secreted proteins (Additional file 6: Tables S1 and S2). The fungus *O. unilateralis s.l.* thus likely secretes a different array of compounds inside the ant at the time of manipulated biting, compared to when it is grown in insect cell culture media. Similarly, about 20 % of all secreted protein encoding genes was down-regulated after manipulation had taken place, both when compared to the fungal control culture and to gene expression during manipulation (Additional file 6: Tables S4 and S6). This suggests that, again, after manipulation, the fungal parasite dynamically changes its secretion profile. Our findings are in agreement with previous work using a metabolomics approach to determine the secretomes of fungal entomopathogens (including *O. unilateralis s.l.*) in insect cell culture media with and without ant tissues [12, 36]. About one third of the up- and down-regulated genes encoding secreted proteins were annotated as SSPs (Additional file 7). SSPs are often highly species-specific, lacking similarity to known proteins, making functional predictions difficult. Several SSPs that have been experimentally characterized are involved in parasitic relationships of fungi with their hosts, affecting cellular processes inside the host [38, 39]. This suggests that *O. unilateralis s.l.* not only has a specific secretion profile during behavioral manipulation, it also tailors its array of small secreted effector proteins.

**Fig. 3:**
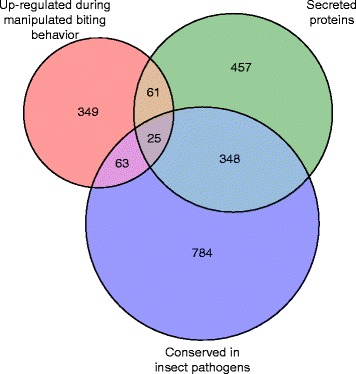
VENN diagram of *O. unilateralis s.l.* genes that are putatively involved in insect pathogenesis and establishing manipulated biting behavior in the ant *C. castaneus.* Such genes are likely to be specifically up-regulated during manipulated biting behavior, when compared to growth in pure culture and in ants after manipulation has taken place. This diagram shows the overlap between i) these up-regulated genes, ii) the *O. unilateralis s.l.* genes that putatively encode secreted proteins, and iii) genes that are conserved in other insect pathogens. A total of 2087 genes have at least one of these characteristics. The area size is approximately congruent with the amount of genes that make up the overlapping and non-overlapping groups

Since secreted proteins were generally overrepresented, we also investigated their conservation across the entomopathogenic (insect-infecting) fungi within the order Hypocreales. From all the genes that were annotated to have a secretion signal, about 42 % were conserved among the fungi with an insect parasitic lifestyle. A little over half of the secreted proteins was thus to be found only in the *O. unilateralis s.l.* genome. Of this 58 %, about 12 % was up-regulated solely during manipulated biting behavior, while this was only 6.7 % for the conserved putative secretion proteins. In fact, the majority (82 %) of the genes found up-regulated during manipulated biting behavior and down-regulated again after, were not conserved in other Hypocrealean entomopathogens (Fig. 3). This suggests that *O. unilateralis s.l.* might be using a rather unique set of genes to establish manipulated biting behavior, compared to other non-manipulating insect-infecting fungi.

### Up-regulation of alkaloid biosynthesis during manipulated biting behavior

In general, behavior-manipulating parasites are thought to change host behavior by means of secreting neuromodulatory agents near both the central and peripheral nervous systems of their hosts [40]. Secondary metabolites, such as polyketides, nonribosomal peptides and alkaloids, have been suggested to play a role in this [41]. We hypothesized that genes encoding for secreted candidate neuromodulators, and proteins involved in the biosynthesis of neuromodulating secondary metabolites would be up-regulated during manipulated behavior, and likely down-regulated again afterwards (gene subset in Additional file 8, sheet 2). We indeed found PKSs and NRPSs with this expression pattern, as well as proteins involved in the production of various alkaloids (summarized in Table 2). For example, two tryptophan dimethylallyltransferases within the *O. unilateralis s.l.* genome were highly expressed (426 and 938 FPKM) during manipulated biting behavior while transcription was non-existent in the fungal baseline culture. Their expression also significantly dropped again after manipulation had taken place and the ant host had died (Additional file 8, sheet 2). Tryptophan dimethyltransferases catalyze the first rate-limiting step of ergot alkaloid biosynthesis of which tryptophan is the biochemical precursor [42, 43]. Indeed, a gene putatively encoding for tryptophan synthase followed a similar expression pattern suggesting accumulation of the precursor during manipulation as well. Moreover, 2 putative indoleamine 2,3-dioxygenases, which degrade tryptophan, were significantly down-regulated at this time (Additional file 7, sheet 3). Ergot alkaloids are well-known to affect animal behavior. They are a class of indole alkaloids, which resemble neurotransmitters such as serotinin, noradrenaline and dopamine and can therefore act on the central and peripheral nervous systems as agonists of various receptors [44]. *Claviceps purpurea,* a related fungus infecting rye grasses, produces these compounds [42]. The ergot in these infected grasses has been known to cause serotonergic overstimulation of the Central Nervous System in humans and livestock that consumed infected rye, which can lead to e.g., muscle spasms and hallucinations [45]. This suggests that the putative production of ergot-like alkaloids by *O. unilateralis s.l.*, through the up-regulation of genes involved in their metabolism, could be affecting the brains of Carpenter ants in similar fashion.

**Table 2:** Candidate *O. unilateralis s.l.* neuromodulators

Protein ID	Functional annotation	Fold up during manipulation	Fold down death after manipulation
Secondary metabolism			
Ophio1_1|g767.t1	Putative tryptophan dimethyltransferase involved in metabolism of ergot alkaloids, which resemble indole neurotransmitters and are therefore agonists of various neuroreceptors	Infinite	40.46
Ophio1_1|g3491.t1	Geranylgeranyldiphosphate transferase, PaxC homolog involved in metabolism of indole diterpenes (penitrems) causing tremors through big potassium channel inhibition	18688.55	25.57
Ophio1_1|g3493.t1	Geranylgeranyl pyrophosphate synthase involved in metabolism of terpenoids and polyketides	14705.25	18.60
Ophio1_1|g3831.t1	Putative PKS	4313.78	4.85
Ophio1_1|g3492.t1	Integral membrane protein, PaxB homolog involved in metabolism of indole diterpenes (penitrems) causing tremors through big potassium channel inhibition	3445.96	16.75
Ophio1_1|g3487.t1	Flavin-dependent monooxygenase, PaxM homolog involved in metabolism of indole diterpenes (penitrems) causing tremors through big potassium channel inhibition	3015.11	39.72
Ophio1_1|g3485.t1	Putative tryptophan dimethyltransferase involved in metabolism of ergot alkaloids, which resemble indole neurotransmitters and are therefore agonists of various neuroreceptors	2293.83	28.51
Ophio1_1|g768.t1	NRPS-like protein	374.06	19.74
Ophio1_1|g444.t1	Homolog of P450 involved in fusarin C metabolism cluster	147.52	12.25
Ophio1_1|g4784.t1	Putative NRPS	6.14	8.84
Ophio1_1|g3877.t1	Putative NRPS	6.03	3.77
Ophio1_1|g5818.t1	Putative histidinol-phosphate aminotransferase involved in metabolism of tropane alkaloid scopolamine, which has an antagonist effect on neuroreceptors	2.91	1.17
Ophio1_1|g1532.t1	Acyl-hydrogenase involved in metabolism of terpenoids and polyketides	2.52	2.28
Secreted proteins			
Ophio1_1|g67605.t1	SSP, putative lipocalin, ApoD homolog, lipophilic protein binding capacity, involved in brain neuropathologies	Infinite	17.39
Ophio1_1|g209.t1	SSP, no functional annotation	Infinite	124.98
Ophio1_1|g2647.t1	SSP, no functional annotation, unique to *O. unilateralis s.l.*	Infinite	74.44
Ophio1_1|g2296.t1	SignalP, putative bacterial-like enterotoxin involved in pathogenesis and possibly affecting chemosensory compounds	3263.32	199.43
Ophio1_1|g4844.t1	SignalP, putative sphingomyelin phosphodiesterase, ASM, SMPD1 homolog involved in sphingolipid metabolism and therefore cell regulation	1506.00	7.06
Ophio1_1|g5103.t1	SSP, contains ankyrin-repeat, unique to *O. unilateralis s.l.*	316.29	3.24
Ophio1_1|g5079.t1	SSP, no functional annotation	118.50	3.23
Ophio1_1|g3495.t1	SSP, no functional annotation, unique to *O. unilateralis s.l.*	113.50	2.91
Ophio1_1|g364.t1	SignalP, putative protein-tyrosine-phosphatase, involved in ELA behavior induction in Lepidoptera by baculoviruses	111.28	5.39
Ophio1_1|g4465.t1	SSP, putative kynurenine formamidase involved in tryptophan metabolism towards quinolinic acid and kynurenic acid	102.17	6.26
Ophio1_1|g734.t1	SSP, no functional annotation, unique to *O. unilateralis s.l.*	85.48	19.28
Ophio1_1|g6878.t1	SSP, no functional annotation	59.57	3.82
Ophio1_1|g1605.t1	SSP, no functional annotation	51.39	11.53
Ophio1_1|g3218.t1	SSP, no functional annotation	29.14	2.92
Ophio1_1|g3121.t1	SSP, no functional annotation	20.03	2.84
Ophio1_1|g6661.t1	SSP, no functional annotation, unique to *O. unilateralis s.l.*	17.31	7.61
Ophio1_1|g1154.t1	SSP, no functional annotation, unique to *O. unilateralis s.l.*	17.02	2.53
Ophio1_1|g2359.t1	SSP, no functional annotation, unique to *O. unilateralis s.l.*	12.19	3.56
Ophio1_1|g749.t1	SignalP, P450, CYP5A ortholog, putative thromboxane-A synthase regulating inflammation levels	10.39	6.36
Ophio1_1|g103.t1	SignalP, tyrosinase involved in dopamine metabolism, melanin production and quinone production	8.70	21.89
Ophio1_1|g7417.t1	SSP, no functional annotation	8.45	2.43
Ophio1_1|g3227.t1	SSP, no functional annotation, unique to *O. unilateralis s.l.*	6.90	3.36
Ophio1_1|g5581.t1	SSP, no functional annotation, unique to *O. unilateralis s.l.*	4.64	17.27
Ophio1_1|g25.t1	SSP, no functional annotation, unique to *O. unilateralis s.l.*	3.72	2.97
Ophio1_1|g999.t1	SignalP, tyrosinase involved in dopamine metabolism, melanin production and quinone production	2.05	6.12

Tryptophan synthesis in turn involves the precursors anthranilate and indole-3-glycerol-phosphate [46]. The latter can also function as an indole donor to synthesize indole diterpene alkaloids, such as the fungal compound paxilline [47, 48]. Several genes putatively encoding enzyme homologs of the *pax* pathway (i.e., the biosynthesis pathway of fungal indole diterpenes) were found to be highly expressed in *O. unilateralis s.l.* during the manipulated biting event, again followed by a significant down-regulation after manipulation had taken place (Additional file 7, sheet 7). A putative geranyldiphosphate transferase (PaxC homolog) was found, which catalyzes the first step in this pathway. In addition, a flavin-dependent monooxygenase (PaxM homolog) and an integral membrane protein (PaxB homolog) were found, which are required for the following oxidative cyclization steps resulting in a wide variety of indole diterpenes and their precursors paspaline and emindole DA [49, 50]. These metabolites, also called penitrems, can have a variety of biological activities, including the modulation of insect and animal ion channels and insect feeding deterrence [51, 52]. Furthermore, they can inhibit big potassium (BK) channels, which regulate processes such as passive smooth muscle contraction and neuron excitation causing tremors (*i.e.* unintentional rhythmic muscle movement) [53]. A continuous rhythmic moving of the legs of manipulated twig-biting individuals was observed (in this study as well as [10] and [12], additional videos), which indicates that the putative penitrem production by *O. unialteralis s.l.* might have a tremorgenic effect in ants as well.

Since tryptophan can also function as the precursor for many other classes of alkaloids (KEGG Ontology Pathway 00400) we searched for more differentially expressed genes involved in tryptophan metabolism. Among the genes involved in tryptophan metabolism that were significantly up-regulated both during and after manipulation we found a histidinol-phosphate aminotransferase (Additional file 7, sheet 2 and 4). KEGG Orthology indicated that this enzyme could be involved in the biosynthesis of the tropane alkaloid scopolamine. This compound is an antagonist of the muscarinic acetylcholine receptor, which binds the neurotransmitter acetylcholine (reviewed in [54]). Similarly, the putative production of tropane alkaloids by *O. unilateralis s.l.* could have an antagonist effect on neurotransmitter receptors within the membranes of ant brain neurons.

### Differentially expressed *O. unilateralis s.l.* genes with secretome annotations

**Fig. 4:**
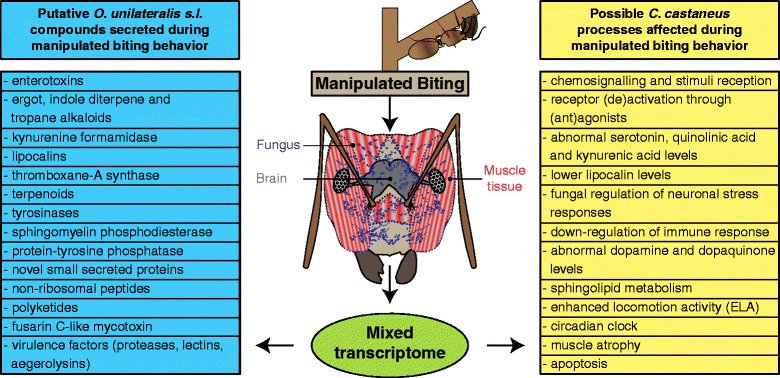
Infographic summarizing the putatively secreted *O. unilateralis s.l.* compounds and possible *C. castaneus* processes found in this study that are seemingly involved in manipulation

In almost all subsets for *O. unilateralis s.l.* genes that were differentially expressed during and after manipulated biting behavior, those that were annotated to have a secretion signal were overrepresented. As previously stressed, this indicates that this parasitic fungus dynamically changes a significant part of its secretome from the time of manipulation to host death a few hours after manipulation (Additional file 9). During manipulated biting behavior, 191 genes encoding secreted proteins were up-regulated, of which 61 were SSPs (Additional file 7, sheet 2). From these SSPs, about half was transcribed >10-fold more during manipulation compared to the baseline expression and 28 were significantly down-regulated again after the event had taken place (Additional file 8, sheet 2). Of these, 16 did not receive a functional annotation. Our genome comparison analysis showed that 9 of these SSP genes were unique to the genome of *O. unilateralis* as well as one gene that contained an ankyrin repeat. Among those that were functionally annotated were 2 putative hydrophobic surface binding proteins (HsbA), 1 hydrophobin domain-containing protein, 1 cysteine-rich secretory antigen, 5 pathogenesis-related (CAP) protein, and a toxic ricin B lectin. Another SSP was GO annotated to have hydrolase activity and to be involved in a metabolic process. This gene was >100-fold up-regulated during manipulation and significantly down-regulated 6-fold again after. A homology search against the NCBI database suggested that this SSP is a putative kynurenine formamidase (Additional file 8, sheet 2). This hydrolase is involved in the kynurenine pathway of tryptophan metabolism [55], which produces nicotinamide adenine dinucleotide (NAD). NAD is involved in redox reactions by being an oxidizing agent. A putative up-regulation of its production through a high expression of kynurenine formamidase could therefore be involved in the overall up-regulation of oxidation-reduction processes seen during manipulation (see section below). Quinolinic acid and kynurenic acid are also formed through the kynurenine pathway. Their production could therefore also be increased through the high expression of this putative kynurenine formamidase. The formation of an excessive amount of quinolinic acid through the kynurenine pathway and failing to maintain physiological concentrations of kynurenic acid have been reported to have neuropathological effects [56–58]. Moreover, disturbance of the balance between the various pathways of tryptophan metabolism, such as the suggestive kynurenin pathway up-regulation in our data, could possibly result in serotonin depletion. In ants, serotonin depletion diminishes the likelihood that workers respond to the foraging trails, and when they do so, they walk shorter distances [59]. Indeed, field observations reported that ants infected by *O. unilateralis s.l.* do not walk on the trails and stay within the nest vicinity [60]. Taken together, the up-regulation during manipulation of this secreted hydrolase (Table 2 and Fig. 4), of which its homolog is involved in pathways that can cause neurological disorders, suggests another candidate possibly involved in establishing manipulated behavior in *O. unilateralis s.l* –infected ants.

Another candidate involved in manipulation is an SSP annotated to have a Lipocalin-2 domain (Table 2 and Fig. 4). A homology search suggested that this gene encodes for a D-like apolipoprotein (ApoD), which is a close ortholog to invertebrate Lazarillo-related proteins [61]. This SSP was not expressed in the fungal control culture, highly expressed during manipulation of ant behavior and 17-fold down-regulated again after manipulation (Additional file 8, sheet 2). Lipocalins are extracellular proteins found in bacteria, plants, vertebrates and invertebrates [62], including the order Hymenoptera in which the ants reside [61]. Homologs have also been reported from two other fungal genomes [63]. Our genome comparison analysis found homologs in *M. oryzae* (confirming [63]), *C. bassiana* and *C. militaris*. Lipocalins can be functionally diverse with roles ranging from the storage and transport of chemically sensitive or insoluble metabolic products [64], to participating in olfaction and chemical communication [65, 66], and regulation of (neuronal) stress and immune responses [67]. This makes lipocalin proteins interesting candidates in drug discovery. *O. unilateralis* could thus potentially secrete lipocalins as a way to regulate stress responses during manipulation of behavior, deliver secondary metabolites to the brain tissue, or alter chemical communication. Future functional assays are however needed to determine this.

Among the rest of the secreted proteins that were up-regulated during manipulated biting behavior and down-regulated again after manipulation, were 9 putative proteases: 6 serine proteases (1 tripeptidyl-peptidase and 5 subtilisins), 2 aspartyl proteases, and 1 metallocarboxypeptidase (Additional file 8, sheet 2). Pathogenic fungi commonly secrete these protease families. Though the function of fungal secreted proteases and their importance in infections may vary across species, these enzymes are largely considered important virulence factors [68]. Another putatively secreted protein that was up-regulated >1,500-fold during manipulation (Additional file 8, sheet 2) was annotated as a sphingomyelin phosphodiesterase. In fact, KEGG annotation indicated that this gene is a homolog of acid sphingomyelinase (ASM) SMPD1, an important enzyme in sphingolipid metabolism. Biological membranes consist of sphingolipids, together with cholesterol and phospholipids. As such, sphingolipids are involved in all types of cell regulation through cell recognition and cell signaling [69]. Neuronal cells are especially affected by defects in sphingolipid metabolism with defects in ASM specifically resulting in the neurological disorder Niemann-Pick syndrome [70]. This suggests that *O. unilateralis s.l.* could change neuron cell regulation through the secretion of a putative ASM in large amounts during manipulation, which is in line with the reported enrichment of the metabolite sphingosine in *ex vivo O. unilateralis s.l.* - *C. castaneus* ant brain interactions [12]. Moreover, the muscular atrophy accompanying manipulation and vegetation biting has been hypothesized to be the result of altered nerve motor neuron innervation [10]. Sphingomyelinase has, however, also been found to be an important factor for *Bacillus cereus* pathogenicity through the induction of host epithelial cell death in concert with the secretion of enterotoxins [71]. The up-regulation of secreted ASM could therefore also be complementing enterotoxin secretion, which suggests that this gene may be a virulence factor contributing to pathogenesis. The two effects manipulation and cell death may indeed be related as the biting behavior, which is the hallmark of *O. unilateralis s.l.* pathogenicity, does involve extensive atrophy [10].

Another secreted enzyme whose gene is being up-regulated during manipulation (>110-fold) and significantly down-regulated after (>5-fold), is a putative protein-tyrosine phosphatase (PTP*)* (Additional file 8, sheet 2). In fact, 2 more genes putatively encoding for this enzyme are significantly higher expressed inside the ant’s head compared to growth in culture (Additional file 7, sheet 2). For two baculoviruses, *Bombyx mori* nucleopolyhedrovirus and *Autographa californica* nucleopolyhedrovirus, the gene encoding for PTP was found to manipulate behavior of their respective insect hosts (*B. mori* and *Spodoptera exigua*). In these cases PTP induced enhanced locomotion activity (ELA) [5, 11]. ELA induced by baculoviruses occurs late in the infection during which infected caterpillars move to the upper plant foliage where they die. Similarly, we find *Ophiocordyceps*-infected ants biting at elevated positions. This suggests that *O. unilateralis s.l.* up-regulates putative secreted PTPs possibly causing enhanced locomotion behavior in ant hosts to ensure manipulated biting will take place outside of the nest and at an elevated position (Table 2 and Fig. 4).

### Differentially expressed *O. unilateralis s.l.* genes involved in oxidation-reduction processes

Among the genes that were significantly up-regulated during manipulated biting behavior, and down-regulated after manipulation had taken place, those involved in oxidation-reduction processes were significantly enriched (Additional file 6: Tables S1, S6 and S7). This annotation is rather general since many essential cellular processes require the oxidation and/or reduction of molecules involved. Interference in the balance between oxidants and reductants leads to oxidative- or reductive-stress conditions, which can result in inflammation, carcinogenesis, degenerative - and other diseases (reviewed in [72]). Moreover, parasite redox biology is unfolding to be an important aspect of parasite-host interactions. These recent studies have shown that host immune responses include oxidative attacks and the production of antioxidant enzymes by the parasite is considered vital for virulence, disease progression and outcome [73, 74]. During manipulation, 134 genes that were GO annotated to encode for proteins with an oxidation-reduction function were up-regulated (Additional file 7, sheet 2). After manipulated biting, 65 were significantly down-regulated again (Additional file 8, sheet 2). From this subgroup of differentially expressed genes, 15 were annotated to encode the cytochrome P450 proteins (CYPs) discussed above. These monooxygenases contribute to processes such as the biosynthesis of secondary metabolites, the assimilation of carbon sources, carcinogenesis, detoxification and degradation of xenobiotics [37, 75]. Of these CYPs, two were involved in secondary metabolism clusters through catalyzing the modification of intermediate products as “decorating” enzymes (Additional file 8, sheet 2). Both were actively transcribed during manipulated biting while transcription was basically absent during baseline expression and after manipulation had taken place. Another differentially expressed secretion signal-containing CYP was predicted to be involved in arachidonic acid metabolism working as a thromboxane-A synthase (CYP5A ortholog). Arachidonic acid is released from cell membranes by inflammatory stimuli and oxidized into compounds such as thromboxanes. These have been implicated as critical mediators in inflammatory diseases in vertebrates [76]. However, little or nothing is known about thromboxanes in insects at present. This suggests again that the fungal parasite might be regulating inflammation levels inside the ant’s head at the time of manipulated biting (Table 2 and Fig. 4). One CYP was predicted to be involved in phenylalanine metabolism and xenobiotics biodegradation working as a phenylacetate 2-hydroxylase. A fifth CYP with a predicted metabolism function was indicated as a CYP3A ortholog involved in steroid hormone biosynthesis, linoleic acid metabolism and retinol metabolism. This CYP was also KEGG annotated to have carcinogenic activities (Additional file 8, sheet 2). CYP3A members have largely been studied because of their dramatic accumulation induced by a diverse array of xenobiotics [77], which suggests that its up-regulation in *O. unilateralis s.l.* is due to the interactions with a live host and the stressors involved in such a process.

In addition to these CYPs, four other oxidation-reduction related secondary metabolite cluster genes were found to be up-regulated only during manipulated biting. One of these was annotated to encode for an NRPS-like enzyme and therefore suggested to be involved in the production of bioactive non-ribosomal peptides. The other three were “decorating” dehydrogenases predicted to be involved in i) steroid biosynthetic processes, ii) metabolism of bioactive terpenoids and polyketides that are widely used as therapeutic drugs and pharmaceutical agents [78], and iii) the biosynthesis of streptomycin, a compound that can have both antibiotic and toxic effects (Additional file 8, sheet 2). This KEGG ortholog for streptomycin biosynthesis is also homologous to a CYP within the Fusarin C cluster. Fusarin C is a mycotoxin with carcinogenic effects produced by several plant pathogenic *Fusarium* species [79] as well as insect pathogen *Metarhizium* [80]. This suggests that this up-regulated oxidoreductase might be involved in the biosynthesis of a fungal secondary metabolite with similar pathogenic effects for the host.

Of the secreted oxidoreductase activity containing proteins that were found to be up-regulated during manipulated biting behavior, four were annotated to function as tyrosinases (Additional file 7, sheet 2). Of those, two were significantly down-regulated again after manipulation had taken place (Additional file 8, sheet 2). Tyrosinases are type-3 copper proteins involved in melanin synthesis from tyrosine. They catalyze the hydroxylation of monophenols into diphenols and facilitate the oxidation of those into reactive quinones. As such, tyrosinases are especially responsible for the first steps resulting in the formation of dopaquinone [81] but have also been shown to play a role in other parts of dopamine synthesis and metabolism in the brain [82]. In fungi, tyrosinase is mainly associated with melanin production (i.e., browning and pigmentation). The functions of these melanins range from mechanisms of defense and resistance to environmental stresses [83] to virulence mechanisms [84]. Moreover, in insects, melanin plays a major role in the innate immune system by damaging and encasing invading microbes [85]. However, when dissecting *O. unilateralis s.l.* infected ants, no browning of the mycelium is observed [18] while browning of both the mycelium and the medium is seen in *Ophiocordyceps* cultures grown in culture [12]. The expression of tyrosinase was 2–8.7-fold higher during manipulated biting behavior when compared to expression during growth in culture. This suggests that tyrosinase might be up-regulated not merely to produce melanin. Research indeed shows that the quinones generated from dopamine and DOPA by tyrosinase are cytotoxic both in and beside dopaminergic neurons through the interaction with a variety of bioactive molecules [86]. Moreover, quinone formation has been related to pathogenesis in Parkinson’s disease [82], mitochondrial dysfunction in the brain [87], inflammation leading to degeneration of dopamine neurons as seen in methamphetamine neurotoxicity [88, 89] and proteasome impairment [90]. The specific up-regulation of fungal tyrosinases during the behavioral manipulation of the ant host might therefore exert more than a mere virulence or environmental stress response function (Table 2 and Fig. 4).

### Differentially expressed pathogenicity-related *O. unilateralis s.l.* genes

Since this mixed transcriptomics study aims to reveal the gene expression at a particular time point during a parasite-host interaction, an up-regulation of pathogenicity-related genes in the parasite is to be expected. We indeed found putative pathogenesis-related genes from different classes, some of which have already been discussed above. During manipulated biting behavior we also found 2 ricin-type lectins, 1 fucose specific lectin and 1 lectin-like flocculation protein to be significantly higher expressed compared to both expression in the baseline culture and heads of ants after manipulation had taken place (Additional file 8, sheet 2). Lectins bind carbohydrates and mediate recognition in parasite-host interactions. They are highly sought after because of their great therapeutic and biotechnological potential. The roles of many fungal lectins remain elusive, however functions such as host recognition and yeast flocculation have been proposed [91]. Furthermore, a ricin-type lectin from *Rhizoctonia solani* was found to induce significant toxicity in insect pests [92]. Another protein with interesting biotechnological potential that was found to be up-regulated during manipulation was a putative aegerolysin. Fungal aegerolysins are said to have anti-tumoral, anti-proliferative, and anti-bacterial activities as well as being involved in the early stages of fructification [93]. A homology search for this gene revealed that this *O. unilateralis s.l.* aegerolysin shares 63 % similarity with Asp-hemolysin of *A. fumigatus*, which is a lethal and cardiotoxic component of this pathogenic fungus [94]. This suggests that *O. unilateralis s.l.* up-regulates an array of possible virulence factors when growing inside its ant host. These are essential to understand the parasite-host interactions taking place, as well as of potential biotechnological interest for the fields of medical drug discovery and biological pest control.

The genome comparison (discussed above) showed that *O. unilateralis s.l.* has far more genes encoding secreted putative bacterial-like enterotoxins compared to other fungi studied so far. Of the 34 putative enterotoxins in the *O. unilateralis s.l.* genome, 12 were not transcriptionally active under the conditions used in this study. Of the transcriptionally active ones, only two were constitutively expressed across the conditions tested leaving a total of 21 differentially expressed enterotoxins. During manipulated biting behavior, six enterotoxins were up-regulated compared to expression during growth in culture. Two of these were significantly down-regulated again after manipulation had taken place. Nine were down-regulated during manipulated biting behavior, of which three were significantly up-regulated again afterwards. This highly dynamic expression of the majority of enterotoxins indicates that their expression is tailored towards the environmental conditions that *O. unilateralis s.l.* encounters and different life cycle stages.

### Pathology-related changes in *C. castaneus*: immune reaction and apoptosis

So far we have discussed changes in the gene expression of the fungal parasite during and after manipulated biting behavior (summarized in Fig. 4). The mixed transcriptomics approach, allows us to also investigate gene expression of the ant host at these time points (also summarized in Fig. 4). Because this is a host-parasite system it might be expected that immune-related gene expression in the host is altered because of parasitism. Previous studies on gene expression in ants have shown an up-regulation of immune-related genes upon infection by bacteria [95] and the generalist fungus *Metarhizium brunneum* [35, 96]. These studies sampled the ants in the early stages of the infection, 24 and 48 h after the entry of the pathogen respectively. In our study we sampled more than two weeks after the entry of the parasite as this amount of time is needed to establish manipulation [12]. We found that many of these immune-related genes were down-regulated in ant heads at time of manipulated biting compared to heads of healthy ants. This included genes involved in pathogen recognition (*e.g.* peptidoglycan recognition protein), signaling (*e.g.* cytochrome p450 6 k1) and immune response effectors, such as prophenoloxidase and antimicrobial peptides (AMPs) (defensin 1, defensin 2 and a homolog of dynamin) (Additional file 10, sheets 4 and 5). Other genes important in insect immune and stress responses, such as the homologs of *malvolio* and *punch* [97], were constitutively expressed across the controls and infected samples. Moreover, our enrichment analysis revealed that among the 391 down-regulated genes in the head of the live host during manipulated biting, those related to oxidoreductase activity were overrepresented (Additional file 7: Table S6). This suggests that *O. unilateralis s.l.* might be able to suppress host immune responses in the head during the event of manipulated biting behavior. Similarly, stress-related genes were also differentially expressed in infected ants. Again, the *Ophiocordyceps* infection resulted in down-regulation of most common stress-related genes in insects, such as the homolog of the insect lipocalin *lazarillo* [98]. However, at the same time, a secreted fungal lipocalin was up-regulated (discussed above) implying that the parasite might be regulating the host’s neuronal stress response.

Although parasites suppressing the expression of ants’ immune responses have not been previously documented, it has been shown for other hymenopterans (ants belong to the order Hymenoptera). The ectoparasitic mite *Varroa destructor* suppresses the innate immune system of honeybees (*Apis melifera*) by reducing the expression of AMPs and immunity-related enzymes [99]. The bumblebee *Bombus terrestris* has its immune response affected by the trypanosome gut parasite *Crithidia bombi* through the down-regulation of AMPs [100]. Furthermore, other trypanosome parasites that cause vector borne diseases, such as *Trypanosoma brucei* (sleeping sickness) and *Leishimania* (leishmaniasis), also repress immune reaction related expression in their insect vectors (tsetse and sandflies, respectively) [101, 102]. All three of these parasites are co-evolved specialists unlike the generalist pathogens used in [35, 96]. We suggest that *O. unilateralis s.l.,* like other specialist parasites, might suppress the immune response in heads of the ants it infects.

At the moment of manipulation, the fungal cells occupy the head of the ant host, causing severe atrophy and a reduction in organelles of muscle cells, but leaving the brain preserved [10]. However, 48 h post-mortem, brain tissue was virtually indistinguishable from the fungal cells [18]. In line with this previous evidence of tissue breakdown, we found the differential expression of a number of apoptosis-related genes during the manipulation, compared to the healthy controls. Overall, pro-apoptotic genes were found to be up-regulated during the manipulated biting event (Additional file 10, sheet 4). These included proteins annotated as feminization-1 (Fem1b) homolog b-like and specificity protein-3 transcription factor (Sp3). Fem1b plays a role in sex determination in *Caenorhabditis elegans* regulating cell elimination through apoptosis [103, 104]. In humans, the up-regulation of its homologue (FEM-1) induces apoptosis via the caspase pathway [105, 106]. The zinc finger transcription factor Sp3 is important for the regulation of genes implicated in cell cycles and programed cell death [107]. Its overexpression results in apoptosis of cancer cells [107–109] and cortical neuron cells in culture [110]. Sp3 was also found to be accumulated in the post-mortem brain tissue of patients with Alzheimer disease, and has been suggested to play a role in the neurodegenerative progression characteristic of Alzheimer, which in humans results in cognitive loss [110]. We suggest that preserving the brain during colonization of the head may be necessary for *O. unilateralis s.l.* to successfully manipulate behavior. However, after the ant host is successfully attached to a substrate (the time point at which we collected the manipulated ants for RNA-Seq analysis), the fungus appears to either induce neural apoptosis or release hydrolytic enzymes that cause host tissue degradation to be able to complete its development.

### Possible ant host targets of behavioral manipulation

Prior to manipulated biting, infected ant hosts display a stereotypical walking pattern, followed by constant climbing and falling from the vegetation due to whole body convulsions [10]. This suggests neuronal activity is affected. In line with this, in the head of the ant sampled at 10:00 h during the manipulated biting, we found the differential expression of putative biogenic amine precursors and receptors. Biogenic amines are known to influence behavior of both vertebrates [111, 112] and invertebrates [113–117], including ants [118–120]. We for instance found the up-regulation of a putative beta-3R-like octopamine receptor gene and a tyrosine 3-monooxygenase gene (Additional file 10, sheet 4). Tyrosine 3-monooxygenase is the rate-limiting enzyme for the biosynthesis of dopamine [121]. Although dopamine is a precursor for the melanin pathway in the immune response of insects (ants included: [35, 95]), it was demonstrated in *Drosophila melanogaster* that dopamine also functions as a neurotransmitter in the fly nervous system. Furthermore, the expression of tyrosine 3-monooxygenase appears to oscillate in a circadian manner in *D. melanogaster*, regulating motor neuron activity and neuromuscular functions through the biosynthesis of dopamine [122]. This results in the circadian regulation of locomotion behavior [121]. The up-regulation of this enzyme could therefore be involved in establishing the aberrant walking behavior that is observed in *O. unilateralis s.l.* infected ants [10]. Moreover, increases in the dopamine levels in *Formica polyctena* ants (by direct injection) were found to stimulate the opening of mandibles and biting [120]; behaviors that were both observed in manipulated ants moments before the biting event. This could indicate that the up-regulation of tyrosine 3-monooxygenase plays a role in the behavioral changes observed in *O. unilateralis s.l.*-infected ants.

In addition to walking aberrantly, manipulated ants do not follow trails like their healthy nest mates [10, 60]. One possible explanation is that the manipulated ants receive limited stimuli from the environment. We indeed found genes putatively encoding for receptors and binding proteins involved in odorant and gustatory perception (*e.g. OR-1* and *ORCO*) were down-regulated during manipulated biting behavior relative to gene expression in healthy ant heads (Additional file 10, sheet 5). Making the host latch on in the optimal microenvironment for fungal growth and spore dispersion is crucial to the life cycle of this parasite [18, 60]. The erratic walking has been observed in nature to last at least three hours [10]. Suppressing the sensorial perception could therefore be a strategy to keep the ant engaged in this process. However, mosquitoes infected with the generalist fungal pathogen *C. bassiana*, which does not manipulate host behavior as a way to achieve transmission, were found to be less responsive to feeding stimuli, as well [123]. The lack of response to stimuli could therefore also be a general pathology-related host reaction or an ancient characteristic of entomopathogenic fungi in general.

Another characteristic feature of the manipulation of *C. castaneus* behavior by *O. unilateralis s.l.* is the switch to diurnality. Uninfected *C. castaneus* are primarily nocturnal ([124] and our extensive field observations). Moreover, the manipulated biting behavior and subsequent death appears to be synchronized to a certain time of day, both in the laboratory and in nature (this study and [10], respectively). This has led to the hypothesis that the fungus might affect the circadian clock of the host [12]. The circadian clock is a temporal program that anticipates the highly predictable daily changes of the environment. The molecular mechanism of the biological clock is conserved across different groups of organisms [125], exemplified by positive and negative transcriptional feedback loops on one hand, and the redox state of peroxiredoxin on the other hand [126]. In insects, two of these clock genes are *period* and *cycle.* Fire ant (*Solenopsis invicta*) *period* and *cycle* oscillate in anti-phase in their transcript levels [127]. In manipulated ants (10:00 h), *period* and *cycle* homologs were expressed with a 2.3 and 2.1-fold increase, respectively, compared to uninfected controls sampled at 10:00 h (Additional file 10, sheet 5). This may affect the expression of clock-controlled genes regulating certain behaviors such as the above-mentioned oscillating tyrosine 3-monooxygenase in *Drosophila* [121, 122]. The circadian clock in some organisms regulates cell cycle control, proliferation and apoptosis [128, 129]. For instance, the over- and under-expression of homologous clock genes *PER1* [130] and *BMAL1* [131] in human cells correlates with the rapid proliferation of aggressive tumor cells and to tumor cell control through apoptosis [132, 133], respectively. This is consistent with our apoptosis-related findings discussed above and previously reported morphological data [10].

*O. unilateralis s.l.-*infected ants display increased and constant activity before the manipulated biting takes place [10]. This is suggestive of the increased locomotor activity in caterpillars infected with baculovirus [5]. In caterpillars, this behavior is related to the expression of *ptp*. We also found a significant up-regulation of PTP-encoding genes during manipulation, both in the ant (Additional file 10, sheet 4), as well as in the fungal transcriptome (Additional files 7 and 11, sheet 2). The observed enhanced locomotion in behaviorally manipulated ants could be a result of this up-regulated *ptp* expression. Ultimately, the manipulation of ant host behavior results in biting the vegetation at an elevated position, which fixes the ant to a substrate and is crucial for fungal development and transmission [18, 60]. The deep penetration of the mandibles into the plant tissue and the atrophy of the mandibular muscles cause the “lock-jaw”, ensuring the lasting attachment of the ant even once it has been killed by the fungus. The muscle atrophy is characterized by muscle fiber detachment from the head capsule, with broken z-lines (anchors for muscle contraction) and less dense sarcoplasmic reticula and mitochondria [10]. In our transcriptomics study, we found that two genes encoding for collagen, homologues of *COL4A1* and *COL4A2*, the major protein of the muscle fibers [134], and a muscle LIM protein Mlp84B homolog, which is a component of the Z-lines and important for maintaining the muscles integrity [135], were down-regulated during manipulated biting (Additional file 10, sheet 5). These suggest a mechanism by which the fungus establishes the host muscle atrophy that is important for the lock-jaw and, consequently, long term fixation of the dead ant to a plant substrate. These genes, together with the others mentioned above, which are involved in host pathways that are possible hallmarks for *O. unilateralis s.l.* to establish the different aspects of behavioral manipulation, are summarized in Table 3 and Fig. 4.

**Table 3:** Candidate *C. castaneus* targets of behavioral manipulation

Protein ID	Functional annotation	Fold change during manipulation
Up-regulated		
Camfl1|CFLO16350-PA	Putative protein-tyrosine phosphatase, involved in ELA behavior induction in Lepidoptera by baculoviruses	8.39
Camfl1|CFLO14092-PA	Octopamine receptor Beta3r homolog, putative biogenic amine receptor involved in the G-protein pathway	6.26
Camfl1|CFLO22298-PA	Putative specificity protein-3 transcription factor, pro-apoptotic function, also found in Alzheimer brains	5.74
Camfl1|CFLO15698-PA	Feminization-1 homolog, pro-apoptotic function, protein binding function	3.14
Camfl1|CFLO22839-PA	Putative protein-tyrosine phosphatase, involved in ELA behavior induction in Lepidoptera by baculoviruses	3.82
Camfl1|CFLO22748-PA	Putative tyrosine 3-monooxygenase, circadian, dopamine biosynthesis and regulation of locomotion behavior	2.96
Camfl1|CFLO17010-PA	Putative protein-tyrosine phosphatase, involved in ELA behavior induction in Lepidoptera by baculoviruses	2.88
Camfl1|CFLO19262-PA	Putative protein-tyrosine phosphatase, involved in ELA behavior induction in Lepidoptera by baculoviruses	2.44
Camfl1|CFLO16807-PA	Period homolog, circadian clock gene, negative feedback loop	2.25
Camfl1|CFLO21782-PA	Putative protein-tyrosine phosphatase, involved in ELA behavior induction in Lepidoptera by baculoviruses	2.18
Camfl1|CFLO22839-PA	Cycle homolog, circadian clock positive feedback loop	2.11
Down-regulated		
Camfl1|CFLO13023-PA	Putative prophenoloxidase, oxidoreductase activity	24.03
Camfl1|CFLO15551-PA	Putative general odorant binding protein involved in chemosignaling	10.00
Camfl1|CFLO13952-PA	Dynamin homolog, putative antimicrobial peptide, GTPase activity involved in immune response	8.08
Camfl1|CFLO13270-PA	Putative pheromone-binding protein involved in chemosignaling	7.74
Camfl1|CFLO16107-PA	Defensin1 homolog, putative antimicrobial peptide, immune response	5.35
Camfl1|CFLO17717-PA	Defensin2 homolog, putative antimicrobial peptide, immune response	4.66
Camfl1|CFLO22173-PA	Odorant receptor or-1 homolog involved in chemosignaling	4.37
Camfl1|CFLO15004-PA	Putative odorant receptor co-receptor involved in chemosignaling	4.03
Camfl1|CFLO16950-PA	Cytochrome p450 6k1 homolog, oxidoreductase activity, ion binding, involved in immune response	3.63
Camfl1|CFLO15757-PA	Putative collagen alpha-2 (IV) chain, muscle related	3.19
Camfl1|CFLO17070-PA	Putative odorant binding protein involved in chemosignaling	2.73
Camfl1|CFLO21048-PA	Muscle LIM protein Mlp84B homolog	2.30
Camfl1|CFLO23127-PA	Putative peptidoglycan recognition protein involved in catabolic processes and pathogen recognition	2.29
Camfl1|CFLO21233-PA	Putative lipocalin involved in stress responses	2.23
Camfl1|CFLO15749-PA	Putative collagen alpha-1 (IV) chain, muscle related	2.12

## Conclusions

Given the novelty and precise nature of the behavioral changes observed, unraveling the genetic basis of fungal control of ant behavior is of great interest to the field studying parasitic manipulation of host behavior, as well as disciplines ranging from parasitology and animal behavior to parasite-host co-evolution and fungal genetics. Moreover, the long track record of fungi producing a wealth of industrially interesting bioactive compounds and the expectation that brain-manipulating species produce novel neuromodulators [41], make genetic studies into this system important from an applied perspective (both in medicine and insect pest control). Here, we used a novel *O. unilateralis s.l.* genome and the published *C. floridanus* genome as references to assess the gene expression in the heads of infected Carpenter ants during manipulated biting behavior.

Comparing the novel *O. unilateralis s.l.* genome to other currently available fungal genomes revealed that this manipulative fungus putatively secretes an array of novel compounds that might have interesting biological properties. Moreover, 80 % of the *O. unilateralis s.l.* genes that were uniquely up-regulated during manipulated biting behavior were found not to be conserved among other non-manipulating insect pathogens of the order Hypocreales. This indicates that, in order to establish manipulated biting, *O. unilateralis s.l.* employs a set of genes that is not present in the genomes of non-manipulating fungi. However, *O. unilateralis s.l.* seemingly has a mechanism to change behavior in common with a viral manipulator (i.e., *ptp* expression). Future comparisons with other closely related (*Ophiocordyceps*) and non-related manipulating parasites will therefore be interesting to investigate if the ability to manipulate host behavior has either convergently or divergently evolved.

Our study also shows that *O. unilateralis s.l.* has an abundance of genes encoding for bacterial-like enterotoxins, which are dynamically up- and down-regulated in the situations tested in this study. These compounds could negatively affect the production of chemosignaling molecules in insects [31, 32], which would impair communication in infected individuals. In agreement with this, infected ants are generally observed to be unresponsive to external stimuli [10, 60] and host genes putatively encoding for receptors and binding proteins involved in odorant and gustatory perception were found to be down-regulated. However, the dynamic expression of putative *O. unilateralis s.l.* enterotoxins could also be contributing to the up-regulated apoptotic features found in manipulated *C. castaneus* ants and the severe atrophy of muscle cells observed in histology studies [10].

Genes putatively involved in immune- and stress responses were found to be generally down-regulated in infected ant heads during the manipulated biting event. In fact, oxidoreductase activity-related genes were enriched in the subset of down-regulated ant genes. Oxidation-reduction reactions are vital mechanisms in parasite-host interactions for both parasite and host [73]. As such, in the fungal parasite, we indeed found genes involved in these processes being up-regulated during manipulation and down-regulated again after the host was presumably killed. While *O. unilateralis s.l.* ultimately kills its host, premature death will not lead to manipulation [12] thus impairing transmission and completion of the life cycle. As suggested for other insect-parasite interactions [100], suppression of host responses might therefore be crucial to create conditions in the host that favor parasite development. In addition to causing apoptosis and impairing chemosensory communication, *O. unilateralis s.l.* might therefore be regulating the host’s immune- and neuronal stress responses as a mechanism to establish manipulated behavior.

Other links between the fungal parasite and ant host transcriptomes during manipulated biting behavior are the up-regulation of genes encoding for enzymes involved in dopamine metabolism and protein-tyrosine phosphatases. Both pathways have reported effects on behavioral outputs such as locomotion and mandible movement, which are hallmarks of ant infections by *O. unilateralis s.l.* fungi. Moreover, homologs of clock genes *per* and *cycle* were up-regulated in manipulated ants, which would affect processes in the host, ranging from the cellular to the behavioral output level. In addition, we found the up-regulation of various fungal genes involved in different types of alkaloid metabolism that lead to compounds that can both work as agonists and antagonists of a range of receptors in the central and peripheral nervous system. Together with the up-regulation of a putatively secreted sphingomyelinase that changes neuron cell membranes, a possible shift in tryptophan metabolism from the serotonin pathway towards the kynurenine pathway and the production of NRPSs, PKSs and small secreted proteins with unknown functions, this represents an array of mechanisms and compounds through which the ant host brain could be manipulated.

In conclusion, we found various candidate manipulators and possible host pathways through which manipulated biting behavior as seen in *O. unilateralis s.l.*-infected Carpenter ants could be established. While some of these candidates could be essential to the process, we expect these to work in concert since the manipulated behavior in this system is complex and precise. Future functional gene expression studies are necessary to determine this. Furthermore, this study only assesses the ultimate time points in this parasite-host interaction: manipulated biting and killing of the host afterwards. This leaves the mechanisms underlying the reactions and more subtle behavioral changes leading up to this still unresolved. That said, our study demonstrates that experimental infections combined with behavioral studies to determine sampling points followed by mixed transcriptomics analyses will be a good approach to study the parasite-host interactions at these earlier stages as well.

## Methods

### Fungal strain and maintenance

*O. unilateralis s.l.* strain SC16a isolated from an infected *C. castaneus* worker ant collected in South Carolina, USA was selected for genome sequencing and RNA-Seq since infection and manipulation of host behavior by this fungus was successfully recapitulated in the laboratory [12]. Fungal cultures were maintained in Grace’s insect medium (Sigma) freshly supplemented with 10 % fetal bovine serum (PAA Laboratories Inc.). For both genome sequencing and RNA-Seq, fungal cultures were grown in 250 mL shaking flasks holding 50 mL supplemented insect medium shaken at 150 rpm at 28 °C. Cultures used for infections were transferred to potato dextrose agar prior to preparing the material for injection.

### Genome sequencing and assembly

For genome sequencing two different genomic libraries were prepared to avoid fragmentation bias. The first library was prepared with a Nextera kit (Illumina) according to manufacturer's instructions. For the second library 5 μg of genomic DNA were sheared in a Covaris M220 ultrasonicator with 50 μl screw caps to an approximate length of 800 bp. The fragmented DNA was used for library preparation with the NEBNext DNA Library Prep Master Mix Set for Illumina (New England Biolabs) according to manufacturer’s instructions. Both libraries were automatically size selected to a final length of 650–850 bp by separation on a 1.5 % agarose gel in a BluePippin electrophoresis unit (Sage Science). Sequencing was performed with a MiSeq sequencer (Illumina) yielding in total 13.4 Mio 2×300 bp paired-end sequences (1.4 Mio sequences for the Nextera library and 12.0 Mio sequences for the NEBNext library). Sequences were *de novo* assembled with CLC Genomics Workbench (Qiagen) using default parameters. 98 % of primary sequences assembled into 7,875 scaffolds (minimum length 300 bp, average coverage of 120-fold) with a total genome size of 26.05 Mb. This Whole Genome Shotgun project has been deposited at DDBJ/EMBL/GenBank under the accession LAZP00000000. The version described in this paper is version LAZP01000000. Repetitive sequences in the assembly were masked using RepeatMaker [136], RepBase library [137] and RepeatScout [138].

### Gene prediction

Genes were predicted for *O. unilateralis* with Augustus version 3.0.2 [139] using the supplied parameter set for *Fusarium graminearum*. To aid in gene prediction, intron hints were produced from the Tophat transcript alignments (described below) using the Augustus software package. Introns were subsequently filtered for having at least 20 reads mapping across it and (in the case of alternative splicing) spliced in at least 50 % of the cases. Completeness of the set of predicted proteins was analyzed using the CEGMA protein set [25] using a blastp E-value cutoff of 1e-5.

### Functional annotation

The predicted proteins of *O. unilateralis* and those of the previously published *C. floridanus* [23] were functionally annotated. In addition to these, we included 16 previously published fungal genomes for comparative purposes: *Ophiocordyceps sinensis* [140], *Tolypocladium inflatum* [27], *Cordyceps militaris* [141], *Cordyceps bassiana* (formerly *Beauveria bassiana*) [30], *Metarhizium robertsii* (formerly *Metarhizium anisopliae*) and *Metarhizium acridum* [142], *Claviceps purpurea* [143], *Fusarium oxysporum* [144], *Fusarium graminearum* [145], *Trichoderma reesei* [146], *Trichoderma virens* [147], *Magnaporthe oryzae (*formerly *Magnaporthe grisea*) [148], *Candida albicans* [149], *Aspergillus fumigatus* [150], *Neurospora crassa* [151], *Aspergillus nidulans* [152].

Conserved protein domains were predicted using PFAM version 27 [26] and were subsequently mapped to the corresponding gene ontology (GO) terms [153, 154]. Metabolic genes were predicted using KEGG on the KAAS server [155]. Proteases were predicted using the MEROPS database [156] with a blastp E-value cutoff of 1e-5. Secretion signals were predicted using Signalp 4.1 [157]. Transmembrane domains were predicted using TMHMM 2.0c [158]. Proteins with a secretion signal, no transmembrane domain (except in the first 40 amino acids) and a total length shorter than 300 amino acids were considered small secreted proteins. Genes and gene clusters involved in secondary metabolism were predicted using a pipeline based on the SMURF method [159]. SMURF parameter *d* (maximum intergenic distance in base pairs) was set at 3000 bp, and SMURF parameter *y* (the maximum number of non-secondary metabolism genes upstream or downstream of the backbone gene) was set at 6.

### Comparative genomic analysis

Clusters of orthologous genes among the fungal genomes were identified using OrthoMCL version 2.0.9 [160] using an inflation factor of 1.5. To estimate the phylogeny of these organisms, 168 orthologous groups of genes having exactly one gene in each organism were identified. The sequences of each species were concatenated, aligned using MAFFT version 7.123b [161] and well-aligned regions were extracted using Gblocks 0.91b [162]. This resulted in 73994 amino acid positions. The parallelized version of RAxML version 8.1.16 [163] with the PROTGAMMAWAG model with 100 rapid bootstrap partitions was used to reconstruct a species tree. The tree was visualized using Dendroscope version 3.2.10 [164].

Proteins that contained a ‘heat-labile enterotoxin alpha chain’ domain (PFAM domain PF01375) were identified as enterotoxin. The domains were aligned using MAFFT version 7.123b [161]. Next, a phylogenetic tree was reconstructed with FastTree 2.0 using default parameters [165]. The tree was visualized using Dendroscope version 3.2.10 [164] and rooted on the monophyletic group containing the sequences from *M. oryzae*.

### Ant host infections and behavioral observations

Worker ants of the species *C. castaneus* obtained in SC, USA (private property in Due West (GPS 34.332110, −82.387131)) were used for infections followed by RNA-Seq. In nature, this species is also infected by the fungal parasite used in this study. Ants were infected or sham treated by ways of injecting as previously described [12]. After the infection procedure, the colony was housed in a 513 cm^2^ cage with a darkened, two-dimensional 140 cm^2^ wooden nest and sand at the bottom. Additionally, the cage held a climbing and biting platform as well as water and 10 % sugar water *ad libitum*. Ants were kept under strict light (LD1212) and temperature cycles with light between 06:00 h and 18:00 h and a 10 °C increased temperature between 10:00 h and 16:00 h [12]. Ants were randomly assigned to a treatment group and color-coded (Edding): 50 untreated (no color), 35 infected (purple thorax - injected with the fungus) and 15 sham treated (green thorax - injected with medium). Daily observations were made at 9:00 h, 13:00 h and 17:00 h up to 30 days post infections to record survival status, location within the cage and the presence of manipulated climbing and biting behavior of individuals from each of the treatment groups.

### Preparation of controls and infected samples

To reduce variation within sample type, we used heads of ants that were collected on the same day. The maximum number found manipulated on the same day was three. Therefore, each of the sample types was prepared in biological triplicates. *O. unilateralis s.l.* controls were generated from saturated shaking cultures that were grown for 14 days. These cultures were harvested over a sterile Buchner funnel with Whatmann paper using suction. Dried mycelium was subsequently snap frozen in liquid nitrogen and stored at −80 °C. Healthy *C. castaneus* controls were collected after keeping them under the same strict 24 h cycles as the infected ants for 30 days. The day prior to sampling, 6 untreated ants were divided over 2 small cages placed next to the cage used for the infection experiment to be able to snap freeze them in liquid nitrogen without inducing stress due to sampling. At 10:00 h 3 ants were harvested by quickly inverting one small cage into liquid nitrogen. These ants function as controls for infected ants displaying manipulated biting behavior. At 14:00 h the 3 additional ants were harvested to function as controls for ants that died after the manipulated biting event. Infected ants displaying the manipulated biting behavior were harvested at 10:00 h by placing them, together with the twig they were biting, into liquid nitrogen. Similarly, infected ants that appeared lifeless after biting were harvested at 14:00 h. Manipulated ants, not reacting to any environmental stimuli anymore, would not be disturbed by the sampling procedure. After snap freezing, twigs were removed and harvested ants were stored at −80 °C prior to RNA extraction.

### RNA extraction, mRNA-Seq library construction and sequencing

RNA was extracted from 1 cm^2^ pieces of the *O. unilateralis* control cultures, and the heads from both the infected and the healthy control ants. These samples were transferred to liquid nitrogen cooled 2 mL Eppendorf tubes (Greiner) with two metal beads (4.76 mm in diameter). Cells were mechanically disrupted inside these frozen 2 mL Eppendorf using a TissueLyser II (Qiagen) and a chilled adapter set (Qiagen) at 24 freq/s for 60 s. RNA was subsequently extracted according to a previously reported protocol [166]. The quality and quantity of the samples were checked using a Bioanalyzer (Agilent Technologies) and a NanoDrop (Thermo Scientific). Subsequently mRNA-Seq libraries were constructed using a TruSeq Stranded mRNA Sample Prep Kit (Illumina). Samples were sequenced on an Illumina HiSeq 2500 using 100 × 100 paired-end sequencing in Rapid Run mode. The RNA-Seq expression dataset is available at the NCBI’s Gene Expression Omnibus under the accession code GSE68176.

### Mixed transcriptome analysis

Both single species RNA reads (as controls for both parasite and host) and mixed RNA reads (in case of parasite-host interactions) were used in this study. Sequence tags were mapped to the respective parasite and host genome sequences using the programs TopHat version 2.0.11 [167] and Bowtie version 2.2.2 [168]. Default settings were used, with the exception of intron length, which was set as a minimum of 5 nucleotides and a maximum of 1000 nucleotides in the case of *O. unilateralis*. Moreover, the microexon-search option was enabled. The program Cuffdiff version 2.2.1, which is part of Cufflinks [169], was used to identify reads overlapping with the predicted genes and to identify differentially expressed genes. Default settings were used. The expression levels of each predicted gene were normalized to Fragments Per Kilobase of exon model per Million fragments (FPKM). The bias correction method was used while running Cuffdiff [170]. For differential expression we also applied a cutoff of at least a 2-fold change in expression value, as well as at least one condition with an expression value of at least 4 FPKM. Over- and under-representation of functional annotation terms in sets of differentially regulated genes were calculated using the Fisher Exact test. The Benjamini-Hochberg correction was used to correct for multiple testing. As a cutoff for significance we used a corrected *p*-value of 0.05. To visualize gene expression a heat map was generated using thw ‘heatmap.2’ module of the statistics software package R 3.2.0. First, the expression values were log2-transformed after increasing the values with 1 in order to avoid negative log2 values. Next, the genes were clustered using the euclidean distance and average linkage methods. The values were scaled for each gene, resulting in a z-score.

## Additional files

Additional file 1:
**KEGG annotations of genes found in the** ***O. unilateralis s.l.*** **genome.** Pie charts representing the first and second level KEGG annotations that were found in the *O. unilateralis s.l.* genome. First level annotations are indicated in the multi-color pie chart in the center. Second level annotations are indicated in the surrounding pie charts, displayed in different shades of the color of their first level annotation. (PDF 377 kb) Additional file 2:
**Phylogenetic relationship between genes annotated to have an enterotoxin_a PFAM domain.** Phylogenetic tree of all genes in this study that contain one or more enterotoxin_a PFAM domains. The tree was rooted on *M. oryzae* enterotoxins. (PDF 190 kb) Additional file 3:
**Mixed and single transcriptome sequencing and genome mapping statistics.** Table summarizing the sequencing statistics for each transcriptomics sample used in this study as well as the percentage of reads that mapped to the respective parasite and host reference genomes. For all samples, 100 % of the clusters passed filtering and 100 % of the reads in the sample matched the given index. (PDF 90 kb) Additional file 4:
**Differential gene expression in parasite and host during and after manipulated biting behavior.** Bar charts displaying the number of genes that are up- and down- regulated during the parasite-host interaction. (A) The fungal parasite *O. unilateralis s.l.* gene expression during and after manipulated biting behavior are both compared to a baseline gene expression displayed by the fungus in insect cell culture media and to each other. (B) The ant host *C. castaneus* gene expression during and after manipulated biting behavior are both compared to the gene expression in healthy hosts sampled at the same time of day and to each other. The amount of genes that are differentially expressed during manipulated biting compared to both the situation before (controls) and after the event are indicated in green (Uniquely Up, − Down). Genes were considered differentially expressed when expression was at least 4 FPKM for one of the sample types and significantly (Q < 0.05) changed > =2-fold. (PDF 157 kb) Additional file 5:
**Mapped fungal and insect reads in mixed transcriptome samples compared to single transcriptome controls.** Bar charts displaying the percentage of RNA-Seq reads in both single and mixed transcriptome samples mapped to either the parasitic *O. unilateralis s.l.* genome (A) or the *C. floridanus* genome (B). The single transcriptome samples (Fungal Culture and Healthy Ant heads; black bars) display the read mapping when all of the biological material is derived from one organism. Relative comparison to this percentage reads mapped suggests that during manipulated biting behavior about half of the biological material is ant tissue, while the other half comprises of fungal cells. Right after the manipulated biting event the amount of fungal cells inside the head appears to have increased, while the amount of host tissue has decreased even further. (PDF 131 kb) Additional file 6:
**Enrichment analysis on differentially expressed** ***O. unilateralis s.l.*** **genes.** Tables summarizing the overrepresentation of annotation terms for *O. unilateralis s.l.* genes found to be up- and down-regulated during manipulated biting behavior in comparison to baseline gene expression in fungal cultures (**Tables S1 and S2**, respectively), after manipulated biting behavior in comparison to baseline gene expression (**Tables S3 and S4**), during manipulated biting behavior versus death after manipulated biting behavior (**Tables S5 and S6**), and genes that are uniquely up- or down-regulated during manipulated biting behavior (**Tables S7 and S8**). (PDF 263 kb) Additional file 7:
**Differentially expressed** ***O. unilateralis s.l.*** **genes.** Tables displaying all *O. unilateralis s.l.* genes, including their expression levels and annotation, that were found to be differentially expressed during manipulated biting behavior in comparison to baseline gene expression in fungal cultures (sheets 2 and 3), after manipulated biting behavior in comparison to baseline gene expression (sheets 4 and 5), and during manipulated biting behavior versus death after manipulated biting behavior (sheets 6 and 7). (XLSX 891 kb) Additional file 8:
***O. unilateralis s.l.*** **genes uniquely up- or down regulated during manipulated biting behavior.** Tables displaying all *O. unilateralis s.l.* genes, including their expression levels and annotation that were found to be up-regulated during manipulated biting behavior followed by being down-regulated again after the biting event had taken place (sheet 2), and that were found to be down-regulated during manipulated biting behavior followed by being up-regulated again after the biting event had taken place (sheet 3). (XLSX 232 kb) Additional file 9:
**An expression heat map of differentially expressed** ***O. unilateralis s.l.*** **genes that contain a putative secretion signal.** Of 891 genes encoding a protein with a putative secretion signal, 429 (48 %) were differentially expressed. Green indicates a relatively high expression of that gene compared to other conditions, whereas red indicates relatively low expression. Genes are clustered based on similarity of expression profile. The large differences in expression between the samples are clearly visible. Genes annotated to be small secreted proteins (SSPs) are indicated with a line in between the dendrogram and the heat map. (PDF 538 kb) Additional file 10:
**Differentially expressed** ***C. castaneus*** **genes.** Tables displaying all *C. castaneus* genes, including their expression levels and annotation, that were found to be differentially expressed in healthy ant heads at 2 different time points during the day; 10:00 h and 14:00 h (sheets 2 and 3), during manipulated biting behavior in comparison to healthy ant heads at 10:00 h (sheets 4 and 5), after manipulated biting behavior in comparison to healthy ant heads at 14:00 h (sheets 6 and 7), and during manipulated biting behavior versus death after manipulated biting behavior (sheets 8 and 9). (XLSX 339 kb) Additional file 11:
**Enrichment analysis on differentially expressed** ***C. castaneus*** **genes.** Tables summarizing the overrepresentation of annotation terms for *C. castaneus* genes found to be up- and down-regulated when healthy ants are compared at two different times of day; 10 AM and 2 PM (**Tables S1 and S2**), during manipulated biting behavior in comparison to healthy ant controls (**Tables S3 and S4**), after manipulated biting behavior in comparison to healthy ant controls (**Tables S5 and S6**), during manipulated biting behavior versus death after manipulated biting behavior (**Tables S7 and S8**), and genes that are uniquely up- or down-regulated during manipulated biting behavior (**Tables S9 and S10**). (PDF 554 kb)
