# Supplementary material for: Gene expression during zombie ant biting behavior reflects the complexity underlying fungal parasitic behavioral manipulation
Source: BMC Genomics. 2015 Aug 19;16(1):620. doi: 10.1186/s12864-015-1812-x (PMC4545319; doi:10.1186/s12864-015-1812-x)
Supplement: Additional file 1: — KEGG annotations of genes found in the O. unilateralis s.l. genome. Pie charts representing the first and second level KEGG annotations that were found in the O. unilateralis s.l. genome. First level annotations are indicated in the multi-color pie chart in the center. Second level annotations are indicated in the surrounding pie charts, displayed in different shades of the color of their first level annotation. (PDF 377 kb) [file 12864_2015_1812_MOESM1_ESM.pdf]

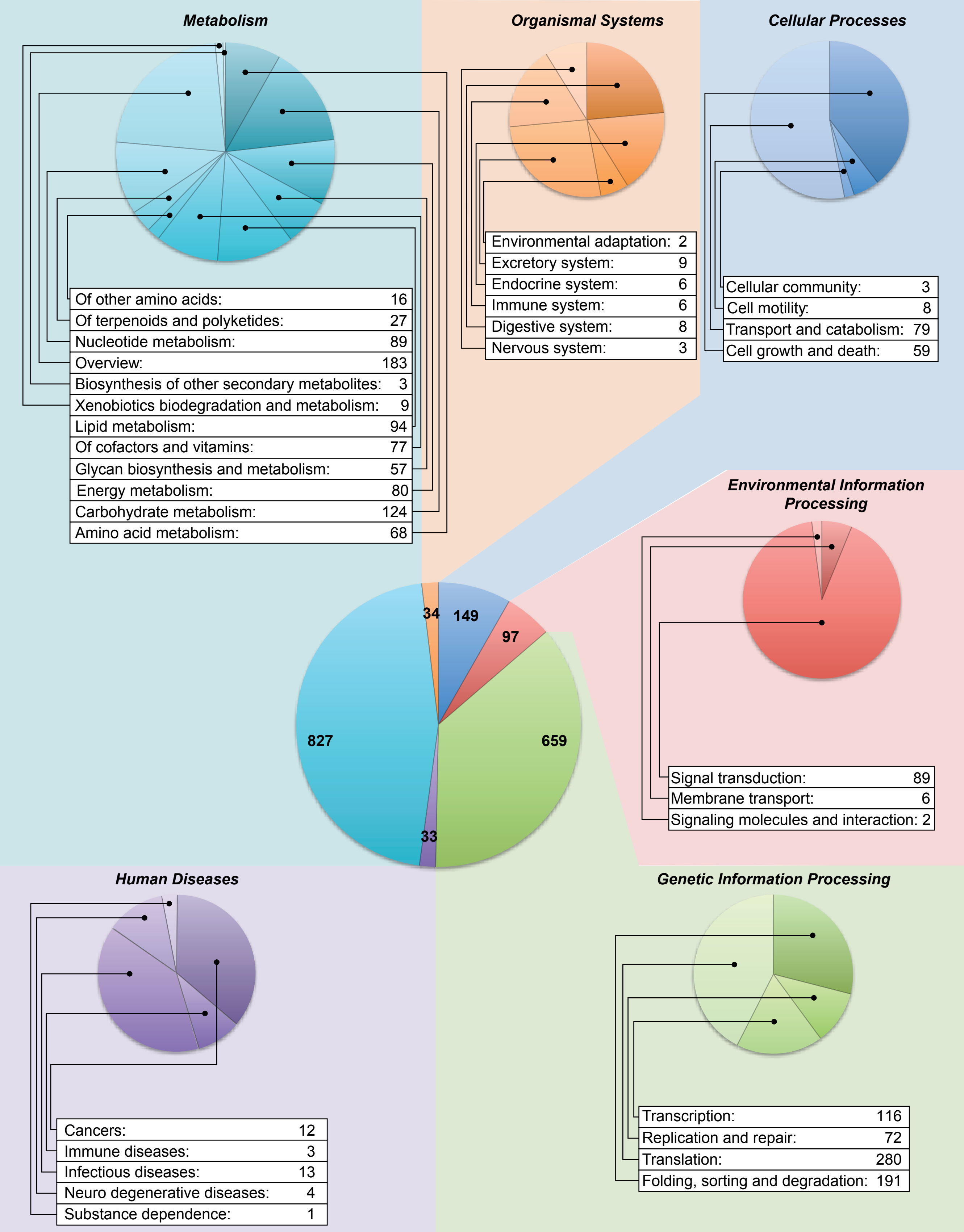

Metabolism
Of other amino acids: 16
Of terpenoids and polyketides: 27
Nucleotide metabolism: 89
Overview: 183
Biosynthesis of other secondary metabolites: 3
Xenobiotics biodegradation and metabolism: 9
Lipid metabolism: 94
Of cofactors and vitamins: 77
Glycan biosynthesis and metabolism: 57
Energy metabolism: 80
Carbohydrate metabolism: 124
Amino acid metabolism: 68
Organismal Systems
Environmental adaptation: 2
Excretory system: 9
Endocrine system: 6
Immune system: 6
Digestive system: 8
Nervous system: 3
Cellular Processes
Cellular community: 3
Cell motility: 8
Transport and catabolism: 79
Cell growth and death: 59
Environmental Information Processing
Signal transduction: 89
Membrane transport: 6
Signaling molecules and interaction: 2
34
149
97
827
659
33
Human Diseases
Cancers: 12
Immune diseases: 3
Infectious diseases: 13
Neuro degenerative diseases: 4
Substance dependence: 1
Genetic Information Processing
Transcription: 116
Replication and repair: 72
Translation: 280
Folding, sorting and degradation: 191
