# Supplementary figures and images for: Gene expression during zombie ant biting behavior reflects the complexity underlying fungal parasitic behavioral manipulation
Source: BMC Genomics. 2015 Aug 19;16(1):620. doi: 10.1186/s12864-015-1812-x (PMC4545319; doi:10.1186/s12864-015-1812-x)

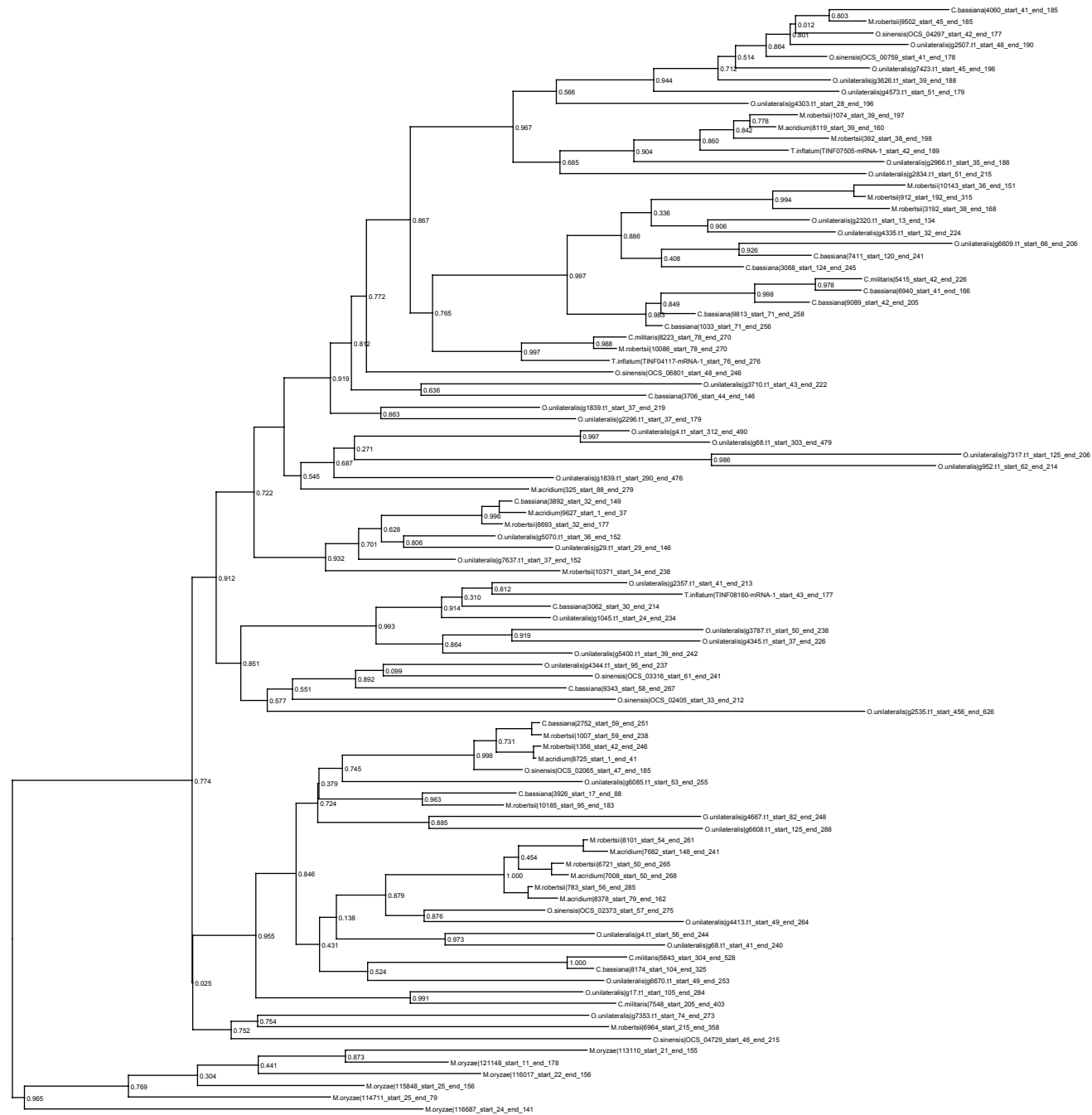

Supplement: Additional file 2: — Phylogenetic relationship between genes annotated to have an enterotoxin_a PFAM domain. Phylogenetic tree of all genes in this study that contain one or more enterotoxin_a PFAM domains. The tree was rooted on M. oryzae enterotoxins. (PDF 190 kb) [file 12864_2015_1812_MOESM2_ESM.pdf]

**A**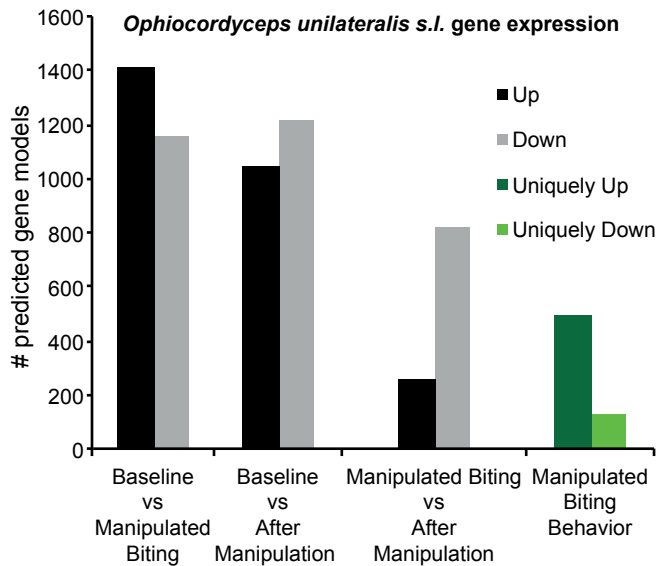**B**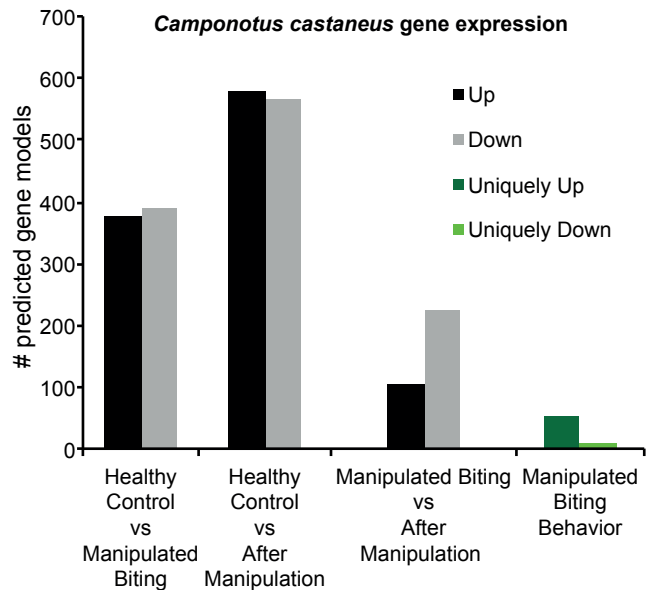

Supplement: Additional file 4: — Differential gene expression in parasite and host during and after manipulated biting behavior. Bar charts displaying the number of genes that are up- and down- regulated during the parasite-host interaction. (A) The fungal parasite O. unilateralis s.l. gene expression during and after manipulated biting behavior are both compared to a baseline gene expression displayed by the fungus in insect cell culture media and to each other. (B) The ant host C. castaneus gene expression during and after manipulated biting behavior are both compared to the gene expression in healthy hosts sampled at the same time of day and to each other. The amount of genes that are differentially expressed during manipulated biting compared to both the situation before (controls) and after the event are indicated in green (Uniquely Up, − Down). Genes were considered differentially expressed when expression was at least 4 FPKM for one of the sample types and significantly (Q < 0.05) changed > =2-fold. (PDF 157 kb) [file 12864_2015_1812_MOESM4_ESM.pdf]

**A**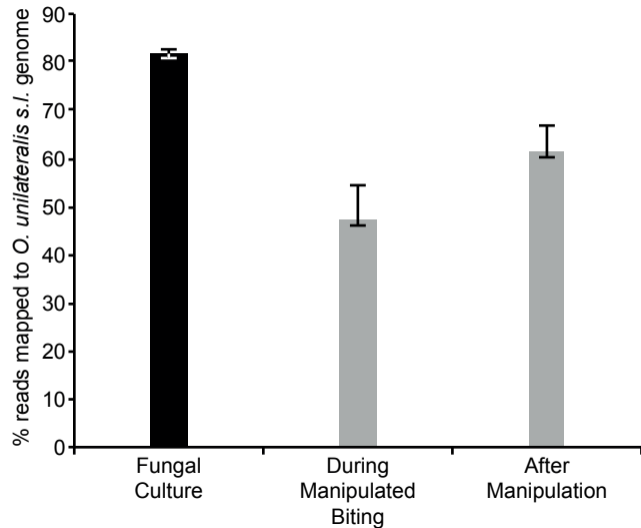**B**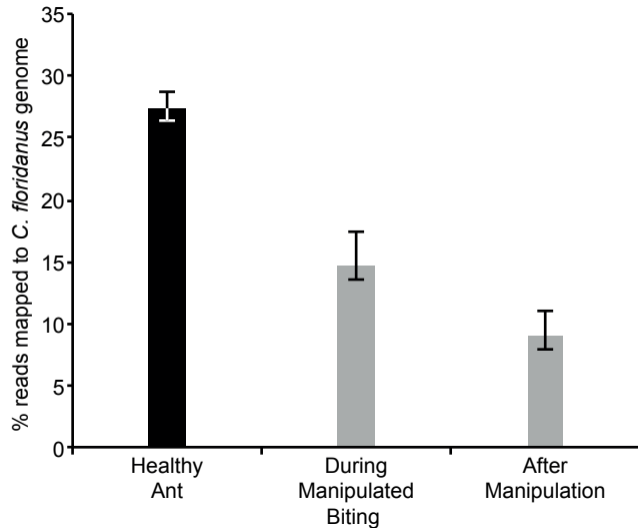

Supplement: Additional file 5: — Mapped fungal and insect reads in mixed transcriptome samples compared to single transcriptome controls. Bar charts displaying the percentage of RNA-Seq reads in both single and mixed transcriptome samples mapped to either the parasitic O. unilateralis s.l. genome (A) or the C. floridanus genome (B). The single transcriptome samples (Fungal Culture and Healthy Ant heads; black bars) display the read mapping when all of the biological material is derived from one organism. Relative comparison to this percentage reads mapped suggests that during manipulated biting behavior about half of the biological material is ant tissue, while the other half comprises of fungal cells. Right after the manipulated biting event the amount of fungal cells inside the head appears to have increased, while the amount of host tissue has decreased even further. (PDF 131 kb) [file 12864_2015_1812_MOESM5_ESM.pdf]

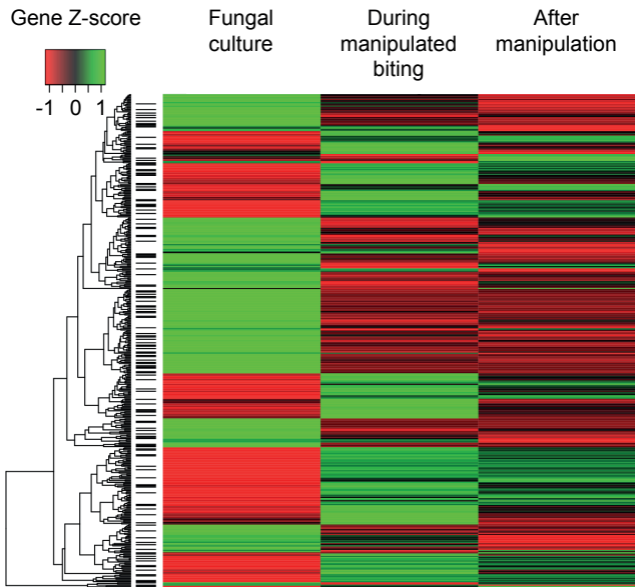

Supplement: Additional file 9: — An expression heat map of differentially expressed O. unilateralis s.l. genes that contain a putative secretion signal. Of 891 genes encoding a protein with a putative secretion signal, 429 (48 %) were differentially expressed. Green indicates a relatively high expression of that gene compared to other conditions, whereas red indicates relatively low expression. Genes are clustered based on similarity of expression profile. The large differences in expression between the samples are clearly visible. Genes annotated to be small secreted proteins (SSPs) are indicated with a line in between the dendrogram and the heat map. (PDF 538 kb) [file 12864_2015_1812_MOESM9_ESM.pdf]
