# Supplementary material for: Gene expression during zombie ant biting behavior reflects the complexity underlying fungal parasitic behavioral manipulation
Source: BMC Genomics. 2015 Aug 19;16(1):620. doi: 10.1186/s12864-015-1812-x (PMC4545319; doi:10.1186/s12864-015-1812-x)
Supplement: Additional file 3: — Mixed and single transcriptome sequencing and genome mapping statistics. Table summarizing the sequencing statistics for each transcriptomics sample used in this study as well as the percentage of reads that mapped to the respective parasite and host reference genomes. For all samples, 100 % of the clusters passed filtering and 100 % of the reads in the sample matched the given index. (PDF 90 kb) [file 12864_2015_1812_MOESM3_ESM.pdf]

Lane	Sample ID	Description	Yield (Mbases)	# Reads	% of raw clusters per lane	% of >= Q30 Bases (PF)	Mean Quality Score (PF)	% Reads mapping to *O. unilateralis s.l.* genome	% Reads mapping to *C. floridanus* genome
1	13SC16a	*O. unilateralis s.l.* control	1,425	14,251,542	5.26	88.42	34.26	81.1	n/a
2	13SC16a	*O. unilateralis s.l.* control	1,444	14,442,488	5.33	88.41	34.26		
1	14SC16a	*O. unilateralis s.l.* control	2,068	20,683,990	7.64	88.37	34.26	82.0	n/a
2	14SC16a	*O. unilateralis s.l.* control	2,094	20,943,060	7.72	88.38	34.27		
1	15SC16a	*O. unilateralis s.l.* control	1,874	18,743,720	6.92	88.43	34.28	81.1	n/a
2	15SC16a	*O. unilateralis s.l.* control	1,887	18,865,090	6.96	88.42	34.28		
1	10HA2	*C. castaneus* control at 2PM	1,741	17,406,504	6.43	92.53	35.93	n/a	27.3
2	10HA2	*C. castaneus* control at 2PM	1,731	17,312,344	6.38	92.35	35.88		
1	11HA2	*C. castaneus* control at 2PM	1,808	18,083,132	6.68	92.42	35.91	n/a	26.9
2	11HA2	*C. castaneus* control at 2PM	1,798	17,984,200	6.63	92.22	35.86		
1	12HA2	*C. castaneus* control at 2PM	1,376	13,761,068	5.08	92.45	35.9	n/a	28.8
2	12HA2	*C. castaneus* control at 2PM	1,367	13,671,646	5.04	92.27	35.85		
1	7HA10	*C. castaneus* control at 10AM	1,176	11,757,876	4.34	92.3	35.83	n/a	29.0
2	7HA10	*C. castaneus* control at 10AM	1,173	11,729,986	4.33	92.13	35.78		
1	8HA10	*C. castaneus* control at 10AM	1,801	18,005,462	6.65	92.43	35.88	n/a	25.7
2	8HA10	*C. castaneus* control at 10AM	1,788	17,883,278	6.59	92.28	35.84		
1	9HA10	*C. castaneus* control at 10AM	2,046	20,460,248	7.56	91.9	35.73	n/a	26.3
2	9HA10	*C. castaneus* control at 10AM	2,034	20,344,976	7.5	91.73	35.68		
1	1Cc6L	live infected ant head during manipulation	1,838	18,384,106	6.79	89.88	34.87	45.6	15.7
2	1Cc6L	live infected ant head during manipulation	1,847	18,470,768	6.81	89.77	34.84		
1	2Cc7L	live infected ant head during manipulation	1,857	18,566,820	6.86	89.55	34.77	40.2	16.8
2	2Cc7L	live infected ant head during manipulation	1,861	18,607,708	6.86	89.42	34.73		
1	3Cc8L	live infected ant head during manipulation	1,808	18,079,164	6.68	88.59	34.42	55.0	11.3
2	3Cc8L	live infected ant head during manipulation	1,813	18,129,654	6.69	88.52	34.4		
1	4Cc3D	dead infected ant head after manipulation	1,793	17,929,616	6.62	88.83	34.44	67.1	6.8
2	4Cc3D	dead infected ant head after manipulation	1,805	18,053,878	6.66	88.79	34.43		
1	5Cc4D	dead infected ant head after manipulation	1,700	16,996,412	6.28	88.76	34.44	59.8	9.4
2	5Cc4D	dead infected ant head after manipulation	1,714	17,142,746	6.32	88.7	34.42		
1	6Cc5D	dead infected ant head after manipulation	1,909	19,094,148	7.05	88.73	34.45	56.3	10.9
2	6Cc5D	dead infected ant head after manipulation	1,915	19,153,566	7.06	88.67	34.44		
1	lane1	Clusters with unmatched barcodes for lane 1	849	8,492,704	3.14	79.59	31.68	n/a	n/a
2	lane2	Clusters with unmatched barcodes for lane 2	846	8,462,100	3.12	80.02	31.83	n/a	n/a
