# Supplementary material for: Gene expression during zombie ant biting behavior reflects the complexity underlying fungal parasitic behavioral manipulation
Source: BMC Genomics. 2015 Aug 19;16(1):620. doi: 10.1186/s12864-015-1812-x (PMC4545319; doi:10.1186/s12864-015-1812-x)
Supplement: Additional file 6: — Enrichment analysis on differentially expressed O. unilateralis s.l. genes. Tables summarizing the overrepresentation of annotation terms for O. unilateralis s.l. genes found to be up- and down-regulated during manipulated biting behavior in comparison to baseline gene expression in fungal cultures (Tables S1 and S2, respectively), after manipulated biting behavior in comparison to baseline gene expression (Tables S3 and S4), during manipulated biting behavior versus death after manipulated biting behavior (Tables S5 and S6), and genes that are uniquely up- or down-regulated during manipulated biting behavior (Tables S7 and S8). (PDF 263 kb) [file 12864_2015_1812_MOESM6_ESM.pdf]

Baseline Culture vs Manipulated Biting Behavior

Table 1: Overrepresentation of annotation terms within the total of 1417 up-regulated *Ophiocordyceps unilateralis s.l.* genes (2-fold and significant Q<0.05, minimum expression of 4) in live manipulated *Camponotus castaneus* ant heads (Manipulated Biting Behavior samples) compared to gene expression in culture (Fungal Baseline Expression samples)

GO Annotation term	# up-regulated proteins with annotation term	# up-regulated proteins without annotation term	# other proteins with annotation term	# other proteins without annotation term	log odds ratio	p-value (uncorrected)	p-value (corrected)
GO:0006259|DNA metabolic process	50	1367	74	6340	1.14221812293	4.98230015171e-09	4.43424713502e-06
GO:0008150|biological_process	566	851	2093	4321	0.317070363116	1.87742447616e-07	6.52162866388e-05
GO:0006260|DNA replication	19	1398	13	6401	1.90090120605	2.19830179682e-07	6.52162866388e-05
GO:0044699|single-organism process	282	1135	936	5478	0.374399014368	1.12665033172e-06	0.000250679698809
GO:0016491|oxidoreductase activity	132	1285	381	6033	0.486488228571	7.99075905152e-06	0.00130964768009
GO:0008152|metabolic process	403	1014	1466	4948	0.293724228861	1.19839023669e-05	0.00152366758665
GO:0055114|oxidation-reduction process	110	1307	307	6107	0.515333837804	1.48655626704e-05	0.00165379384708
GO:0003677|DNA binding	73	1344	183	6231	0.614859879768	3.19129477623e-05	0.00315583594539
GO:0090304|nucleic acid metabolic process	91	1326	261	6153	0.481111975189	0.000215577843582	0.0147587908299
GO:0046906|tetrapyrrole binding	34	1383	70	6344	0.801119714217	0.000280017168942	0.0166143520239
GO:0020037|heme binding	34	1383	70	6344	0.801119714217	0.000280017168942	0.0166143520239
GO:0003824|catalytic activity	413	1004	1587	4827	0.224080007843	0.000672328428995	0.0351983706945

PFAM Annotation term	# up-regulated proteins with annotation term	# up-regulated proteins without annotation term	# other proteins with annotation term	# other proteins without annotation term	log odds ratio	p-value (uncorrected)	p-value (corrected)
PF00493.18 MCM2/3/5 family	6	1411	0	6414	NaN	3.47972191271e-05	0.0149280070055
PF14551.1 MCM N-terminal domain	6	1411	1	6413	3.30578797652	0.000205932833298	0.0441725927424

TF Annotation term	# up-regulated proteins with annotation term	# up-regulated proteins without annotation term	# other proteins with annotation term	# other proteins without annotation term	log odds ratio	p-value (uncorrected)	p-value (corrected)
TF: Heteromeric CCAAT factors	6	1411	4	6410	1.91902570617	0.00374884463722	0.0449861356467

Secretome Annotation term	# up-regulated proteins with annotation term	# up-regulated proteins without annotation term	# other proteins with annotation term	# other proteins without annotation term	log odds ratio	p-value (uncorrected)	p-value (corrected)
secretome<br>Has a SignalP signal sequence	191	1226	700	5714	0.240355559298	0.00728703327334	0.00728703327334

KEGG Annotation term	# up-regulated proteins with annotation term	# up-regulated proteins without annotation term	# other proteins with annotation term	# other proteins without annotation term	log odds ratio	p-value (uncorrected)	p-value (corrected)
KEGG third level: DNA replication	24	1393	8	6406	2.62438764506	3.02851754904e-12	9.44897475301e-10
KEGG second level: Replication and repair	43	1374	54	6360	1.30451825268	1.76355254575e-09	2.75114197137e-07
KEGG third level: Mismatch repair	13	1404	9	6405	1.88547840987	2.06644508055e-05	0.00128946173026
KEGG third level: Cell cycle - yeast	28	1389	45	6369	1.04840042802	4.18195223335e-05	0.00217461516134
KEGG third level: Cell cycle	21	1396	31	6383	1.13056243517	0.000166773239788	0.00743332154485
KEGG third level: Homologous recombination	10	1407	7	6407	1.87260630846	0.00020559123882	0.00801805831396
KEGG EC: EC:3.6.4.12	8	1409	5	6409	1.98482664885	0.000610995888747	0.0160636811794
KEGG second level: Cell growth and death	34	1383	75	6339	0.731338385699	0.000956305820871	0.0225361965483
KEGG third level: Meiosis - yeast	20	1397	34	6380	0.98821276602	0.00101123958871	0.0225361965483
KEGG third level: Cyanoamino acid metabolism	7	1410	5	6409	1.85058578475	0.00211874797688	0.0388852569874
KEGG third level: Pyrimidine metabolism	20	1397	37	6377	0.903185047968	0.00264923631153	0.0435032489051
KEGG third level: Alcoholism	9	1408	10	6404	1.40939202174	0.00326913488038	0.0485700039371

Table 2: Overrepresentation of annotation terms within the total of 1153 down-regulated *Ophiocordyceps unilateralis s.l.* genes (2-fold and significant Q<0.05, minimum expression of 4) in live manipulated *Camponotus castaneus* ant heads (Manipulated Biting Behavior samples) compared to gene expression in culture (Fungal Baseline Expression samples)

**GO Annotation term**	**# down-regulated proteins with annotation term**	**# down-regulated proteins without annotation term**	**# other proteins with annotation term**	**# other proteins without annotation term**	**log odds ratio**	**p-value (uncorrected)**	**p-value (corrected)**
GO:0005975|carbohydrate metabolic process	42	1111	116	6562	0.760114351058	9.08584012242e-05	0.00577599836354
GO:0006006|glucose metabolic process	5	1148	2	6676	2.67678843978	0.0011118507602	0.0299862780782
GO:0019318|hexose metabolic process	7	1146	7	6671	1.76149215543	0.00195447101754	0.0483188668225

**SSP Annotation term**	**# down-regulated proteins with annotation term**	**# down-regulated proteins without annotation term**	**# other proteins with annotation term**	**# other proteins without annotation term**	**log odds ratio**	**p-value (uncorrected)**	**p-value (corrected)**
SSP<br>Small Secreted Protein	63	1090	235	6443	0.460365786163	0.00256692912628	0.00256692912628

**secretome Annotation term**	**# down-regulated proteins with annotation term**	**# down-regulated proteins without annotation term**	**# other proteins with annotation term**	**# other proteins without annotation term**	**log odds ratio**	**p-value (uncorrected)**	**p-value (corrected)**
secretome<br>Has a SignalP signal sequence	169	984	722	5956	0.348402072782	0.000239041976078	0.000239041976078

**TMHMM Annotation term**	**# down-regulated proteins with annotation term**	**# down-regulated proteins without annotation term**	**# other proteins with annotation term**	**# other proteins without annotation term**	**log odds ratio**	**p-value (uncorrected)**	**p-value (corrected)**
TMHMM<br>Has transmembrane domain (TMHMM)	246	907	1247	5431	0.166571698172	0.0348129915018	0.0348129915018

KEGG Annotation term	# down-regulated proteins with annotation term	# down-regulated proteins without annotation term	# other proteins with annotation term	# other proteins without annotation term	log odds ratio	p-value (uncorrected)	p-value (corrected)
KEGG second level: Xenobiotics biodegradation and metabolism	21	1132	37	6641	1.20288109986	4.01972361973e-05	0.00209025628226
KEGG third level: Pentose phosphate pathway	10	1143	9	6669	1.8691740545	0.000119381274309	0.00465586969806
KEGG third level: Carbon fixation in photosynthetic organisms	9	1144	7	6671	2.01455330905	0.000135492869062	0.00469708612749
KEGG second level: Carbohydrate metabolism	48	1105	156	6522	0.596680746056	0.000859074294548	0.0243664708999
KEGG third level: Carotenoid biosynthesis	4	1149	1	6677	3.14607114713	0.00206458743471	0.0460108056877
KEGG third level: Caprolactam degradation	4	1149	1	6677	3.14607114713	0.00206458743471	0.0460108056877

Baseline Culture vs Death After Manipulated Biting Behavior

Table 3: Overrepresentation of annotation terms within the total of 1050 up-regulated *Ophiocordyceps unilateralis* genes (2-fold and significant Q<0.05, minimum expression of 4) in dead *Camponotus castaneus* ant heads after manipulation (Death After Manipulation samples) compared to gene expression in culture (Fungal Baseline Expression samples)

GO Annotation term	# up-regulated proteins with annotation term	# up-regulated proteins without annotation term	# other proteins with annotation term	# other proteins without annotation term	log odds ratio	p-value (uncorrected)	p-value (corrected)
GO:0008150|biological_process	419	631	2240	4541	0.29723644363	1.61726975844e-05	0.00719685042508
GO:0006259|DNA metabolic process	35	1015	89	6692	0.952735862388	1.23541224104e-05	0.00719685042508
GO:0006260|DNA replication	13	1037	19	6762	1.49549715333	0.000123319793546	0.0245190864968
GO:0008152|metabolic process	301	749	1568	5213	0.289725865089	0.00011587755697	0.0245190864968

KEGG Annotation term	# up-regulated proteins with annotation term	# up-regulated proteins without annotation term	# other proteins with annotation term	# other proteins without annotation term	log odds ratio	p-value (uncorrected)	p-value (corrected)
KEGG third level: DNA replication	19	1031	13	6768	2.26116603897	1.39354422702e-09	4.34785798832e-07
KEGG second level: Replication and repair	30	1020	67	6714	1.08089703353	6.19996550035e-06	0.000386877847222
KEGG third level: Mismatch repair	12	1038	10	6771	2.05767455855	5.6197539803e-06	0.000386877847222
KEGG third level: Cell cycle - yeast	25	1025	48	6733	1.23000301093	4.33903310599e-06	0.000386877847222
KEGG third level: Alcoholism	9	1041	10	6771	1.76710648121	0.000353561337683	0.0157587339082
KEGG second level: Cell growth and death	28	1022	81	6700	0.818101390118	0.000538365421242	0.0209962514284
KEGG third level: Valine, leucine and isoleucine biosynthesis	7	1043	6	6775	2.02528887057	0.000626842384832	0.0217305360075
KEGG third level: Cell cycle	16	1034	36	6745	1.06443649736	0.0014358949918	0.0447999237442

Table 4: Overrepresentation of annotation terms within the total of 1218 down-regulated *Ophiocordyceps unilateralis* genes (2-fold and significant Q<0.05, minimum expression of 4) in dead *Camponotus castaneus* ant heads after manipulation (Death After Manipulation samples) compared to gene expression in culture (Fungal Baseline Expression samples)

**GO Annotation term**	**# down-regulated proteins with annotation term**	**# down-regulated proteins without annotation term**	**# other proteins with annotation term**	**# other proteins without annotation term**	**log odds ratio**	**p-value (uncorrected)**	**p-value (corrected)**
GO:0044765|single-organism transport	73	1145	263	6350	0.431355584869	0.00204705200796	0.0267922983395
GO:0055085|transmembrane transport	63	1155	203	6410	0.543687674309	0.000392563933064	0.00727878959223
GO:0016641|oxidoreductase activity, acting on the CH-NH2 group of donors, oxygen as acceptor	5	1213	1	6612	3.30522746243	0.000472305349027	0.00824219138498
GO:0009405|pathogenesis	13	1205	22	6591	1.17313241923	0.00150694301696	0.0206335274631
GO:0051704|multi-organism process	13	1205	23	6590	1.1285289231	0.00202815207176	0.0267922983395

**PFAM Annotation term**	**# down-regulated proteins with annotation term**	**# down-regulated proteins without annotation term**	**# other proteins with annotation term**	**# other proteins without annotation term**	**log odds ratio**	**p-value (uncorrected)**	**p-value (corrected)**
PF07690.11 Major Facilitator Superfamily	33	1185	75	6538	0.886907981645	7.57319364126e-05	0.032489000721
PF00083.19 Sugar (and other) transporter	22	1196	43	6570	1.03337351463	0.000213372924259	0.0457684922535

**SSP Annotation term**	**# down-regulated proteins with annotation term**	**# down-regulated proteins without annotation term**	**# other proteins with annotation term**	**# other proteins without annotation term**	**log odds ratio**	**p-value (uncorrected)**	**p-value (corrected)**
SSP<br>Small Secreted Protein	67	1151	231	6382	0.475125306985	0.00140259440063	0.00140259440063

**Secretome Annotation term**	**# down-regulated proteins with annotation term**	**# down-regulated proteins without annotation term**	**# other proteins with annotation term**	**# other proteins without annotation term**	**log odds ratio**	**p-value (uncorrected)**	**p-value (corrected)**
secretome<br>Has a SignalP signal sequence	188	1030	703	5910	0.428172100196	2.88772207093e-06	2.88772207093e-06

TMHMM Annotation term	# down-regulated proteins with annotation term	# down-regulated proteins without annotation term	# other proteins with annotation term	# other proteins without annotation term	log odds ratio	p-value (uncorrected)	p-value (corrected)
TMHMM<br>Has transmembrane domain (TMHMM)	290	928	1203	5410	0.34027984606	7.10575527309e-06	7.10575527309e-06

KEGG Annotation term	# down-regulated proteins with annotation term	# down-regulated proteins without annotation term	# other proteins with annotation term	# other proteins without annotation term	log odds ratio	p-value (uncorrected)	p-value (corrected)
KEGG third level: Valine, leucine and isoleucine degradation	12	1206	17	6596	1.35084761212	0.000746156946118	0.0145500604493

Manipulated Biting Behavior vs Death After Manipulated Biting Behavior

Table 5: Overrepresentation of annotation terms within the total of 259 up-regulated *Ophiocordyceps unilateralis* genes (2-fold and significant Q<0.05, minimum expression of 4) in dead *Camponotus castaneus* ant heads after manipulation (Death After Manipulation samples) compared to gene expression in live manipulated *Camponotus castaneus* ant heads (Manipulated Biting Behavior samples)

GO Annotation term	# up-regulated proteins with annotation term	# up-regulated proteins without annotation term	# other proteins with annotation term	# other proteins without annotation term	log odds ratio	p-value (uncorrected)	p-value (corrected)
GO:0006928|cellular component movement	4	255	7	7565	2.83040829313	0.000321095077178	0.0408249455269
GO:0016853|isomerase activity	7	252	30	7542	1.9435263577	0.00017549125834	0.0408249455269
GO:0007018|microtubule-based movement	4	255	7	7565	2.83040829313	0.000321095077178	0.0408249455269
GO:0005975|carbohydrate metabolic process	15	244	143	7429	1.1611838847	0.000219924957627	0.0408249455269
GO:0003777|microtubule motor activity	4	255	7	7565	2.83040829313	0.000321095077178	0.0408249455269
GO:0044723|single-organism carbohydrate metabolic process	9	250	57	7515	1.55736869383	0.000292764990053	0.0408249455269
GO:0003774|motor activity	5	254	11	7561	2.60496710818	0.000123271054262	0.0408249455269

Secretome Annotation term	# up-regulated proteins with annotation term	# up-regulated proteins without annotation term	# other proteins with annotation term	# other proteins without annotation term	log odds ratio	p-value (uncorrected)	p-value (corrected)
secretome<br>Has a SignalP signal sequence	47	212	844	6728	0.569442034095	0.000967872785157	0.000967872785157

KEGG Annotation term	# up-regulated proteins with annotation term	# up-regulated proteins without annotation term	# other proteins with annotation term	# other proteins without annotation term	log odds ratio	p-value (uncorrected)	p-value (corrected)
KEGG third level: Glycolysis / Gluconeogenesis	7	252	22	7550	2.25474145043	3.33654612578e-05	0.0104100239124
KEGG second level: Carbohydrate metabolism	18	241	186	7386	1.0871697461	0.000134385855928	0.0209641935247

Table 6: Overrepresentation of annotation terms within the total of 821 down-regulated *Ophiocordyceps unilateralis* genes (2-fold and significant Q<0.05, minimum expression of 4) in dead *Camponotus castaneus* ant heads after manipulation (Death After Manipulation samples) compared to gene expression in live manipulated *Camponotus castaneus* ant heads (Manipulated Biting Behavior samples)

GO Annotation term	# down-regulated proteins with annotation term	# down-regulated proteins without annotation term	# other proteins with annotation term	# other proteins without annotation term	log odds ratio	p-value (uncorrected)	p-value (corrected)
GO:0016491|oxidoreductase activity	101	720	412	6598	0.80936780727	1.23102469289e-10	5.47805988334e-08
GO:0055114|oxidation-reduction process	85	736	332	6678	0.842859989349	6.6669028379e-10	1.97784784191e-07
GO:0005506|iron ion binding	28	793	53	6957	1.53359300091	4.63477422448e-09	1.03123726495e-06
GO:0044699|single-organism process	181	640	1037	5973	0.487946239415	1.8836863515e-07	2.13712262405e-05
GO:0016705|oxidoreductase activity, acting on paired donors, with incorporation or reduction of molecular oxygen	26	795	61	6949	1.31523360251	4.57484932146e-07	3.81213993388e-05
GO:0046906|tetrapyrrole binding	29	792	75	6935	1.21958265738	5.1399639558e-07	3.81213993388e-05
GO:0020037|heme binding	29	792	75	6935	1.21958265738	5.1399639558e-07	3.81213993388e-05
GO:0055085|transmembrane transport	54	767	212	6798	0.814294700781	1.23186127613e-06	8.43351181354e-05
GO:0006260|DNA replication	13	808	19	6991	1.77832720629	8.74303781659e-06	0.000409542297725
GO:0044710|single-organism metabolic process	123	698	689	6321	0.480356684069	1.22136957068e-05	0.000543509458951
GO:0044765|single-organism transport	61	760	275	6735	0.675857422071	1.51265912288e-05	0.000641079342553
GO:0003824|catalytic activity	261	560	1739	5271	0.345493486094	2.26081590126e-05	0.000914602796417
GO:0008152|metabolic process	243	578	1626	5384	0.330796155885	6.71077802558e-05	0.00248858018449
GO:0046914|transition metal ion binding	60	761	287	6723	0.61351875107	7.20675719041e-05	0.00256560555979
GO:0016021|integral component of membrane	52	769	239	6771	0.65009326263	0.000120569497897	0.00383238761171
GO:0008150|biological_process	328	493	2331	4679	0.289291486172	0.000155603589255	0.00477542049781
GO:0006810|transport	67	754	362	6648	0.489727376093	0.000831524380188	0.0205571305102
GO:0051234|establishment of localization	67	754	365	6645	0.481022868948	0.000873736509789	0.0210169052355
GO:0016701|oxidoreductase activity, acting on single donors with incorporation of molecular oxygen	6	815	7	7003	1.99675511459	0.00116963146842	0.0273940001813
GO:0006259|DNA metabolic process	25	796	99	6911	0.785026412981	0.0015622020603	0.0356502521453
GO:0015994|chlorophyll metabolic process	4	817	1	7009	3.53560558276	0.000549993855775	0.0143968979894
GO:0022857|transmembrane transporter activity	37	784	174	6836	0.617411637315	0.00188128892344	0.0398654081396
GO:0046148|pigment biosynthetic process	5	816	5	7005	2.14996510381	0.00200910027597	0.0415837033863

PFAM Annotation term	# down-regulated proteins with annotation term	# down-regulated proteins without annotation term	# other proteins with annotation term	# other proteins without annotation term	log odds ratio	p-value (uncorrected)	p-value (corrected)
PF07690.11 Major Facilitator Superfamily	30	791	78	6932	1.21509423804	3.58702282228e-07	7.69416395379e-05
PF00067.17 Cytochrome P450	21	800	38	6972	1.57198195697	2.24944357673e-07	7.69416395379e-05
PF00493.18 MCM2/3/5 family	6	815	0	7010	NaN	1.30627662882e-06	0.000186797557921
PF14551.1 MCM N-terminal domain	6	815	1	7009	3.94352167249	8.3276178439e-06	0.000893137013758
PF01494.14 FAD binding domain	9	812	11	6999	1.95335152507	8.44602032234e-05	0.00724668543657

SSP Annotation term	# down-regulated proteins with annotation term	# down-regulated proteins without annotation term	# other proteins with annotation term	# other proteins without annotation term	log odds ratio	p-value (uncorrected)	p-value (corrected)
SSP<br>Small Secreted Protein	56	765	242	6768	0.716499032038	1.12280474771e-05	1.12280474771e-05

Secretome Annotation term	# down-regulated proteins with annotation term	# down-regulated proteins without annotation term	# other proteins with annotation term	# other proteins without annotation term	log odds ratio	p-value (uncorrected)	p-value (corrected)
secretome<br>Has a SignalP signal sequence	145	676	746	6264	0.588389303844	1.43741293616e-08	1.43741293616e-08

TMHMM Annotation term	# down-regulated proteins with annotation term	# down-regulated proteins without annotation term	# other proteins with annotation term	# other proteins without annotation term	log odds ratio	p-value (uncorrected)	p-value (corrected)
TMHMM<br>Has transmembrane domain (TMHMM)	199	622	1294	5716	0.345895795974	0.000114175500511	0.000114175500511

KEGG Annotation term	# down-regulated proteins with annotation term	# down-regulated proteins without annotation term	# other proteins with annotation term	# other proteins without annotation term	log odds ratio	p-value (uncorrected)	p-value (corrected)
KEGG third level: DNA replication	15	806	17	6993	2.03541804224	1.82743060976e-07	1.90052783415e-05
KEGG second level: Biosynthesis of other secondary metabolites	11	810	22	6988	1.46176824303	0.00034710909788	0.0120331153932
KEGG EC: EC:3.6.4.12	6	815	7	7003	1.99675511459	0.00116963146842	0.0260660727247
KEGG third level: Valine, leucine and isoleucine degradation	9	812	20	6990	1.35422779885	0.00211387616001	0.0412205851202

secondary metabolism Annotation term	# down-regulated proteins with annotation term	# down-regulated proteins without annotation term	# other proteins with annotation term	# other proteins without annotation term	log odds ratio	p-value (uncorrected)	p-value (corrected)
Present in secondary metabolism cluster Backbone genes, decorating genes, and other genes in secondary metabolism clusters	34	787	93	6917	1.16727021165	1.59419281765e-07	3.18838563531e-06
Backbone genes and decorating genes Backbone genes and decorating genes in secondary metabolism clusters	22	799	55	6955	1.24756442672	7.88653354964e-06	7.88653354964e-05
Decorating Part of secondary metabolism cluster: Decorating	14	807	25	6985	1.57837810817	2.1287733216e-05	0.00014191822144
Cluster 2 Part of secondary metabolism cluster 2	6	815	2	7008	3.25023180804	3.0342448112e-05	0.00015171224056
Cluster 7 Part of secondary metabolism cluster 7	5	816	2	7008	3.06668400953	0.000219410990415	0.000877643961659
Cluster 14 Part of secondary metabolism cluster 14	3	818	2	7008	2.55341040412	0.00976050133005	0.0278871466573
NRPS Part of secondary metabolism cluster: NRPS	3	818	2	7008	2.55341040412	0.00976050133005	0.0278871466573
Cluster 18 Part of secondary metabolism cluster 18	4	817	6	7004	1.74313249044	0.0150142832726	0.0375357081814

Baseline Culture vs Manipulated Biting Behavior vs Death After Manipulated Biting Behavior

Table 7: Overrepresentation of annotation terms within the total of 498 *Ophiocordyceps unilateralis* genes that are up-regulated in live manipulated *Camponotus castaneus* ant heads (Manipulated Biting Behavior samples) compared to gene expression in culture (Fungal Baseline Expression samples) and down-regulated again after manipulation (Death After Manipulation samples) (2-fold and significant Q<0.05, minimum expression of 4)

GO Annotation term	# up-regulated proteins with annotation term	# up-regulated proteins without annotation term	# other proteins with annotation term	# other proteins without annotation term	log odds ratio	p-value (uncorrected)	p-value (corrected)
GO:0006260|DNA replication	13	485	19	7314	2.33390708607	2.70555528716e-08	2.40794420558e-05
GO:0005506|iron ion binding	19	479	62	7271	1.53725310936	4.90095168185e-07	0.000109046174921
GO:0016491|oxidoreductase activity	63	435	450	6883	0.795350994937	2.74427898953e-07	0.000109046174921
GO:0055114|oxidation-reduction process	54	444	363	6970	0.848127153622	4.28479303053e-07	0.000109046174921
GO:0006259|DNA metabolic process	24	474	100	7233	1.29808548951	7.22814267915e-07	0.000128660939689
GO:0016705|oxidoreductase activity, acting on paired donors, with incorporation or reduction of molecular oxygen	18	480	69	7264	1.37316506968	6.71425922519e-06	0.000669626059953
GO:0046906|tetrapyrrole binding	20	478	84	7249	1.28392354952	6.77149948267e-06	0.000669626059953
GO:0020037|heme binding	20	478	84	7249	1.28392354952	6.77149948267e-06	0.000669626059953
GO:0008150|biological_process	215	283	2444	4889	0.418542872772	1.0281652119e-05	0.000762555865491
GO:0008152|metabolic process	161	337	1708	5625	0.453219287356	1.00228906646e-05	0.000762555865491
GO:0044699|single-organism process	113	385	1105	6228	0.503354403427	1.68119737065e-05	0.00115097358453
GO:0003824|catalytic activity	166	332	1834	5499	0.404919703131	6.49662498955e-05	0.00385466416047
GO:0015994|chlorophyll metabolic process	4	494	1	7332	4.08376245289	7.67724137239e-05	0.00427046551339
GO:0044710|single-organism metabolic process	78	420	734	6599	0.512618488181	0.000138474021694	0.00724952231219
GO:0046914|transition metal ion binding	38	460	309	7024	0.63010652866	0.000967559054066	0.034923035487
GO:0044765|single-organism transport	37	461	299	7034	0.635587109291	0.00085642184068	0.034923035487
GO:0016798|hydrolase activity, acting on glycosyl bonds	13	485	64	7269	1.11329139265	0.00102022350861	0.034923035487
GO:0055085|transmembrane transport	31	467	235	7098	0.695640765879	0.00117921945857	0.0388705673382

PFAM Annotation term	# up-regulated proteins with annotation term	# up-regulated proteins without annotation term	# other proteins with annotation term	# other proteins without annotation term	log odds ratio	p-value (uncorrected)	p-value (corrected)
PF00493.18 MCM2/3/5 family	6	492	0	7333	NaN	6.42947040595e-08	2.75824280415e-05
PF14551.1 MCM N-terminal domain	6	492	1	7332	4.4932843617	4.25807597249e-07	9.13357296099e-05
PF00067.17 Cytochrome P450	15	483	44	7289	1.63796555491	2.68381670695e-06	0.000383785789093
PF07690.11 Major Facilitator Superfamily	17	481	91	7242	1.03413925776	0.000427661867086	0.045866735245

SSP Annotation term	# up-regulated proteins with annotation term	# up-regulated proteins without annotation term	# other proteins with annotation term	# other proteins without annotation term	log odds ratio	p-value (uncorrected)	p-value (corrected)
SSP<br>Small Secreted Protein	28	470	270	7063	0.443675025882	0.0383881417996	0.0383881417996

Secretome Annotation term	# up-regulated proteins with annotation term	# up-regulated proteins without annotation term	# other proteins with annotation term	# other proteins without annotation term	log odds ratio	p-value (uncorrected)	p-value (corrected)
secretome<br>Has a SignalP signal sequence	86	412	805	6528	0.526337566129	5.46630410752e-05	5.46630410752e-05

TMHMM Annotation term	# up-regulated proteins with annotation term	# up-regulated proteins without annotation term	# other proteins with annotation term	# other proteins without annotation term	log odds ratio	p-value (uncorrected)	p-value (corrected)
TMHMM<br>Has transmembrane domain (TMHMM)	118	380	1375	5958	0.296794494918	0.00792095738226	0.00792095738226

KEGG Annotation term	# up-regulated proteins with annotation term	# up-regulated proteins without annotation term	# other proteins with annotation term	# other proteins without annotation term	log odds ratio	p-value (uncorrected)	p-value (corrected)
KEGG third level: DNA replication	14	484	18	7315	2.46428297846	2.40713312015e-09	7.51025533485e-07
KEGG EC: EC:3.6.4.12	6	492	7	7326	2.54655554702	7.4962482681e-05	0.00477505086669
KEGG second level: Replication and repair	18	480	79	7254	1.23644612137	3.2320513966e-05	0.00477505086669

Secondary Metabolism Annotation term	# up-regulated proteins with annotation term	# up-regulated proteins without annotation term	# other proteins with annotation term	# other proteins without annotation term	log odds ratio	p-value (uncorrected)	p-value (corrected)
Present in secondary metabolism cluster<br>Backbone genes, decorating genes, and other genes in secondary metabolism clusters	20	478	107	7226	1.03873361831	0.000135728904546	0.00271457809091
Backbone genes and decorating genes<br>Backbone genes and decorating genes in secondary metabolism clusters	13	485	64	7269	1.11329139265	0.00102022350861	0.00680149005739
Cluster 2<br>Part of secondary metabolism cluster 2	4	494	4	7329	2.69705884274	0.00092071262013	0.00680149005739

Table 8: Overrepresentation of annotation terms within the total of 135 *Ophiocordyceps unilateralis* genes that are down-regulated in live manipulated *Camponotus castaneus* ant heads (Manipulated Biting Behavior samples) compared to gene expression in culture (Fungal Baseline Expression samples) and up-regulated again after manipulation (Death After Manipulation samples) (2-fold and significant Q<0.05, minimum expression of 4)

Go Annotation term	# down-regulated proteins with annotation term	# down-regulated proteins without annotation term	# other proteins with annotation term	# other proteins without annotation term	log odds ratio	p-value (uncorrected)	p-value (corrected)
GO:0005975|carbohydrate metabolic process	12	123	146	7550	1.618418515	1.63E-05	0.014498189

Secretome Annotation term	# down-regulated proteins with annotation term	# down-regulated proteins without annotation term	# other proteins with annotation term	# other proteins without annotation term	log odds ratio	p-value (uncorrected)	p-value (corrected)
secretome<br>Has a SignalP signal sequence	28	107	863	6833	0.728480079	0.001436723	0.001436723
