# Supplementary material for: Gene expression during zombie ant biting behavior reflects the complexity underlying fungal parasitic behavioral manipulation
Source: BMC Genomics. 2015 Aug 19;16(1):620. doi: 10.1186/s12864-015-1812-x (PMC4545319; doi:10.1186/s12864-015-1812-x)
Supplement: Additional file 11: — Enrichment analysis on differentially expressed C. castaneus genes. Tables summarizing the overrepresentation of annotation terms for C. castaneus genes found to be up- and down-regulated when healthy ants are compared at two different times of day; 10 AM and 2 PM (Tables S1 and S2), during manipulated biting behavior in comparison to healthy ant controls (Tables S3 and S4), after manipulated biting behavior in comparison to healthy ant controls (Tables S5 and S6), during manipulated biting behavior versus death after manipulated biting behavior (Tables S7 and S8), and genes that are uniquely up- or down-regulated during manipulated biting behavior (Tables S9 and S10). (PDF 554 kb) [file 12864_2015_1812_MOESM11_ESM.pdf]

## Healthy Ants at 2PM vs Healthy Ants at 10AM

**Table 1:** Overrepresentation of annotation terms within the total of 77 up-regulated *Camponotus floridanus* homologs (2-fold and significant Q<0.05, minimum expression of 4) in healthy *Camponotus castaneus* ant heads at 10 AM (Ant Controls at 10AM samples) compared to gene expression at 2 PM (Ant Controls at 2PM samples)

| PFAM Annotation term | # up-regulated proteins with annotation term | # up-regulated proteins without annotation term | # other proteins with annotation term | # other proteins without annotation term | log odds ratio | p-value (uncorrected) | p-value (corrected) |
|---|---|---|---|---|---|---|---|
| PF00650.15 CRAL/TRIO domain | 4 | 73 | 21 | 16963 | 3.790102263 | 4.51E-06 | 0.003399132 |
| PF03392.8 Insect pheromone-binding family, A10/OS-D | 3 | 74 | 9 | 16975 | 4.336819571 | 1.89E-05 | 0.007110284 |
| PF01401.13 Angiotensin-converting enzyme | 2 | 75 | 4 | 16980 | 4.729156166 | 0.000298068 | 0.037407538 |
| PF00917.21 MATH domain | 2 | 75 | 4 | 16980 | 4.729156166 | 0.000298068 | 0.037407538 |
| PF07690.11 Major Facilitator Superfamily | 5 | 72 | 116 | 16868 | 2.312355217 | 0.000217057 | 0.037407538 |
| PF03765.10 CRAL/TRIO, N-terminal domain | 2 | 75 | 4 | 16980 | 4.729156166 | 0.000298068 | 0.037407538 |

| SSP Annotation term | # up-regulated proteins with annotation term | # up-regulated proteins without annotation term | # other proteins with annotation term | # other proteins without annotation term | log odds ratio | p-value (uncorrected) | p-value (corrected) |
|---|---|---|---|---|---|---|---|
| SSP<br>Small Secreted Protein | 6 | 71 | 305 | 16679 | 1.530673538 | 0.002801902 | 0.002801902 |

| Secretome Annotation term | # up-regulated proteins with annotation term | # up-regulated proteins without annotation term | # other proteins with annotation term | # other proteins without annotation term | log odds ratio | p-value (uncorrected) | p-value (corrected) |
|---|---|---|---|---|---|---|---|
| secretome<br>Has a SignalP signal sequence | 15 | 62 | 928 | 16056 | 1.431721974 | 1.72E-05 | 1.72E-05 |

**Table 2:** Overrepresentation of annotation terms within the total of 54 down-regulated *Camponotus floridanus* homologs (2-fold and significant Q<0.05, minimum expression of 4) in healthy *Camponotus castaneus* ant heads at 10 AM (Ant Controls at 10AM samples) compared to gene expression at 2 PM (Ant Controls at 2PM samples)

| GO Annotation term | # down-regulated proteins with annotation term | # down-regulated proteins without annotation term | # other proteins with annotation term | # other proteins without annotation term | log odds ratio | p-value (uncorrected) | p-value (corrected) |
|---|---|---|---|---|---|---|---|
| GO:0055086\|nucleobase-containing small molecule metabolic process | 6 | 48 | 94 | 16913 | 3.113101512 | 7.17E-07 | 0.000408634 |
| GO:0044281\|small molecule metabolic process | 8 | 46 | 210 | 16797 | 2.632648192 | 3.92E-07 | 0.000408634 |
| GO:1901564\|organonitrogen compound metabolic process | 8 | 46 | 257 | 16750 | 2.427877598 | 1.71E-06 | 0.000649606 |
| GO:0072521\|purine-containing compound metabolic process | 4 | 50 | 55 | 16952 | 3.20507927 | 3.59E-05 | 0.010217626 |
| GO:1901135\|carbohydrate derivative metabolic process | 5 | 49 | 152 | 16855 | 2.426139721 | 0.000136098 | 0.020708731 |
| GO:0044710\|single-organism metabolic process | 11 | 43 | 911 | 16096 | 1.508478333 | 0.000121765 | 0.020708731 |
| GO:0044711\|single-organism biosynthetic process | 5 | 49 | 162 | 16845 | 2.361830435 | 0.000181656 | 0.020708731 |
| GO:0016742\|hydroxymethyl-, formyl- and related transferase activity | 2 | 52 | 3 | 17004 | 5.384495063 | 9.77E-05 | 0.020708731 |
| GO:0006753\|nucleoside phosphate metabolic process | 4 | 50 | 84 | 16923 | 2.779883479 | 0.000171639 | 0.020708731 |
| GO:0009117\|nucleotide metabolic process | 4 | 50 | 83 | 16924 | 2.79191876 | 0.000164221 | 0.020708731 |
| GO:0072522\|purine-containing compound biosynthetic process | 3 | 51 | 36 | 16971 | 3.322529002 | 0.000252698 | 0.024006266 |
| GO:0016741\|transferase activity, transferring one-carbon groups | 4 | 50 | 92 | 16915 | 2.68843886 | 0.000239985 | 0.024006266 |
| GO:0008484\|sulfuric ester hydrolase activity | 2 | 52 | 7 | 17000 | 4.536961936 | 0.000348986 | 0.030603412 |
| GO:0006807\|nitrogen compound metabolic process | 9 | 45 | 739 | 16268 | 1.482219434 | 0.000515393 | 0.041967712 |
| GO:1901293\|nucleoside phosphate biosynthetic process | 3 | 51 | 54 | 16953 | 2.916002698 | 0.00077716 | 0.042076237 |
| GO:0006139\|nucleobase-containing compound metabolic process | 8 | 46 | 600 | 16407 | 1.559333842 | 0.000607937 | 0.042076237 |
| GO:0006163\|purine nucleotide metabolic process | 3 | 51 | 54 | 16953 | 2.916002698 | 0.00077716 | 0.042076237 |
| GO:0046483\|heterocycle metabolic process | 8 | 46 | 631 | 16376 | 1.50706641 | 0.000841297 | 0.042076237 |
| GO:0008152\|metabolic process | 18 | 36 | 2496 | 14511 | 1.067070351 | 0.000658862 | 0.042076237 |

| GO Annotation term | # down-regulated proteins with annotation term | # down-regulated proteins without annotation term | # other proteins with annotation term | # other proteins without annotation term | log odds ratio | p-value (uncorrected) | p-value (corrected) |
|---|---|---|---|---|---|---|---|
| GO:0019637\|organophosphate metabolic process | 4 | 50 | 130 | 16877 | 2.340443932 | 0.000848907 | 0.042076237 |
| GO:0034641\|cellular nitrogen compound metabolic process | 8 | 46 | 631 | 16376 | 1.50706641 | 0.000841297 | 0.042076237 |
| GO:0009165\|nucleotide biosynthetic process | 3 | 51 | 54 | 16953 | 2.916002698 | 0.00077716 | 0.042076237 |
| GO:0006725\|cellular aromatic compound metabolic process | 8 | 46 | 618 | 16389 | 1.528677345 | 0.000735934 | 0.042076237 |
| GO:1901657\|glycosyl compound metabolic process | 3 | 51 | 59 | 16948 | 2.827154324 | 0.000993371 | 0.045297734 |
| GO:0009116\|nucleoside metabolic process | 3 | 51 | 59 | 16948 | 2.827154324 | 0.000993371 | 0.045297734 |
| GO:1901360\|organic cyclic compound metabolic process | 8 | 46 | 652 | 16355 | 1.473044523 | 0.001036977 | 0.045467466 |
| GO:0019438\|aromatic compound biosynthetic process | 4 | 50 | 142 | 16865 | 2.251440046 | 0.0011677 | 0.049302883 |

| SSP Annotation term | # down-regulated proteins with annotation term | # down-regulated proteins without annotation term | # other proteins with annotation term | # other proteins without annotation term | log odds ratio | p-value (uncorrected) | p-value (corrected) |
|---|---|---|---|---|---|---|---|
| SSP<br>Small Secreted Protein | 5 | 49 | 306 | 16701 | 1.717256389 | 0.002969093 | 0.002969093 |

## Healthy Ants at 2PM vs Death After Manipulated Biting Behavior

**Table 3:** Overrepresentation of annotation terms within the total of 581 up-regulated *Camponotus floridanus* homologs (2-fold and significant Q<0.05, minimum expression of 4) in dead *Camponotus castaneus* ant heads after manipulation (Death After Manipulation samples) compared to gene expression in healthy *Camponotus castaneus* ant heads at the same time of day; 2 PM (Ant Controls at 2PM samples)

| GO Annotation term | # up-regulated proteins with annotation term | # up-regulated proteins without annotation term | # other proteins with annotation term | # other proteins without annotation term | log odds ratio | p-value (uncorrected) | p-value (corrected) |
|---|---|---|---|---|---|---|---|
| **Over-represented** | | | | | | | |
| GO:0003674\|molecular_function | 300 | 281 | 6002 | 10478 | 0.622612879 | 2.60E-13 | 2.96E-10 |
| GO:0005488\|binding | 213 | 368 | 4115 | 12365 | 0.553440294 | 1.05E-09 | 3.98E-07 |
| GO:0065007\|biological regulation | 66 | 515 | 804 | 15676 | 0.915774732 | 9.95E-10 | 3.98E-07 |
| GO:0005515\|protein binding | 97 | 484 | 1459 | 15021 | 0.724324024 | 4.33E-09 | 8.23E-07 |
| GO:0050789\|regulation of biological process | 63 | 518 | 779 | 15701 | 0.896628122 | 3.90E-09 | 8.23E-07 |
| GO:0050794\|regulation of cellular process | 63 | 518 | 771 | 15709 | 0.907460186 | 3.03E-09 | 8.23E-07 |
| GO:0007165\|signal transduction | 35 | 546 | 360 | 16120 | 1.05444107 | 2.54E-07 | 4.14E-05 |
| GO:0006793\|phosphorus metabolic process | 35 | 546 | 384 | 16096 | 0.988412606 | 1.43E-06 | 0.000181379 |
| GO:0006796\|phosphate-containing compound metabolic process | 35 | 546 | 382 | 16098 | 0.993758797 | 1.36E-06 | 0.000181379 |
| GO:0003700\|sequence-specific DNA binding transcription factor activity | 22 | 559 | 182 | 16298 | 1.259683973 | 1.88E-06 | 0.000194637 |
| GO:0001071\|nucleic acid binding transcription factor activity | 22 | 559 | 182 | 16298 | 1.259683973 | 1.88E-06 | 0.000194637 |
| GO:0051716\|cellular response to stimulus | 37 | 544 | 446 | 16034 | 0.89411646 | 4.68E-06 | 0.000445051 |
| GO:0043167\|ion binding | 100 | 481 | 1793 | 14687 | 0.53237547 | 8.00E-06 | 0.000701855 |
| GO:0050896\|response to stimulus | 38 | 543 | 483 | 15997 | 0.84061667 | 1.12E-05 | 0.000910377 |
| GO:0036211\|protein modification process | 30 | 551 | 344 | 16136 | 0.937628995 | 1.54E-05 | 0.001097849 |
| GO:0006464\|cellular protein modification process | 30 | 551 | 344 | 16136 | 0.937628995 | 1.54E-05 | 0.001097849 |
| GO:0043412\|macromolecule modification | 31 | 550 | 369 | 16111 | 0.90052983 | 1.98E-05 | 0.00132576 |
| GO:0051171\|regulation of nitrogen compound metabolic process | 28 | 553 | 316 | 16164 | 0.951646121 | 2.57E-05 | 0.001505275 |

| GO Annotation term | # up-regulated proteins with annotation term | # up-regulated proteins without annotation term | # other proteins with annotation term | # other proteins without annotation term | log odds ratio | p-value (uncorrected) | p-value (corrected) |
|---|---|---|---|---|---|---|---|
| GO:0019219\|regulation of nucleobase-containing compound metabolic process | 28 | 553 | 316 | 16164 | 0.951646121 | 2.57E-05 | 0.001505275 |
| GO:0016310\|phosphorylation | 23 | 558 | 235 | 16245 | 1.047090188 | 2.64E-05 | 0.001505275 |
| GO:0080090\|regulation of primary metabolic process | 28 | 553 | 324 | 16156 | 0.92614977 | 3.45E-05 | 0.001870457 |
| GO:0031323\|regulation of cellular metabolic process | 28 | 553 | 327 | 16153 | 0.916747408 | 3.89E-05 | 0.001976197 |
| GO:0006468\|protein phosphorylation | 22 | 559 | 225 | 16255 | 1.044948411 | 3.99E-05 | 0.001976197 |
| GO:0019222\|regulation of metabolic process | 28 | 553 | 333 | 16147 | 0.898193572 | 5.02E-05 | 0.002386647 |
| GO:0004672\|protein kinase activity | 22 | 559 | 231 | 16249 | 1.018261917 | 5.72E-05 | 0.002577603 |
| GO:0046872\|metal ion binding | 58 | 523 | 930 | 15550 | 0.617492878 | 5.88E-05 | 0.002577603 |
| GO:2001141\|regulation of RNA biosynthetic process | 25 | 556 | 282 | 16198 | 0.948843516 | 7.60E-05 | 0.002988165 |
| GO:0051252\|regulation of RNA metabolic process | 25 | 556 | 282 | 16198 | 0.948843516 | 7.60E-05 | 0.002988165 |
| GO:0006355\|regulation of transcription, DNA-templated | 25 | 556 | 282 | 16198 | 0.948843516 | 7.60E-05 | 0.002988165 |
| GO:0010556\|regulation of macromolecule biosynthetic process | 25 | 556 | 289 | 16191 | 0.923891654 | 9.51E-05 | 0.003011545 |
| GO:0006470\|protein dephosphorylation | 7 | 574 | 25 | 16455 | 2.082789589 | 8.21E-05 | 0.003011545 |
| GO:2000112\|regulation of cellular macromolecule biosynthetic process | 25 | 556 | 289 | 16191 | 0.923891654 | 9.51E-05 | 0.003011545 |
| GO:0010468\|regulation of gene expression | 25 | 556 | 289 | 16191 | 0.923891654 | 9.51E-05 | 0.003011545 |
| GO:0009889\|regulation of biosynthetic process | 25 | 556 | 289 | 16191 | 0.923891654 | 9.51E-05 | 0.003011545 |
| GO:0016020\|membrane | 40 | 541 | 566 | 15914 | 0.731820601 | 8.04E-05 | 0.003011545 |
| GO:0031326\|regulation of cellular biosynthetic process | 25 | 556 | 289 | 16191 | 0.923891654 | 9.51E-05 | 0.003011545 |
| GO:0043169\|cation binding | 58 | 523 | 948 | 15532 | 0.597164735 | 0.000100207 | 0.003087445 |
| GO:0004721\|phosphoprotein phosphatase activity | 7 | 574 | 27 | 16453 | 2.005706997 | 0.00012368 | 0.003615268 |
| GO:0060255\|regulation of macromolecule metabolic process | 25 | 556 | 299 | 16181 | 0.889256951 | 0.000140394 | 0.00400124 |
| GO:0004725\|protein tyrosine phosphatase activity | 6 | 575 | 19 | 16461 | 2.201699675 | 0.000154781 | 0.004303654 |
| GO:0008150\|biological_process | 184 | 397 | 4060 | 12420 | 0.34912458 | 0.00016607 | 0.004507602 |
| GO:0043565\|sequence-specific DNA binding | 17 | 564 | 170 | 16310 | 1.061894351 | 0.000229955 | 0.005957921 |

| GO Annotation term | # up-regulated proteins with annotation term | # up-regulated proteins without annotation term | # other proteins with annotation term | # other proteins without annotation term | log odds ratio | p-value (uncorrected) | p-value (corrected) |
|---|---|---|---|---|---|---|---|
| GO:0009987\|cellular process | 109 | 472 | 2185 | 14295 | 0.412662894 | 0.000250437 | 0.006344406 |
| GO:0016311\|dephosphorylation | 7 | 574 | 31 | 16449 | 1.867313512 | 0.000257864 | 0.006390537 |
| GO:0005575\|cellular_component | 106 | 475 | 2120 | 14360 | 0.413154765 | 0.000270644 | 0.00656455 |
| GO:0016773\|phosphotransferase activity, alcohol group as acceptor | 23 | 558 | 285 | 16195 | 0.851103905 | 0.000398331 | 0.009460357 |
| GO:0005509\|calcium ion binding | 13 | 568 | 115 | 16365 | 1.180795997 | 0.000426716 | 0.009927668 |
| GO:0006811\|ion transport | 17 | 564 | 184 | 16296 | 0.981898293 | 0.000534317 | 0.012090622 |
| GO:0044765\|single-organism transport | 30 | 551 | 424 | 16056 | 0.723567008 | 0.000540896 | 0.012090622 |
| GO:0022838\|substrate-specific channel activity | 11 | 570 | 90 | 16390 | 1.256875913 | 0.000644986 | 0.013368804 |
| GO:0022803\|passive transmembrane transporter activity | 11 | 570 | 90 | 16390 | 1.256875913 | 0.000644986 | 0.013368804 |
| GO:0015267\|channel activity | 11 | 570 | 90 | 16390 | 1.256875913 | 0.000644986 | 0.013368804 |
| GO:0005216\|ion channel activity | 11 | 570 | 90 | 16390 | 1.256875913 | 0.000644986 | 0.013368804 |
| GO:0016301\|kinase activity | 22 | 559 | 277 | 16203 | 0.833827163 | 0.000951108 | 0.019022159 |
| GO:0006810\|transport | 36 | 545 | 564 | 15916 | 0.622759064 | 0.001163246 | 0.022476278 |
| GO:0051234\|establishment of localization | 36 | 545 | 566 | 15914 | 0.61909357 | 0.001189083 | 0.022592586 |
| GO:0016791\|phosphatase activity | 8 | 573 | 56 | 16424 | 1.409703093 | 0.001423183 | 0.026597182 |
| GO:0055085\|transmembrane transport | 21 | 560 | 273 | 16207 | 0.800312385 | 0.00157278 | 0.028918857 |
| GO:0006461\|protein complex assembly | 6 | 575 | 33 | 16447 | 1.648780236 | 0.001903759 | 0.034448974 |

| PFAM Annotation term | # up-regulated proteins with annotation term | # up-regulated proteins without annotation term | # other proteins with annotation term | # other proteins without annotation term | log odds ratio | p-value (uncorrected) | p-value (corrected) |
|---|---|---|---|---|---|---|---|
| PF00069.20 Protein kinase domain | 21 | 560 | 216 | 16264 | 1.038016602 | 6.40E-05 | 0.016065116 |
| PF13895.1 Immunoglobulin domain | 13 | 568 | 93 | 16387 | 1.394472062 | 6.33E-05 | 0.016065116 |
| PF07714.12 Protein tyrosine kinase | 21 | 560 | 213 | 16267 | 1.052187283 | 5.32E-05 | 0.016065116 |
| PF14259.1 RNA recognition motif (a.k.a. RRM, RBD, or RNP domain) | 11 | 570 | 80 | 16400 | 1.375268891 | 0.000260676 | 0.024536104 |
| PF00102.22 Protein-tyrosine phosphatase | 6 | 575 | 19 | 16461 | 2.201699675 | 0.000154781 | 0.024536104 |
| PF13306.1 Leucine rich repeats (6 copies) | 7 | 574 | 31 | 16449 | 1.867313512 | 0.000257864 | 0.024536104 |
| PF00036.27 EF hand | 6 | 575 | 20 | 16460 | 2.150345629 | 0.000195466 | 0.024536104 |
| PF00076.17 RNA recognition motif. (a.k.a. RRM, RBD, or RNP domain) | 12 | 569 | 93 | 16387 | 1.312670339 | 0.000236687 | 0.024536104 |
| PF07716.10 Basic region leucine zipper | 5 | 576 | 14 | 16466 | 2.32332585 | 0.000351943 | 0.029445866 |
| PF07647.12 SAM domain (Sterile alpha motif) | 5 | 576 | 15 | 16465 | 2.254272245 | 0.000456201 | 0.034109821 |
| PF07679.11 Immunoglobulin I-set domain | 11 | 570 | 87 | 16393 | 1.290960487 | 0.000498284 | 0.034109821 |
| PF00105.13 Zinc finger, C4 type (two domains) | 5 | 576 | 16 | 16464 | 2.189672988 | 0.000582118 | 0.036527901 |

| Secretome Annotation term | # up-regulated proteins with annotation term | # up-regulated proteins without annotation term | # other proteins with annotation term | # other proteins without annotation term | log odds ratio | p-value (uncorrected) | p-value (corrected) |
|---|---|---|---|---|---|---|---|
| secretome<br>Has a SignalP signal sequence | 48 | 533 | 895 | 15585 | 0.449920061 | 0.00536912 | 0.00536912 |

**Table 4:** Overrepresentation of annotation terms within the total of 565 down-regulated *Camponotus floridanus* homologs (2-fold and significant Q<0.05, minimum expression of 4) in dead *Camponotus castaneus* ant heads after manipulation (Death After Manipulation samples) compared to gene expression in healthy *Camponotus castaneus* ant heads at the same time of day; 2 PM (Ant Controls at 2PM samples)

| GO Annotation term | # down-regulated proteins with annotation term | # down-regulated proteins without annotation term | # other proteins with annotation term | # other proteins without annotation term | log odds ratio | p-value (uncorrected) | p-value (corrected) |
|---|---|---|---|---|---|---|---|
| GO:0008152\|metabolic process | 240 | 325 | 2274 | 14222 | 1.530063407 | 1.72E-59 | 1.96E-56 |
| GO:0008150\|biological_process | 318 | 247 | 3926 | 12570 | 1.416354973 | 6.03E-59 | 3.44E-56 |
| GO:0003824\|catalytic activity | 245 | 320 | 2519 | 13977 | 1.446488339 | 1.07E-54 | 4.07E-52 |
| GO:0003674\|molecular_function | 361 | 204 | 5941 | 10555 | 1.145480176 | 9.73E-40 | 2.77E-37 |
| GO:0016491\|oxidoreductase activity | 90 | 475 | 440 | 16056 | 1.93355803 | 5.62E-39 | 1.28E-36 |
| GO:0044699\|single-organism process | 160 | 405 | 1593 | 14903 | 1.307230252 | 1.81E-34 | 3.44E-32 |
| GO:0044710\|single-organism metabolic process | 106 | 459 | 816 | 15680 | 1.490115823 | 2.03E-30 | 3.31E-28 |
| GO:0055114\|oxidation-reduction process | 69 | 496 | 359 | 16137 | 1.83307824 | 1.93E-28 | 2.75E-26 |
| GO:0044238\|primary metabolic process | 128 | 437 | 1618 | 14878 | 0.990789862 | 1.70E-18 | 2.15E-16 |
| GO:0071704\|organic substance metabolic process | 131 | 434 | 1704 | 14792 | 0.963260855 | 5.69E-18 | 6.49E-16 |
| GO:0048037\|cofactor binding | 32 | 533 | 135 | 16361 | 1.984595433 | 6.06E-16 | 6.28E-14 |
| GO:0050662\|coenzyme binding | 26 | 539 | 99 | 16397 | 2.078114787 | 4.40E-14 | 4.18E-12 |
| GO:0005975\|carbohydrate metabolic process | 30 | 535 | 153 | 16343 | 1.790047663 | 3.75E-13 | 3.29E-11 |
| GO:0044281\|small molecule metabolic process | 32 | 533 | 186 | 16310 | 1.661001501 | 1.38E-12 | 1.13E-10 |
| GO:0044763\|single-organism cellular process | 75 | 490 | 903 | 15593 | 0.971937544 | 8.42E-12 | 6.40E-10 |
| GO:1901564\|organonitrogen compound metabolic process | 33 | 532 | 232 | 16264 | 1.469836056 | 5.95E-11 | 4.24E-09 |
| GO:0005506\|iron ion binding | 25 | 540 | 132 | 16364 | 1.747343842 | 6.76E-11 | 4.53E-09 |
| GO:0016705\|oxidoreductase activity, acting on paired donors, with incorporation or reduction of molecular oxygen | 25 | 540 | 135 | 16361 | 1.72468764 | 1.03E-10 | 6.52E-09 |
| GO:0043167\|ion binding | 114 | 451 | 1779 | 14717 | 0.737682988 | 1.43E-10 | 8.58E-09 |
| GO:1901605\|alpha-amino acid metabolic process | 12 | 553 | 20 | 16476 | 2.88347643 | 1.91E-10 | 1.09E-08 |

| GO Annotation term | # down-regulated proteins with annotation term | # down-regulated proteins without annotation term | # other proteins with annotation term | # other proteins without annotation term | log odds ratio | p-value (uncorrected) | p-value (corrected) |
|---|---|---|---|---|---|---|---|
| GO:0020037\|heme binding | 25 | 540 | 143 | 16353 | 1.666628701 | 3.00E-10 | 1.56E-08 |
| GO:0046906\|tetrapyrrole binding | 25 | 540 | 143 | 16353 | 1.666628701 | 3.00E-10 | 1.56E-08 |
| GO:0043436\|oxoacid metabolic process | 20 | 545 | 89 | 16407 | 1.911773461 | 4.16E-10 | 1.90E-08 |
| GO:0006082\|organic acid metabolic process | 20 | 545 | 89 | 16407 | 1.911773461 | 4.16E-10 | 1.90E-08 |
| GO:0019752\|carboxylic acid metabolic process | 20 | 545 | 89 | 16407 | 1.911773461 | 4.16E-10 | 1.90E-08 |
| GO:0006520\|cellular amino acid metabolic process | 18 | 547 | 72 | 16424 | 2.015755795 | 7.25E-10 | 3.18E-08 |
| GO:0044282\|small molecule catabolic process | 9 | 556 | 8 | 16488 | 3.507402864 | 8.63E-10 | 3.64E-08 |
| GO:0050660\|flavin adenine dinucleotide binding | 14 | 551 | 39 | 16457 | 2.372267072 | 1.19E-09 | 4.84E-08 |
| GO:0044765\|single-organism transport | 42 | 523 | 412 | 16084 | 1.142645073 | 1.95E-09 | 7.65E-08 |
| GO:0009987\|cellular process | 126 | 439 | 2168 | 14328 | 0.640193075 | 5.70E-09 | 2.16E-07 |
| GO:0016021\|integral component of membrane | 45 | 520 | 486 | 16010 | 1.04759386 | 7.87E-09 | 2.89E-07 |
| GO:0016740\|transferase activity | 57 | 508 | 714 | 15782 | 0.908312187 | 1.24E-08 | 4.42E-07 |
| GO:0016054\|organic acid catabolic process | 8 | 557 | 8 | 16488 | 3.387822883 | 1.40E-08 | 4.70E-07 |
| GO:0046395\|carboxylic acid catabolic process | 8 | 557 | 8 | 16488 | 3.387822883 | 1.40E-08 | 4.70E-07 |
| GO:0009063\|cellular amino acid catabolic process | 7 | 558 | 6 | 16490 | 3.540301133 | 6.07E-08 | 1.98E-06 |
| GO:0044712\|single-organism catabolic process | 13 | 552 | 49 | 16447 | 2.067479382 | 9.42E-08 | 2.98E-06 |
| GO:0016614\|oxidoreductase activity, acting on CH-OH group of donors | 14 | 551 | 62 | 16434 | 1.907295774 | 1.69E-07 | 5.20E-06 |
| GO:0003995\|acyl-CoA dehydrogenase activity | 6 | 559 | 4 | 16492 | 3.789946329 | 2.41E-07 | 7.23E-06 |
| GO:0006810\|transport | 45 | 520 | 555 | 15941 | 0.91051525 | 3.11E-07 | 9.09E-06 |
| GO:0051234\|establishment of localization | 45 | 520 | 557 | 15939 | 0.906792653 | 3.37E-07 | 9.62E-06 |
| GO:0044425\|membrane part | 46 | 519 | 591 | 15905 | 0.8693103 | 7.02E-07 | 1.95E-05 |
| GO:0006629\|lipid metabolic process | 19 | 546 | 135 | 16361 | 1.439200958 | 8.31E-07 | 2.25E-05 |
| GO:1901606\|alpha-amino acid catabolic process | 6 | 559 | 6 | 16490 | 3.384359942 | 1.00E-06 | 2.65E-05 |
| GO:0055085\|transmembrane transport | 27 | 538 | 267 | 16229 | 1.115284692 | 1.82E-06 | 4.71E-05 |

| GO Annotation term | # down-regulated proteins with annotation term | # down-regulated proteins without annotation term | # other proteins with annotation term | # other proteins without annotation term | log odds ratio | p-value (uncorrected) | p-value (corrected) |
|---|---|---|---|---|---|---|---|
| GO:1901565\|organonitrogen compound catabolic process | 9 | 556 | 27 | 16469 | 2.289854522 | 1.90E-06 | 4.82E-05 |
| GO:0044237\|cellular metabolic process | 80 | 485 | 1344 | 15152 | 0.620360038 | 2.79E-06 | 6.92E-05 |
| GO:0016787\|hydrolase activity | 66 | 499 | 1038 | 15458 | 0.67787953 | 3.08E-06 | 7.47E-05 |
| GO:0005215\|transporter activity | 32 | 533 | 360 | 16136 | 0.989918527 | 4.27E-06 | 0.000101397 |
| GO:0016773\|phosphotransferase activity, alcohol group as acceptor | 27 | 538 | 281 | 16215 | 1.063315655 | 4.39E-06 | 0.000102112 |
| GO:0016627\|oxidoreductase activity, acting on the CH-CH group of donors | 7 | 558 | 15 | 16481 | 2.623464467 | 4.65E-06 | 0.00010611 |
| GO:0006544\|glycine metabolic process | 5 | 560 | 5 | 16491 | 3.382633273 | 8.59E-06 | 0.000192023 |
| GO:0043168\|anion binding | 56 | 509 | 859 | 15637 | 0.694529932 | 9.56E-06 | 0.000209589 |
| GO:0009069\|serine family amino acid metabolic process | 5 | 560 | 6 | 16490 | 3.200251075 | 1.53E-05 | 0.000329525 |
| GO:0043169\|cation binding | 59 | 506 | 947 | 15549 | 0.649453288 | 1.70E-05 | 0.000358108 |
| GO:1901363\|heterocyclic compound binding | 100 | 465 | 1888 | 14608 | 0.509184037 | 1.77E-05 | 0.000360541 |
| GO:0097159\|organic cyclic compound binding | 100 | 465 | 1888 | 14608 | 0.509184037 | 1.77E-05 | 0.000360541 |
| GO:0036094\|small molecule binding | 54 | 511 | 839 | 15657 | 0.67907713 | 1.89E-05 | 0.00037743 |
| GO:0006869\|lipid transport | 5 | 560 | 7 | 16489 | 3.046039751 | 2.55E-05 | 0.000502142 |
| GO:1901265\|nucleoside phosphate binding | 53 | 512 | 834 | 15662 | 0.664726561 | 2.90E-05 | 0.000551072 |
| GO:0000166\|nucleotide binding | 53 | 512 | 834 | 15662 | 0.664726561 | 2.90E-05 | 0.000551072 |
| GO:0009056\|catabolic process | 15 | 550 | 122 | 16374 | 1.297560868 | 4.92E-05 | 0.000919989 |
| GO:0009071\|serine family amino acid catabolic process | 4 | 561 | 4 | 16492 | 3.380909788 | 7.49E-05 | 0.00135558 |
| GO:0006546\|glycine catabolic process | 4 | 561 | 4 | 16492 | 3.380909788 | 7.49E-05 | 0.00135558 |
| GO:0022857\|transmembrane transporter activity | 26 | 539 | 319 | 16177 | 0.894535624 | 9.66E-05 | 0.001719991 |
| GO:0005488\|binding | 184 | 381 | 4144 | 12352 | 0.364292871 | 9.90E-05 | 0.001736138 |
| GO:0008272\|sulfate transport | 4 | 561 | 5 | 16491 | 3.1577056 | 0.000131312 | 0.002169503 |
| GO:0015116\|sulfate transmembrane transporter activity | 4 | 561 | 5 | 16491 | 3.1577056 | 0.000131312 | 0.002169503 |

| GO Annotation term | # down-regulated proteins with annotation term | # down-regulated proteins without annotation term | # other proteins with annotation term | # other proteins without annotation term | log odds ratio | p-value (uncorrected) | p-value (corrected) |
|---|---|---|---|---|---|---|---|
| GO:1901682\|sulfur compound transmembrane transporter activity | 4 | 561 | 5 | 16491 | 3.1577056 | 0.000131312 | 0.002169503 |
| GO:0072348\|sulfur compound transport | 4 | 561 | 5 | 16491 | 3.1577056 | 0.000131312 | 0.002169503 |
| GO:1901575\|organic substance catabolic process | 14 | 551 | 119 | 16377 | 1.251832218 | 0.000133422 | 0.002172868 |
| GO:0044711\|single-organism biosynthetic process | 16 | 549 | 151 | 16345 | 1.148887763 | 0.000139337 | 0.00223724 |
| GO:0016310\|phosphorylation | 21 | 544 | 237 | 16259 | 0.97391493 | 0.000144568 | 0.002288993 |
| GO:0016746\|transferase activity, transferring acyl groups | 10 | 555 | 65 | 16431 | 1.516154783 | 0.000175242 | 0.002736658 |
| GO:0016301\|kinase activity | 23 | 542 | 276 | 16220 | 0.913827676 | 0.000203846 | 0.003140338 |
| GO:0006468\|protein phosphorylation | 20 | 545 | 227 | 16269 | 0.967013197 | 0.000221418 | 0.003365553 |
| GO:0019538\|protein metabolic process | 49 | 516 | 810 | 15686 | 0.609203159 | 0.000254472 | 0.003812012 |
| GO:0006820\|anion transport | 7 | 558 | 32 | 16464 | 1.864746742 | 0.000257478 | 0.003812012 |
| GO:0016616\|oxidoreductase activity, acting on the CH-OH group of donors, NAD or NADP as acceptor | 8 | 557 | 44 | 16452 | 1.680888997 | 0.000286579 | 0.004188463 |
| GO:0016742\|hydroxymethyl-, formyl- and related transferase activity | 3 | 562 | 2 | 16494 | 3.784715216 | 0.000343704 | 0.00495978 |
| GO:0008236\|serine-type peptidase activity | 11 | 554 | 88 | 16408 | 1.308918071 | 0.000430493 | 0.006058792 |
| GO:0017171\|serine hydrolase activity | 11 | 554 | 88 | 16408 | 1.308918071 | 0.000430493 | 0.006058792 |
| GO:0044283\|small molecule biosynthetic process | 6 | 559 | 25 | 16471 | 1.956090709 | 0.000466556 | 0.006486269 |
| GO:0004672\|protein kinase activity | 20 | 545 | 233 | 16263 | 0.940555893 | 0.000490512 | 0.006737146 |
| GO:0046872\|metal ion binding | 53 | 512 | 935 | 15561 | 0.543943822 | 0.000635131 | 0.008518229 |
| GO:0050661\|NADP binding | 3 | 562 | 3 | 16493 | 3.379189478 | 0.000670478 | 0.008785578 |
| GO:0005319\|lipid transporter activity | 4 | 561 | 9 | 16487 | 2.569676349 | 0.000670277 | 0.008785578 |
| GO:0046914\|transition metal ion binding | 36 | 529 | 576 | 15920 | 0.631754305 | 0.000788515 | 0.01021485 |
| GO:0005576\|extracellular region | 9 | 556 | 67 | 16429 | 1.378567009 | 0.000888917 | 0.011386127 |
| GO:0008509\|anion transmembrane transporter activity | 6 | 559 | 29 | 16467 | 1.807427823 | 0.000919415 | 0.01151795 |
| GO:1901607\|alpha-amino acid biosynthetic process | 4 | 561 | 10 | 16486 | 2.464255177 | 0.000913942 | 0.01151795 |

| GO Annotation term | # down-regulated proteins with annotation term | # down-regulated proteins without annotation term | # other proteins with annotation term | # other proteins without annotation term | log odds ratio | p-value (uncorrected) | p-value (corrected) |
|---|---|---|---|---|---|---|---|
| GO:0016772\|transferase activity, transferring phosphorus-containing groups | 30 | 535 | 450 | 16046 | 0.692897928 | 0.001011475 | 0.01253349 |
| GO:0016798\|hydrolase activity, acting on glycosyl bonds | 8 | 557 | 55 | 16441 | 1.457076611 | 0.001072976 | 0.013152608 |
| GO:0006796\|phosphate-containing compound metabolic process | 27 | 538 | 390 | 16106 | 0.728778719 | 0.001168704 | 0.014173645 |
| GO:0006793\|phosphorus metabolic process | 27 | 538 | 392 | 16104 | 0.723539433 | 0.001200915 | 0.01441098 |
| GO:0006575\|cellular modified amino acid metabolic process | 4 | 561 | 11 | 16485 | 2.368884338 | 0.001213856 | 0.014414545 |
| GO:0030170\|pyridoxal phosphate binding | 6 | 559 | 31 | 16465 | 1.740614986 | 0.001245165 | 0.0146339 |
| GO:0005575\|cellular_component | 100 | 465 | 2126 | 14370 | 0.374033201 | 0.001443121 | 0.01678733 |
| GO:0015103\|inorganic anion transmembrane transporter activity | 4 | 561 | 12 | 16484 | 2.281812298 | 0.001576411 | 0.018037545 |
| GO:0006807\|nitrogen compound metabolic process | 41 | 524 | 707 | 15789 | 0.558118491 | 0.001582241 | 0.018037545 |
| GO:0046394\|carboxylic acid biosynthetic process | 5 | 560 | 22 | 16474 | 1.899997335 | 0.00172567 | 0.019286905 |
| GO:0016053\|organic acid biosynthetic process | 5 | 560 | 22 | 16474 | 1.899997335 | 0.00172567 | 0.019286905 |
| GO:0044248\|cellular catabolic process | 10 | 555 | 90 | 16406 | 1.189209709 | 0.00175263 | 0.019398044 |
| GO:0016701\|oxidoreductase activity, acting on single donors with incorporation of molecular oxygen | 3 | 562 | 5 | 16491 | 2.868242583 | 0.001786232 | 0.019579855 |
| GO:0003854\|3-beta-hydroxy-delta5-steroid dehydrogenase activity | 4 | 561 | 13 | 16483 | 2.201708924 | 0.002007945 | 0.020809616 |
| GO:0033764\|steroid dehydrogenase activity, acting on the CH-OH group of donors, NAD or NADP as acceptor | 4 | 561 | 13 | 16483 | 2.201708924 | 0.002007945 | 0.020809616 |
| GO:0016229\|steroid dehydrogenase activity | 4 | 561 | 13 | 16483 | 2.201708924 | 0.002007945 | 0.020809616 |
| GO:0019318\|hexose metabolic process | 4 | 561 | 13 | 16483 | 2.201708924 | 0.002007945 | 0.020809616 |
| GO:0015698\|inorganic anion transport | 4 | 561 | 13 | 16483 | 2.201708924 | 0.002007945 | 0.020809616 |
| GO:0004252\|serine-type endopeptidase activity | 9 | 556 | 77 | 16419 | 1.238845341 | 0.002144514 | 0.022024743 |
| GO:0016903\|oxidoreductase activity, acting on the aldehyde or oxo group of donors | 5 | 560 | 24 | 16472 | 1.812864547 | 0.002403927 | 0.024468542 |
| GO:0006694\|steroid biosynthetic process | 4 | 561 | 14 | 16482 | 2.127540281 | 0.002514704 | 0.02514704 |
| GO:0005996\|monosaccharide metabolic process | 4 | 561 | 14 | 16482 | 2.127540281 | 0.002514704 | 0.02514704 |

| GO Annotation term | # down-regulated proteins with annotation term | # down-regulated proteins without annotation term | # other proteins with annotation term | # other proteins without annotation term | log odds ratio | p-value (uncorrected) | p-value (corrected) |
|---|---|---|---|---|---|---|---|
| GO:0008652\|cellular amino acid biosynthetic process | 4 | 561 | 15 | 16481 | 2.058486736 | 0.003102797 | 0.030493008 |
| GO:0008202\|steroid metabolic process | 4 | 561 | 15 | 16481 | 2.058486736 | 0.003102797 | 0.030493008 |
| GO:0004553\|hydrolase activity, hydrolyzing O-glycosyl compounds | 7 | 558 | 52 | 16444 | 1.378023416 | 0.003231489 | 0.031486306 |
| GO:0016853\|isomerase activity | 6 | 559 | 39 | 16457 | 1.510554548 | 0.003490194 | 0.033156846 |
| GO:0005509\|calcium ion binding | 11 | 554 | 117 | 16379 | 1.022311957 | 0.003526464 | 0.03322454 |
| GO:0009064\|glutamine family amino acid metabolic process | 3 | 562 | 7 | 16489 | 2.531649061 | 0.003642507 | 0.033759818 |
| GO:0006006\|glucose metabolic process | 3 | 562 | 7 | 16489 | 2.531649061 | 0.003642507 | 0.033759818 |
| GO:0009058\|biosynthetic process | 30 | 535 | 491 | 16005 | 0.603142959 | 0.003793597 | 0.034597603 |
| GO:0080019\|fatty-acyl-CoA reductase (alcohol-forming) activity | 4 | 561 | 16 | 16480 | 1.993887537 | 0.003778169 | 0.034597603 |
| GO:0016747\|transferase activity, transferring acyl groups other than amino-acyl groups | 6 | 559 | 40 | 16456 | 1.485175973 | 0.003903686 | 0.035319061 |
| GO:0044267\|cellular protein metabolic process | 30 | 535 | 495 | 16001 | 0.594779371 | 0.003963554 | 0.035578363 |
| GO:0016620\|oxidoreductase activity, acting on the aldehyde or oxo group of donors, NAD or NADP as acceptor | 4 | 561 | 18 | 16478 | 1.875983135 | 0.005413517 | 0.0467531 |

| PFAM Annotation term | # down-regulated proteins with annotation term | # down-regulated proteins without annotation term | # other proteins with annotation term | # other proteins without annotation term | log odds ratio | p-value (uncorrected) | p-value (corrected) |
|---|---|---|---|---|---|---|---|
| PF00067.17 Cytochrome P450 | 22 | 543 | 110 | 16386 | 1.797635358 | 3.85E-10 | 2.90E-07 |
| PF00650.15 CRAL/TRIO domain | 10 | 555 | 15 | 16481 | 2.985530259 | 3.06E-09 | 1.15E-06 |
| PF00441.19 Acyl-CoA dehydrogenase, C-terminal domain | 6 | 559 | 3 | 16493 | 4.077689035 | 9.91E-08 | 2.49E-05 |
| PF02770.14 Acyl-CoA dehydrogenase, middle domain | 6 | 559 | 4 | 16492 | 3.789946329 | 2.41E-07 | 3.63E-05 |
| PF08028.6 Acyl-CoA dehydrogenase, C-terminal domain | 5 | 560 | 1 | 16495 | 4.992313712 | 2.28E-07 | 3.63E-05 |
| PF07859.8 alpha/beta hydrolase fold | 9 | 556 | 22 | 16474 | 2.494952489 | 4.72E-07 | 4.65E-05 |
| PF07690.11 Major Facilitator Superfamily | 17 | 548 | 104 | 16392 | 1.587095848 | 4.94E-07 | 4.65E-05 |
| PF02958.15 Ecdysteroid kinase | 8 | 557 | 15 | 16481 | 2.758789582 | 4.35E-07 | 4.65E-05 |
| PF08659.5 KR domain | 12 | 553 | 50 | 16446 | 1.965363208 | 7.38E-07 | 6.17E-05 |
| PF00106.20 short chain dehydrogenase | 13 | 552 | 63 | 16433 | 1.815313372 | 1.13E-06 | 8.53E-05 |
| PF00083.19 Sugar (and other) transporter | 13 | 552 | 66 | 16430 | 1.76861078 | 1.79E-06 | 0.000122302 |
| PF02771.11 Acyl-CoA dehydrogenase, N-terminal domain | 5 | 560 | 3 | 16493 | 3.893580167 | 2.02E-06 | 0.000126605 |
| PF01370.16 NAD dependent epimerase/dehydratase family | 9 | 556 | 29 | 16467 | 2.21827411 | 3.10E-06 | 0.00017983 |
| PF00378.15 Enoyl-CoA hydratase/isomerase family | 5 | 560 | 5 | 16491 | 3.382633273 | 8.59E-06 | 0.000462047 |
| PF13561.1 Enoyl-(Acyl carrier protein) reductase | 9 | 556 | 35 | 16461 | 2.029857447 | 1.13E-05 | 0.000515536 |
| PF07914.6 Protein of unknown function (DUF1679) | 6 | 559 | 11 | 16485 | 2.777920879 | 1.16E-05 | 0.000515536 |
| PF00128.19 Alpha amylase, catalytic domain | 6 | 559 | 11 | 16485 | 2.777920879 | 1.16E-05 | 0.000515536 |
| PF03765.10 CRAL/TRIO, N-terminal domain | 4 | 561 | 2 | 16494 | 4.074178232 | 1.69E-05 | 0.000708228 |
| PF02719.10 Polysaccharide biosynthesis protein | 5 | 560 | 9 | 16487 | 2.794604022 | 6.11E-05 | 0.002422004 |
| PF01347.17 Lipoprotein amino terminal region | 4 | 561 | 4 | 16492 | 3.380909788 | 7.49E-05 | 0.002742374 |
| PF00501.23 AMP-binding enzyme | 6 | 559 | 17 | 16479 | 2.342238774 | 8.01E-05 | 0.002742374 |
| PF00732.14 GMC oxidoreductase | 6 | 559 | 17 | 16479 | 2.342238774 | 8.01E-05 | 0.002742374 |
| PF13792.1 Sulfate transporter N-terminal domain with GLY motif | 4 | 561 | 5 | 16491 | 3.1577056 | 0.000131312 | 0.003409584 |

| PFAM Annotation term | # down-regulated proteins with annotation term | # down-regulated proteins without annotation term | # other proteins with annotation term | # other proteins without annotation term | log odds ratio | p-value (uncorrected) | p-value (corrected) |
|---|---|---|---|---|---|---|---|
| PF13716.1 Divergent CRAL/TRIO domain | 5 | 560 | 11 | 16485 | 2.593812011 | 0.000126193 | 0.003409584 |
| PF00171.17 Aldehyde dehydrogenase family | 4 | 561 | 5 | 16491 | 3.1577056 | 0.000131312 | 0.003409584 |
| PF01740.16 STAS domain | 4 | 561 | 5 | 16491 | 3.1577056 | 0.000131312 | 0.003409584 |
| PF00069.20 Protein kinase domain | 20 | 545 | 217 | 16279 | 1.012680338 | 0.000127143 | 0.003409584 |
| PF00916.15 Sulfate transporter family | 4 | 561 | 5 | 16491 | 3.1577056 | 0.000131312 | 0.003409584 |
| PF07714.12 Protein tyrosine kinase | 20 | 545 | 214 | 16282 | 1.026785946 | 0.000106862 | 0.003409584 |
| PF05199.8 GMC oxidoreductase | 5 | 560 | 14 | 16482 | 2.352467954 | 0.000309354 | 0.007764787 |
| PF01130.16 CD36 family | 4 | 561 | 8 | 16488 | 2.687520036 | 0.000476464 | 0.011321638 |
| PF00135.23 Carboxylesterase family | 7 | 558 | 36 | 16460 | 1.746720722 | 0.000481132 | 0.011321638 |
| PF00370.16 FGGY family of carbohydrate kinases, N-terminal domain | 3 | 562 | 3 | 16493 | 3.379189478 | 0.000670478 | 0.014424863 |
| PF00294.19 pfkB family carbohydrate kinase | 3 | 562 | 3 | 16493 | 3.379189478 | 0.000670478 | 0.014424863 |
| PF00089.21 Trypsin | 9 | 556 | 64 | 16432 | 1.424559132 | 0.000661171 | 0.014424863 |
| PF13193.1 AMP-binding enzyme C-terminal domain | 4 | 561 | 10 | 16486 | 2.464255177 | 0.000913942 | 0.019116628 |
| PF00248.16 Aldo/keto reductase family | 3 | 562 | 4 | 16492 | 3.091446771 | 0.001144488 | 0.023291875 |
| PF00155.16 Aminotransferase class I and II | 4 | 561 | 12 | 16484 | 2.281812298 | 0.001576411 | 0.031237826 |
| PF02782.11 FGGY family of carbohydrate kinases, C-terminal domain | 3 | 562 | 5 | 16491 | 2.868242583 | 0.001786232 | 0.033625825 |
| PF01633.15 Choline/ethanolamine kinase | 3 | 562 | 5 | 16491 | 2.868242583 | 0.001786232 | 0.033625825 |
| PF06585.6 Haemolymph juvenile hormone binding protein (JHBP) | 4 | 561 | 13 | 16483 | 2.201708924 | 0.002007945 | 0.035999592 |
| PF01073.14 3-beta hydroxysteroid dehydrogenase/isomerase family | 4 | 561 | 13 | 16483 | 2.201708924 | 0.002007945 | 0.035999592 |
| PF00005.22 ABC transporter | 6 | 559 | 36 | 16460 | 1.590779532 | 0.002443631 | 0.04279195 |
| PF01061.19 ABC-2 type transporter | 4 | 561 | 14 | 16482 | 2.127540281 | 0.002514704 | 0.043035729 |

| SSP Annotation term | # down-regulated proteins with annotation term | # down-regulated proteins without annotation term | # other proteins with annotation term | # other proteins without annotation term | log odds ratio | p-value (uncorrected) | p-value (corrected) |
|---|---|---|---|---|---|---|---|
| SSP<br>Small Secreted Protein | 19 | 546 | 292 | 16204 | 0.658079605 | 0.009701656 | 0.009701656 |

| Secretome Annotation term | # down-regulated proteins with annotation term | # down-regulated proteins without annotation term | # other proteins with annotation term | # other proteins without annotation term | log odds ratio | p-value (uncorrected) | p-value (corrected) |
|---|---|---|---|---|---|---|---|
| secretome<br>Has a SignalP signal sequence | 83 | 482 | 860 | 15636 | 1.141295331 | 1.95E-16 | 1.95E-16 |

## Healthy Ants at 10AM vs Manipulated Biting Behavior

**Table 5:** Overrepresentation of annotation terms within the total of 377 up-regulated *Camponotus floridanus* homologs (2-fold and significant Q<0.05, minimum expression of 4) in live manipulated *Camponotus castaneus* ant heads (Manipulated Biting Behavior samples) compared to gene expression in healthy *Camponotus castaneus* ant heads at the same time of day; 10 AM (Ant Controls at 10AM samples)

| GO Annotation term | # up-regulated proteins with annotation term | # up-regulated proteins without annotation term | # other proteins with annotation term | # other proteins without annotation term | log odds ratio | p-value (uncorrected) | p-value (corrected) |
|---|---|---|---|---|---|---|---|
| GO:0003674\|molecular_function | 202 | 175 | 6100 | 10584 | 0.694536379 | 3.17E-11 | 3.62E-08 |
| GO:0065007\|biological regulation | 49 | 328 | 821 | 15863 | 1.060028213 | 1.59E-09 | 9.05E-07 |
| GO:0050794\|regulation of cellular process | 47 | 330 | 787 | 15897 | 1.05671239 | 3.49E-09 | 1.32E-06 |
| GO:0050789\|regulation of biological process | 47 | 330 | 795 | 15889 | 1.046095157 | 4.72E-09 | 1.34E-06 |
| GO:0019222\|regulation of metabolic process | 26 | 351 | 335 | 16349 | 1.285101795 | 1.38E-07 | 1.15E-05 |
| GO:2001141\|regulation of RNA biosynthetic process | 24 | 353 | 283 | 16401 | 1.371236463 | 9.30E-08 | 1.15E-05 |
| GO:0051252\|regulation of RNA metabolic process | 24 | 353 | 283 | 16401 | 1.371236463 | 9.30E-08 | 1.15E-05 |
| GO:0010556\|regulation of macromolecule biosynthetic process | 24 | 353 | 290 | 16394 | 1.346375544 | 1.42E-07 | 1.15E-05 |
| GO:0006355\|regulation of transcription, DNA-templated | 24 | 353 | 283 | 16401 | 1.371236463 | 9.30E-08 | 1.15E-05 |
| GO:0031323\|regulation of cellular metabolic process | 26 | 351 | 329 | 16355 | 1.303541504 | 9.91E-08 | 1.15E-05 |
| GO:2000112\|regulation of cellular macromolecule biosynthetic process | 24 | 353 | 290 | 16394 | 1.346375544 | 1.42E-07 | 1.15E-05 |
| GO:0010468\|regulation of gene expression | 24 | 353 | 290 | 16394 | 1.346375544 | 1.42E-07 | 1.15E-05 |
| GO:0009889\|regulation of biosynthetic process | 24 | 353 | 290 | 16394 | 1.346375544 | 1.42E-07 | 1.15E-05 |
| GO:0031326\|regulation of cellular biosynthetic process | 24 | 353 | 290 | 16394 | 1.346375544 | 1.42E-07 | 1.15E-05 |
| GO:0051171\|regulation of nitrogen compound metabolic process | 25 | 352 | 319 | 16365 | 1.292953733 | 2.04E-07 | 1.45E-05 |
| GO:0019219\|regulation of nucleobase-containing compound metabolic process | 25 | 352 | 319 | 16365 | 1.292953733 | 2.04E-07 | 1.45E-05 |
| GO:0060255\|regulation of macromolecule metabolic process | 24 | 353 | 300 | 16384 | 1.311863827 | 2.53E-07 | 1.56E-05 |
| GO:0036211\|protein modification process | 26 | 351 | 348 | 16336 | 1.246234375 | 2.75E-07 | 1.56E-05 |

| GO Annotation term | # up-regulated proteins with annotation term | # up-regulated proteins without annotation term | # other proteins with annotation term | # other proteins without annotation term | log odds ratio | p-value (uncorrected) | p-value (corrected) |
|---|---|---|---|---|---|---|---|
| GO:0005488\|binding | 140 | 237 | 4188 | 12496 | 0.566767582 | 3.15E-07 | 1.56E-05 |
| GO:0043412\|macromolecule modification | 27 | 350 | 373 | 16311 | 1.215920298 | 2.91E-07 | 1.56E-05 |
| GO:0016310\|phosphorylation | 21 | 356 | 237 | 16447 | 1.409429934 | 3.07E-07 | 1.56E-05 |
| GO:0006464\|cellular protein modification process | 26 | 351 | 348 | 16336 | 1.246234375 | 2.75E-07 | 1.56E-05 |
| GO:0080090\|regulation of primary metabolic process | 25 | 352 | 327 | 16357 | 1.267695698 | 3.14E-07 | 1.56E-05 |
| GO:0003700\|sequence-specific DNA binding transcription factor activity | 18 | 359 | 186 | 16498 | 1.492297136 | 6.72E-07 | 2.84E-05 |
| GO:0001071\|nucleic acid binding transcription factor activity | 18 | 359 | 186 | 16498 | 1.492297136 | 6.72E-07 | 2.84E-05 |
| GO:0006796\|phosphate-containing compound metabolic process | 27 | 350 | 390 | 16294 | 1.170309193 | 6.59E-07 | 2.84E-05 |
| GO:0006468\|protein phosphorylation | 20 | 357 | 227 | 16457 | 1.401552672 | 6.40E-07 | 2.84E-05 |
| GO:0006793\|phosphorus metabolic process | 27 | 350 | 392 | 16292 | 1.165071341 | 7.23E-07 | 2.94E-05 |
| GO:0004672\|protein kinase activity | 20 | 357 | 233 | 16451 | 1.375099583 | 9.34E-07 | 3.67E-05 |
| GO:0016301\|kinase activity | 21 | 356 | 278 | 16406 | 1.247372994 | 3.35E-06 | 0.000127159 |
| GO:0043565\|sequence-specific DNA binding | 15 | 362 | 172 | 16512 | 1.380754181 | 1.81E-05 | 0.000627713 |
| GO:0005515\|protein binding | 60 | 317 | 1496 | 15188 | 0.653153551 | 1.80E-05 | 0.000627713 |
| GO:0016773\|phosphotransferase activity, alcohol group as acceptor | 20 | 357 | 288 | 16396 | 1.159828693 | 1.82E-05 | 0.000627713 |
| GO:0008150\|biological_process | 129 | 248 | 4115 | 12569 | 0.462978289 | 3.95E-05 | 0.001325418 |
| GO:0019538\|protein metabolic process | 37 | 340 | 822 | 15862 | 0.741913491 | 0.000107687 | 0.003494178 |
| GO:0008610\|lipid biosynthetic process | 8 | 369 | 59 | 16625 | 1.809770319 | 0.000110342 | 0.003494178 |
| GO:0044267\|cellular protein metabolic process | 26 | 351 | 499 | 16185 | 0.876544385 | 0.000121136 | 0.003732313 |
| GO:0004725\|protein tyrosine phosphatase activity | 5 | 372 | 20 | 16664 | 2.415817768 | 0.000189296 | 0.005678882 |
| GO:0044255\|cellular lipid metabolic process | 8 | 369 | 66 | 16618 | 1.69723188 | 0.000222979 | 0.006517833 |

| GO Annotation term | # up-regulated proteins with annotation term | # up-regulated proteins without annotation term | # other proteins with annotation term | # other proteins without annotation term | log odds ratio | p-value (uncorrected) | p-value (corrected) |
|---|---|---|---|---|---|---|---|
| GO:0043167\|ion binding | 64 | 313 | 1829 | 14855 | 0.507247032 | 0.000475476 | 0.013551071 |
| GO:0008653\|lipopolysaccharide metabolic process | 4 | 373 | 14 | 16670 | 2.547024588 | 0.000561663 | 0.015245138 |
| GO:0009103\|lipopolysaccharide biosynthetic process | 4 | 373 | 14 | 16670 | 2.547024588 | 0.000561663 | 0.015245138 |
| GO:0006629\|lipid metabolic process | 11 | 366 | 143 | 16541 | 1.246014735 | 0.000635726 | 0.01647108 |
| GO:0006470\|protein dephosphorylation | 5 | 372 | 27 | 16657 | 2.11529302 | 0.000631971 | 0.01647108 |
| GO:0003677\|DNA binding | 23 | 354 | 464 | 16220 | 0.820313078 | 0.000746099 | 0.018901164 |
| GO:0004721\|phosphoprotein phosphatase activity | 5 | 372 | 29 | 16655 | 2.043713979 | 0.000842221 | 0.020872432 |
| GO:0046872\|metal ion binding | 38 | 339 | 950 | 15734 | 0.618703323 | 0.001073346 | 0.026034339 |
| GO:0019199\|transmembrane receptor protein kinase activity | 3 | 374 | 7 | 16677 | 2.950232146 | 0.00114436 | 0.027178541 |
| GO:0043169\|cation binding | 38 | 339 | 968 | 15716 | 0.598788546 | 0.001217949 | 0.028335946 |
| GO:0016311\|dephosphorylation | 5 | 372 | 33 | 16651 | 1.91426205 | 0.00141342 | 0.031594104 |

| PFAM Annotation term | # up-regulated proteins with annotation term | # up-regulated proteins without annotation term | # other proteins with annotation term | # other proteins without annotati on term | log odds ratio | p-value (uncorrected) | p-value (corrected) |
|---|---|---|---|---|---|---|---|
| PF07716.10 Basic region leucine zipper | 7 | 370 | 12 | 16672 | 3.268986438 | 9.75E-08 | 7.34E-05 |
| PF00170.16 bZIP transcription factor | 6 | 371 | 10 | 16674 | 3.294578213 | 7.43E-07 | 0.000267124 |
| PF00069.20 Protein kinase domain | 19 | 358 | 218 | 16466 | 1.388463858 | 1.42E-06 | 0.000267124 |
| PF07714.12 Protein tyrosine kinase | 19 | 358 | 215 | 16469 | 1.40250307 | 1.17E-06 | 0.000267124 |
| PF01094.23 Receptor family ligand binding region | 5 | 372 | 19 | 16665 | 2.46717107 | 0.000154213 | 0.023224514 |
| PF00102.22 Protein-tyrosine phosphatase | 5 | 372 | 20 | 16664 | 2.415817768 | 0.000189296 | 0.023756658 |
| PF06293.9 Lipopolysaccharide kinase (Kdo/WaaP) family | 4 | 373 | 13 | 16671 | 2.621192546 | 0.000444554 | 0.047821334 |

**Table 6:** Overrepresentation of annotation terms within the total of 391 down-regulated *Camponotus floridanus* homologs (2-fold and significant Q<0.05, minimum expression of 4) in live manipulated *Camponotus castaneus* ant heads (Manipulated Biting Behavior samples) compared to gene expression in healthy *Camponotus castaneus* ant heads at the same time of day; 10 AM (Ant Controls at 10AM samples)

| GO Annotation term | # down-regulated proteins with annotation term | # down-regulated proteins without annotation term | # other proteins with annotation term | # other proteins without annotation term | log odds ratio | p-value (uncorrected) | p-value (corrected) |
|---|---|---|---|---|---|---|---|
| GO:0008150\|biological_process | 226 | 165 | 4018 | 12652 | 1.461620564 | 1.46E-44 | 1.66E-41 |
| GO:0003824\|catalytic activity | 174 | 217 | 2590 | 14080 | 1.472255421 | 1.05E-40 | 5.97E-38 |
| GO:0008152\|metabolic process | 165 | 226 | 2349 | 14321 | 1.49314776 | 1.92E-40 | 7.31E-38 |
| GO:0003674\|molecular_function | 261 | 130 | 6041 | 10629 | 1.26200251 | 1.82E-33 | 5.19E-31 |
| GO:0044699\|single-organism process | 126 | 265 | 1627 | 15043 | 1.480727019 | 2.78E-33 | 6.34E-31 |
| GO:0044710\|single-organism metabolic process | 79 | 312 | 843 | 15827 | 1.558950328 | 5.74E-25 | 1.09E-22 |
| GO:0016491\|oxidoreductase activity | 58 | 333 | 472 | 16198 | 1.787964592 | 1.57E-23 | 2.55E-21 |
| GO:0055114\|oxidation-reduction process | 48 | 343 | 380 | 16290 | 1.791606013 | 5.17E-20 | 7.37E-18 |
| GO:0044763\|single-organism cellular process | 66 | 325 | 912 | 15758 | 1.255293021 | 1.70E-15 | 2.15E-13 |
| GO:0071704\|organic substance metabolic process | 94 | 297 | 1741 | 14929 | 0.998408613 | 3.58E-14 | 3.71E-12 |
| GO:0044238\|primary metabolic process | 91 | 300 | 1655 | 15015 | 1.012325724 | 3.55E-14 | 3.71E-12 |
| GO:0044281\|small molecule metabolic process | 27 | 364 | 191 | 16479 | 1.856251692 | 9.44E-13 | 8.97E-11 |
| GO:0044765\|single-organism transport | 38 | 353 | 416 | 16254 | 1.436527154 | 5.44E-12 | 4.77E-10 |
| GO:0005975\|carbohydrate metabolic process | 23 | 368 | 160 | 16510 | 1.863958999 | 3.51E-11 | 2.86E-09 |
| GO:1901564\|organonitrogen compound metabolic process | 27 | 364 | 238 | 16432 | 1.633398257 | 9.35E-11 | 7.11E-09 |
| GO:0006810\|transport | 41 | 350 | 559 | 16111 | 1.216746987 | 4.29E-10 | 2.92E-08 |
| GO:0009987\|cellular process | 98 | 293 | 2196 | 14474 | 0.790511282 | 4.35E-10 | 2.92E-08 |
| GO:0051234\|establishment of localization | 41 | 350 | 561 | 16109 | 1.213051408 | 4.74E-10 | 3.00E-08 |
| GO:0005506\|iron ion binding | 19 | 372 | 138 | 16532 | 1.811344615 | 3.49E-09 | 2.10E-07 |
| GO:0043167\|ion binding | 82 | 309 | 1811 | 14859 | 0.778104534 | 1.12E-08 | 6.39E-07 |

| GO Annotation term | # down-regulated proteins with annotation term | # down-regulated proteins without annotation term | # other proteins with annotation term | # other proteins without annotation term | log odds ratio | p-value (uncorrected) | p-value (corrected) |
|---|---|---|---|---|---|---|---|
| GO:0016787\|hydrolase activity | 56 | 335 | 1048 | 15622 | 0.91301775 | 1.77E-08 | 9.59E-07 |
| GO:0016021\|integral component of membrane | 35 | 356 | 496 | 16174 | 1.165001698 | 2.19E-08 | 1.14E-06 |
| GO:0043436\|oxoacid metabolic process | 15 | 376 | 94 | 16576 | 1.950877421 | 2.77E-08 | 1.26E-06 |
| GO:0006082\|organic acid metabolic process | 15 | 376 | 94 | 16576 | 1.950877421 | 2.77E-08 | 1.26E-06 |
| GO:0019752\|carboxylic acid metabolic process | 15 | 376 | 94 | 16576 | 1.950877421 | 2.77E-08 | 1.26E-06 |
| GO:0020037\|heme binding | 18 | 373 | 150 | 16520 | 1.670485091 | 6.16E-08 | 2.60E-06 |
| GO:0046906\|tetrapyrrole binding | 18 | 373 | 150 | 16520 | 1.670485091 | 6.16E-08 | 2.60E-06 |
| GO:0044425\|membrane part | 38 | 353 | 599 | 16071 | 1.060628189 | 7.46E-08 | 3.04E-06 |
| GO:0043169\|cation binding | 51 | 340 | 955 | 15715 | 0.903539624 | 9.11E-08 | 3.58E-06 |
| GO:0006869\|lipid transport | 6 | 385 | 6 | 16664 | 3.767762649 | 1.15E-07 | 4.23E-06 |
| GO:0005215\|transporter activity | 28 | 363 | 364 | 16306 | 1.239936226 | 1.15E-07 | 4.23E-06 |
| GO:0006520\|cellular amino acid metabolic process | 13 | 378 | 77 | 16593 | 2.002985939 | 1.31E-07 | 4.68E-06 |
| GO:0044712\|single-organism catabolic process | 11 | 380 | 51 | 16619 | 2.244200285 | 1.42E-07 | 4.90E-06 |
| GO:0055085\|transmembrane transport | 23 | 368 | 271 | 16399 | 1.330268093 | 3.26E-07 | 1.09E-05 |
| GO:1901605\|alpha-amino acid metabolic process | 8 | 383 | 24 | 16646 | 2.673277948 | 4.60E-07 | 1.50E-05 |
| GO:0016705\|oxidoreductase activity, acting on paired donors, with incorporation or reduction of molecular oxygen | 16 | 375 | 144 | 16526 | 1.588539574 | 8.58E-07 | 2.72E-05 |
| GO:0004576\|oligosaccharyl transferase activity | 4 | 387 | 1 | 16669 | 5.149175654 | 1.33E-06 | 4.11E-05 |
| GO:0044282\|small molecule catabolic process | 6 | 385 | 11 | 16659 | 3.161326752 | 1.39E-06 | 4.18E-05 |
| GO:0009069\|serine family amino acid metabolic process | 5 | 386 | 6 | 16664 | 3.582847057 | 2.54E-06 | 7.43E-05 |
| GO:0046872\|metal ion binding | 47 | 344 | 941 | 15729 | 0.825824226 | 2.62E-06 | 7.47E-05 |
| GO:0044237\|cellular metabolic process | 60 | 331 | 1364 | 15306 | 0.710049536 | 4.32E-06 | 0.000119991 |
| GO:0005575\|cellular_component | 83 | 308 | 2143 | 14527 | 0.602543095 | 6.16E-06 | 0.000167218 |
| GO:0005319\|lipid transporter activity | 5 | 386 | 8 | 16662 | 3.295044958 | 6.82E-06 | 0.000180696 |

| GO Annotation term | # down-regulated proteins with annotation term | # down-regulated proteins without annotation term | # other proteins with annotation term | # other proteins without annotation term | log odds ratio | p-value (uncorrected) | p-value (corrected) |
|---|---|---|---|---|---|---|---|
| GO:0009056\|catabolic process | 13 | 378 | 124 | 16546 | 1.523673256 | 1.62E-05 | 0.000419096 |
| GO:0016740\|transferase activity | 37 | 354 | 734 | 15936 | 0.819447951 | 2.08E-05 | 0.000527463 |
| GO:0016054\|organic acid catabolic process | 5 | 386 | 11 | 16659 | 2.97641116 | 2.19E-05 | 0.000530068 |
| GO:0046395\|carboxylic acid catabolic process | 5 | 386 | 11 | 16659 | 2.97641116 | 2.19E-05 | 0.000530068 |
| GO:0048037\|cofactor binding | 14 | 377 | 153 | 16517 | 1.388519653 | 3.14E-05 | 0.000745073 |
| GO:0005509\|calcium ion binding | 12 | 379 | 116 | 16554 | 1.508163297 | 3.85E-05 | 0.000895968 |
| GO:0003995\|acyl-CoA dehydrogenase activity | 4 | 387 | 6 | 16664 | 3.357116182 | 5.11E-05 | 0.001143056 |
| GO:0006544\|glycine metabolic process | 4 | 387 | 6 | 16664 | 3.357116182 | 5.11E-05 | 0.001143056 |
| GO:1901575\|organic substance catabolic process | 12 | 379 | 121 | 16549 | 1.465660855 | 5.62E-05 | 0.00121639 |
| GO:0004553\|hydrolase activity, hydrolyzing O-glycosyl compounds | 8 | 383 | 51 | 16619 | 1.917882818 | 5.66E-05 | 0.00121639 |
| GO:0071702\|organic substance transport | 12 | 379 | 125 | 16545 | 1.432895928 | 7.50E-05 | 0.001583916 |
| GO:0046914\|transition metal ion binding | 30 | 361 | 582 | 16088 | 0.831677907 | 8.88E-05 | 0.001839779 |
| GO:0016798\|hydrolase activity, acting on glycosyl bonds | 8 | 383 | 55 | 16615 | 1.842134548 | 9.12E-05 | 0.001856638 |
| GO:0019538\|protein metabolic process | 38 | 353 | 821 | 15849 | 0.731456679 | 9.59E-05 | 0.001918921 |
| GO:0055086\|nucleobase-containing small molecule metabolic process | 10 | 381 | 90 | 16580 | 1.575928476 | 9.85E-05 | 0.001936513 |
| GO:0016742\|hydroxymethyl-, formyl- and related transferase activity | 3 | 388 | 2 | 16668 | 4.165705761 | 0.000115429 | 0.002230332 |
| GO:0070085\|glycosylation | 5 | 386 | 18 | 16652 | 2.483514393 | 0.000147487 | 0.002802248 |
| GO:1901565\|organonitrogen compound catabolic process | 6 | 385 | 30 | 16640 | 2.156883468 | 0.000151722 | 0.002835465 |
| GO:0005783\|endoplasmic reticulum | 4 | 387 | 9 | 16661 | 2.951471029 | 0.000164845 | 0.002982918 |
| GO:0009063\|cellular amino acid catabolic process | 4 | 387 | 9 | 16661 | 2.951471029 | 0.000164845 | 0.002982918 |
| GO:0006820\|anion transport | 6 | 385 | 33 | 16637 | 2.061392983 | 0.000239841 | 0.004272169 |
| GO:0022857\|transmembrane transporter activity | 19 | 372 | 326 | 16344 | 0.94026388 | 0.000405491 | 0.007111682 |
| GO:0008236\|serine-type peptidase activity | 9 | 382 | 90 | 16580 | 1.467946727 | 0.000447407 | 0.007612595 |
| GO:0017171\|serine hydrolase activity | 9 | 382 | 90 | 16580 | 1.467946727 | 0.000447407 | 0.007612595 |

| GO Annotation term | # down-regulated proteins with annotation term | # down-regulated proteins without annotation term | # other proteins with annotation term | # other proteins without annotation term | log odds ratio | p-value (uncorrected) | p-value (corrected) |
|---|---|---|---|---|---|---|---|
| GO:0050662\|coenzyme binding | 10 | 381 | 115 | 16555 | 1.329297039 | 0.000609175 | 0.009999287 |
| GO:0009071\|serine family amino acid catabolic process | 3 | 388 | 5 | 16665 | 3.249235027 | 0.000613991 | 0.009999287 |
| GO:0006546\|glycine catabolic process | 3 | 388 | 5 | 16665 | 3.249235027 | 0.000613991 | 0.009999287 |
| GO:0005488\|binding | 129 | 262 | 4199 | 12471 | 0.380027448 | 0.000633388 | 0.010169897 |
| GO:0006807\|nitrogen compound metabolic process | 32 | 359 | 716 | 15954 | 0.686198208 | 0.000657242 | 0.010406333 |
| GO:0016758\|transferase activity, transferring hexosyl groups | 7 | 384 | 58 | 16612 | 1.652705191 | 0.000698182 | 0.01090311 |
| GO:0006811\|ion transport | 13 | 378 | 188 | 16482 | 1.103637354 | 0.000767556 | 0.011824511 |
| GO:0006486\|protein glycosylation | 4 | 387 | 15 | 16655 | 2.440285218 | 0.000801369 | 0.012020536 |
| GO:0043413\|macromolecule glycosylation | 4 | 387 | 15 | 16655 | 2.440285218 | 0.000801369 | 0.012020536 |
| GO:0008272\|sulfate transport | 3 | 388 | 6 | 16664 | 3.066853463 | 0.000905363 | 0.012901429 |
| GO:0015116\|sulfate transmembrane transporter activity | 3 | 388 | 6 | 16664 | 3.066853463 | 0.000905363 | 0.012901429 |
| GO:1901682\|sulfur compound transmembrane transporter activity | 3 | 388 | 6 | 16664 | 3.066853463 | 0.000905363 | 0.012901429 |
| GO:0072348\|sulfur compound transport | 3 | 388 | 6 | 16664 | 3.066853463 | 0.000905363 | 0.012901429 |
| GO:0044723\|single-organism carbohydrate metabolic process | 8 | 383 | 81 | 16589 | 1.453452502 | 0.000992824 | 0.013973075 |
| GO:0022892\|substrate-specific transporter activity | 15 | 376 | 246 | 16424 | 0.97962848 | 0.001054266 | 0.014656863 |
| GO:0008509\|anion transmembrane transporter activity | 5 | 386 | 30 | 16640 | 1.971967876 | 0.001135492 | 0.015595914 |
| GO:0008233\|peptidase activity | 19 | 372 | 355 | 16315 | 0.853267544 | 0.00122575 | 0.01663518 |
| GO:0050660\|flavin adenine dinucleotide binding | 6 | 385 | 47 | 16623 | 1.706911091 | 0.001289267 | 0.017291344 |
| GO:0016627\|oxidoreductase activity, acting on the CH-CH group of donors | 4 | 387 | 18 | 16652 | 2.257783519 | 0.001432468 | 0.018988527 |
| GO:0005576\|extracellular region | 7 | 384 | 69 | 16601 | 1.478379305 | 0.001768975 | 0.022658783 |
| GO:1901566\|organonitrogen compound biosynthetic process | 8 | 383 | 89 | 16581 | 1.358782923 | 0.001734733 | 0.022658783 |
| GO:0016614\|oxidoreductase activity, acting on CH-OH group of donors | 7 | 384 | 69 | 16601 | 1.478379305 | 0.001768975 | 0.022658783 |
| GO:0044248\|cellular catabolic process | 8 | 383 | 92 | 16578 | 1.32544977 | 0.002105411 | 0.026668541 |

| GO Annotation term | # down-regulated proteins with annotation term | # down-regulated proteins without annotation term | # other proteins with annotation term | # other proteins without annotation term | log odds ratio | p-value (uncorrected) | p-value (corrected) |
|---|---|---|---|---|---|---|---|
| GO:0070011\|peptidase activity, acting on L-amino acid peptides | 18 | 373 | 347 | 16323 | 0.819798993 | 0.002183406 | 0.027352558 |
| GO:1901606\|alpha-amino acid catabolic process | 3 | 388 | 9 | 16661 | 2.66120831 | 0.002252818 | 0.027615189 |
| GO:0016042\|lipid catabolic process | 3 | 388 | 9 | 16661 | 2.66120831 | 0.002252818 | 0.027615189 |
| GO:0019725\|cellular homeostasis | 5 | 386 | 36 | 16634 | 1.789285677 | 0.002342959 | 0.028414606 |
| GO:0051604\|protein maturation | 17 | 374 | 321 | 16349 | 0.839438436 | 0.00250631 | 0.029762433 |
| GO:0016485\|protein processing | 17 | 374 | 321 | 16349 | 0.839438436 | 0.00250631 | 0.029762433 |
| GO:0016757\|transferase activity, transferring glycosyl groups | 7 | 384 | 75 | 16595 | 1.394636207 | 0.002737391 | 0.032171395 |
| GO:0016773\|phosphotransferase activity, alcohol group as acceptor | 16 | 375 | 292 | 16378 | 0.872603144 | 0.002814561 | 0.032740814 |
| GO:1901135\|carbohydrate derivative metabolic process | 10 | 381 | 147 | 16523 | 1.08186176 | 0.003379579 | 0.03891636 |
| GO:1901607\|alpha-amino acid biosynthetic process | 3 | 388 | 11 | 16659 | 2.460417566 | 0.003602643 | 0.040663492 |
| GO:0006790\|sulfur compound metabolic process | 3 | 388 | 11 | 16659 | 2.460417566 | 0.003602643 | 0.040663492 |
| GO:0006575\|cellular modified amino acid metabolic process | 3 | 388 | 12 | 16658 | 2.37334616 | 0.004427438 | 0.049483128 |

| PFAM Annotation term | # down-regulated proteins with annotation term | # down-regulated proteins without annotation term | # other proteins with annotation term | # other proteins without annotation term | log odds ratio | p-value (uncorrected) | p-value (corrected) |
|---|---|---|---|---|---|---|---|
| PF01347.17 Lipoprotein amino terminal region | 5 | 386 | 3 | 16667 | 4.27617425 | 3.26E-07 | 0.000136976 |
| PF00067.17 Cytochrome P450 | 15 | 376 | 117 | 16553 | 1.730609756 | 3.64E-07 | 0.000136976 |
| PF07859.8 alpha/beta hydrolase fold | 7 | 384 | 24 | 16646 | 2.537138992 | 5.15E-06 | 0.000968643 |
| PF07690.11 Major Facilitator Superfamily | 13 | 378 | 108 | 16562 | 1.662790128 | 4.13E-06 | 0.000968643 |
| PF00441.19 Acyl-CoA dehydrogenase, C-terminal domain | 4 | 387 | 5 | 16665 | 3.539497746 | 3.12E-05 | 0.004705816 |
| PF02770.14 Acyl-CoA dehydrogenase, middle domain | 4 | 387 | 6 | 16664 | 3.357116182 | 5.11E-05 | 0.005500848 |
| PF00135.23 Carboxylesterase family | 7 | 384 | 36 | 16634 | 2.13095273 | 4.98E-05 | 0.005500848 |
| PF00501.23 AMP-binding enzyme | 5 | 386 | 18 | 16652 | 2.483514393 | 0.000147487 | 0.013882191 |
| PF13193.1 AMP-binding enzyme C-terminal domain | 4 | 387 | 10 | 16660 | 2.846050491 | 0.000226622 | 0.017087785 |
| PF08028.6 Acyl-CoA dehydrogenase, C-terminal domain | 3 | 388 | 3 | 16667 | 3.760180656 | 0.000226929 | 0.017087785 |
| PF00106.20 short chain dehydrogenase | 8 | 383 | 68 | 16602 | 1.629177296 | 0.000342917 | 0.022611685 |
| PF13848.1 Thioredoxin-like domain | 3 | 388 | 4 | 16666 | 3.472438583 | 0.000390374 | 0.022611685 |
| PF01395.17 PBP/GOBP family | 3 | 388 | 4 | 16666 | 3.472438583 | 0.000390374 | 0.022611685 |
| PF13405.1 EF-hand domain | 6 | 385 | 38 | 16632 | 1.920013805 | 0.000471257 | 0.023657078 |
| PF00083.19 Sugar (and other) transporter | 8 | 383 | 71 | 16599 | 1.585824407 | 0.000447046 | 0.023657078 |
| PF00128.19 Alpha amylase, catalytic domain | 4 | 387 | 13 | 16657 | 2.583506138 | 0.000510241 | 0.024013223 |
| PF02771.11 Acyl-CoA dehydrogenase, N-terminal domain | 3 | 388 | 5 | 16665 | 3.249235027 | 0.000613991 | 0.027196204 |
| PF13792.1 Sulfate transporter N-terminal domain with GLY motif | 3 | 388 | 6 | 16664 | 3.066853463 | 0.000905363 | 0.034086933 |
| PF01740.16 STAS domain | 3 | 388 | 6 | 16664 | 3.066853463 | 0.000905363 | 0.034086933 |
| PF00916.15 Sulfate transporter family | 3 | 388 | 6 | 16664 | 3.066853463 | 0.000905363 | 0.034086933 |

| SSP Annotation term | # down-regulated proteins with annotation term | # down-regulated proteins without annotation term | # other proteins with annotation term | # other proteins without annotation term | log odds ratio | p-value (uncorrected) | p-value (corrected) |
|---|---|---|---|---|---|---|---|
| SSP<br>Small Secreted Protein | 22 | 369 | 289 | 16381 | 1.217696527 | 3.15E-06 | 3.15E-06 |

| Secretome Annotation term | # down-regulated proteins with annotation term | # down-regulated proteins without annotation term | # other proteins with annotation term | # other proteins without annotation term | log odds ratio | p-value (uncorrected) | p-value (corrected) |
|---|---|---|---|---|---|---|---|
| secretome<br>Has a SignalP signal sequence | 79 | 312 | 864 | 15806 | 1.53301679 | 2.46E-24 | 2.46E-24 |

## Manipulated Biting Behavior vs Death After Manipulated Biting Behavior

**Table 7:** Overrepresentation of annotation terms within the total of 104 up-regulated *Camponotus floridanus* homologs (2-fold and significant Q<0.05, minimum expression of 4) in dead *Camponotus castaneus* ant heads after manipulation (Death After Manipulation samples) compared to gene expression in live manipulated *Camponotus castaneus* ant heads (Manipulated Biting Behavior samples)

**No overrepresented annotation terms**

**Table 8:** Over- and under-representation of annotation terms within the total of 224 down-regulated *Camponotus floridanus* homologs (2-fold and significant Q<0.05, minimum expression of 4) in dead *Camponotus castaneus* ant heads after manipulation (Death After Manipulation samples) compared to gene expression in live manipulated *Camponotus castaneus* ant heads (Manipulated Biting Behavior samples)

| GO Annotation term | # down-regulated proteins with annotation term | # down-regulated proteins without annotation term | # other proteins with annotation term | # other proteins without annotation term | log odds ratio | p-value (uncorrected) | p-value (corrected) |
|---|---|---|---|---|---|---|---|
| GO:0008152\|metabolic process | 79 | 145 | 2435 | 14402 | 1.170134439 | 1.28E-14 | 1.46E-11 |
| GO:0008150\|biological_process | 107 | 117 | 4137 | 12700 | 1.032286006 | 1.08E-13 | 4.11E-11 |
| GO:0016491\|oxidoreductase activity | 33 | 191 | 497 | 16340 | 1.737015476 | 9.30E-14 | 4.11E-11 |
| GO:0055114\|oxidation-reduction process | 28 | 196 | 400 | 16437 | 1.769915474 | 2.53E-12 | 7.20E-10 |
| GO:0003824\|catalytic activity | 78 | 146 | 2686 | 14151 | 1.0348344 | 6.90E-12 | 1.57E-09 |
| GO:0044699\|single-organism process | 56 | 168 | 1697 | 15140 | 1.089865973 | 2.09E-10 | 3.98E-08 |
| GO:0044710\|single-organism metabolic process | 38 | 186 | 884 | 15953 | 1.304784602 | 2.95E-10 | 4.81E-08 |
| GO:0003674\|molecular_function | 125 | 99 | 6177 | 10660 | 0.778859589 | 1.10E-08 | 1.56E-06 |
| GO:0048037\|cofactor binding | 13 | 211 | 154 | 16683 | 1.898284137 | 3.20E-07 | 4.06E-05 |
| GO:0050662\|coenzyme binding | 10 | 214 | 115 | 16722 | 1.916157446 | 5.92E-06 | 0.000675093 |
| GO:0016705\|oxidoreductase activity, acting on paired donors, with incorporation or reduction of molecular oxygen | 11 | 213 | 149 | 16688 | 1.755101978 | 8.75E-06 | 0.000906458 |
| GO:0004252\|serine-type endopeptidase activity | 8 | 216 | 78 | 16759 | 2.074145014 | 1.72E-05 | 0.001629806 |
| GO:0008236\|serine-type peptidase activity | 8 | 216 | 91 | 16746 | 1.91921833 | 4.78E-05 | 0.003635707 |
| GO:0005506\|iron ion binding | 10 | 214 | 147 | 16690 | 1.668741508 | 4.34E-05 | 0.003635707 |
| GO:0017171\|serine hydrolase activity | 8 | 216 | 91 | 16746 | 1.91921833 | 4.78E-05 | 0.003635707 |
| GO:0016614\|oxidoreductase activity, acting on CH-OH group of donors | 7 | 217 | 69 | 16768 | 2.059133878 | 6.21E-05 | 0.00442227 |
| GO:0020037\|heme binding | 10 | 214 | 158 | 16679 | 1.595919767 | 7.69E-05 | 0.004867452 |
| GO:0046906\|tetrapyrrole binding | 10 | 214 | 158 | 16679 | 1.595919767 | 7.69E-05 | 0.004867452 |
| GO:0016701\|oxidoreductase activity, acting on single donors with incorporation of molecular oxygen | 3 | 221 | 5 | 16832 | 3.82204879 | 0.000119119 | 0.007147118 |
| GO:0005576\|extracellular region | 6 | 218 | 70 | 16767 | 1.885937112 | 0.000486998 | 0.027758884 |

| GO Annotation term | # down-regulated proteins with annotation term | # down-regulated proteins without annotation term | # other proteins with annotation term | # other proteins without annotation term | log odds ratio | p-value (uncorrected) | p-value (corrected) |
|---|---|---|---|---|---|---|---|
| GO:0005975\|carbohydrate metabolic process | 9 | 215 | 174 | 16663 | 1.388477222 | 0.00071331 | 0.037848536 |
| GO:0004497\|monooxygenase activity | 3 | 221 | 11 | 16826 | 3.033234902 | 0.00073041 | 0.037848536 |
| GO:0016787\|hydrolase activity | 28 | 196 | 1076 | 15761 | 0.738377923 | 0.000841252 | 0.041696861 |
| GO:0051213\|dioxygenase activity | 3 | 221 | 12 | 16825 | 2.946164092 | 0.000904201 | 0.042949564 |
| GO:0006629\|lipid metabolic process | 8 | 216 | 146 | 16691 | 1.443181443 | 0.000985527 | 0.044940051 |

| PFAM Annotation term | # down-regulated proteins with annotation term | # down-regulated proteins without annotation term | # other proteins with annotation term | # other proteins without annotation term | log odds ratio | p-value (uncorrected) | p-value (corrected) |
|---|---|---|---|---|---|---|---|
| PF00089.21 Trypsin | 8 | 216 | 65 | 16772 | 2.257241972 | 5.03E-06 | 0.002527436 |
| PF07993.7 Male sterility protein | 6 | 218 | 30 | 16807 | 2.73561777 | 6.71E-06 | 0.002527436 |
| PF00550.20 Phosphopantetheine attachment site | 4 | 220 | 10 | 16827 | 3.42082174 | 2.61E-05 | 0.004916277 |
| PF02719.10 Polysaccharide biosynthesis protein | 4 | 220 | 10 | 16827 | 3.42082174 | 2.61E-05 | 0.004916277 |
| PF01370.16 NAD dependent epimerase/dehydratase family | 5 | 219 | 33 | 16804 | 2.453230853 | 0.00013155 | 0.019811459 |
| PF00266.14 Aminotransferase class-V | 3 | 221 | 7 | 16830 | 3.485457725 | 0.000250334 | 0.031416953 |
| PF00650.15 CRAL/TRIO domain | 4 | 220 | 21 | 16816 | 2.67823047 | 0.000294647 | 0.031695642 |
| PF01130.16 CD36 family | 3 | 221 | 9 | 16828 | 3.234024455 | 0.000450111 | 0.042366706 |

| SSP Annotation term | # down-regulated proteins with annotation term | # down-regulated proteins without annotation term | # other proteins with annotation term | # other proteins without annotation term | log odds ratio | p-value (uncorrected) | p-value (corrected) |
|---|---|---|---|---|---|---|---|
| SSP<br>Small Secreted Protein | 11 | 213 | 300 | 16537 | 1.046176206 | 0.002782488 | 0.002782488 |

| Secretome Annotation term | # down-regulated proteins with annotation term | # down-regulated proteins without annotation term | # other proteins with annotation term | # other proteins without annotation term | log odds ratio | p-value (uncorrected) | p-value (corrected) |
|---|---|---|---|---|---|---|---|
| secretome<br>Has a SignalP signal sequence | 50 | 174 | 893 | 15944 | 1.635218987 | 1.08E-17 | 1.08E-17 |

**Healthy Ants at 10AM vs Manipulated Biting Behavior vs Death After Manipulated Biting Behavior**

**Table 9:** Overrepresentation of annotation terms within the total of 52 *Camponotus floridanus* homologs that are up-regulated in live manipulated *Camponotus castaneus* ant heads (Manipulated Biting Behavior samples) compared to gene expression in healthy *Camponotus castaneus* ant heads sampled at the same time of day (Healthy Ant Controls at 10AM samples) and down-regulated again after manipulation (Death After Manipulation samples) (2-fold and significant Q<0.05, minimum expression of 4)

| **Secretome Annotation term** | **# up-regulated proteins with annotation term** | **# up-regulated proteins without annotation term** | **# other proteins with annotation term** | **# other proteins without annotation term** | **log odds ratio** | **p-value (uncorrected)** | **p-value (corrected)** |
|---|---|---|---|---|---|---|---|
| secretome<br>Has a SignalP signal sequence | 8 | 44 | 935 | 16074 | 1.139663717 | 0.007306394 | 0.007306394 |

**Table 10:** Overrepresentation of annotation terms within the total of 12 *Camponotus floridanus* homologs that are down-regulated in live manipulated *Camponotus castaneus* ant heads (Manipulated Biting Behavior samples) compared to gene expression in healthy *Camponotus castaneus* ant heads sampled at the same time of day (Healthy Ant Controls at 10AM samples) and up-regulated again after manipulation (Death After Manipulation samples) (2-fold and significant Q<0.05, minimum expression of 4)

**No overrepresented annotation terms**
